# Legacy and Emerging Plasticizers and Stabilizers in PVC Floorings and Implications for Recycling

**DOI:** 10.1021/acs.est.3c04851

**Published:** 2024-01-19

**Authors:** Helene Wiesinger, Christophe Bleuler, Verena Christen, Philippe Favreau, Stefanie Hellweg, Miriam Langer, Roxane Pasquettaz, Andreas Schönborn, Zhanyun Wang

**Affiliations:** † Chair of Ecological Systems Design, Institute of Environmental Engineering, ETH Zürich, 8093 Zürich, Switzerland; ‡ Service de l’air, du bruit et des rayonnements non ionisants (SABRA), Geneva Cantonal Office for the Environment, 1205 Geneva, Switzerland; § Institute for Ecopreneurship, School of Life Sciences, University of Applied Sciences and Arts Northwestern Switzerland, FHNW, 4132 Muttenz, Switzerland; ∥ National Centre of Competence in Research (NCCR) Catalysis, Institute of Environmental Engineering, ETH Zürich, 8093 Zürich, Switzerland; ⊥ Eawag—Swiss Federal Institute of Aquatic Science and Technology, 8600 Dübendorf, Switzerland; # Institute of Natural Resource Sciences, ZHAW Zurich University of Applied Science, 8820 Wädenswil, Switzerland; ¶ Empa—Swiss Federal Laboratories for Materials Science and Technology, Technology and Society Laboratory, 9014 St. Gallen, Switzerland

**Keywords:** building and construction, plastic additives, chemicals of concern, circular economy, indoor air quality, plasticizers, phthalates, recycling

## Abstract

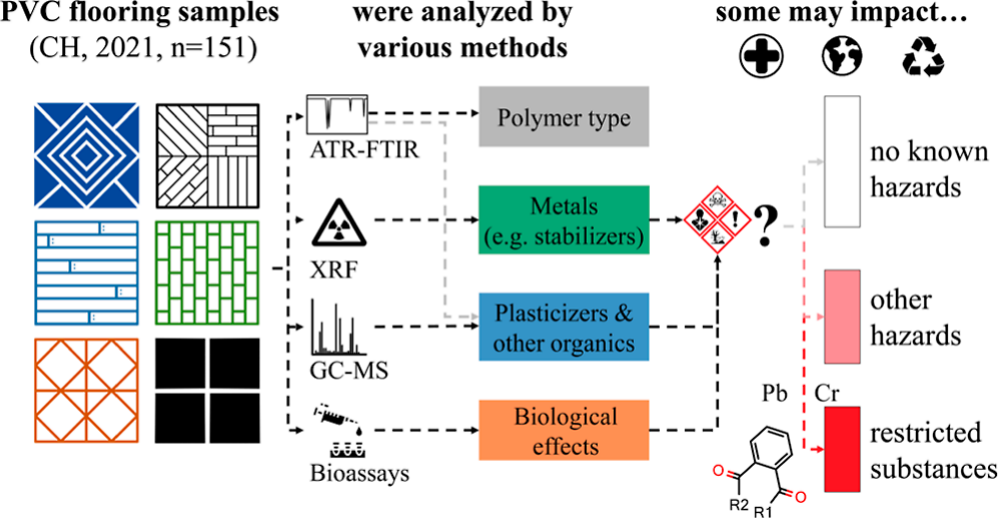

Hazardous chemicals in building and construction plastics can lead to health risks due to indoor exposure and may contaminate recycled materials. We systematically sampled new polyvinyl chloride floorings on the Swiss market (*n* = 151). We performed elemental analysis by X-ray fluorescence, targeted and suspect gas chromatography–mass spectrometry analysis of *ortho*-phthalates and alternative plasticizers, and bioassay tests for cytotoxicity and oxidative stress, and endocrine, mutagenic, and genotoxic activities (for selected samples). Surprisingly, 16% of the samples contained regulated chemicals above 0.1 wt %, mainly lead and bis(2-ethylhexyl) phthalate (DEHP). Their presence is likely related to the use of recycled PVC in new flooring, highlighting that uncontrolled recycling can delay the phase-out of hazardous chemicals. Besides DEHP, 29% of the samples contained other *ortho*-phthalates (mainly diisononyl and diisodecyl phthalates, DiNP and DiDP) above 0.1 wt %, and 17% of the samples indicated a potential to cause biological effects. Considering some overlap between these groups, they together make up an additional 35% of the samples of potential concern. Moreover, both suspect screening and bioassay results indicate the presence of additional potentially hazardous substances. Overall, our study highlights the urgent need to accelerate the phase-out of hazardous substances, increase the transparency of chemical compositions in plastics to protect human and ecosystem health, and enable the transition to a safe and sustainable circular economy.

## Abstract 2

This study highlights the importance of transparent communication and management of chemicals in plastics for the ongoing industrial transition toward a safe and sustainable circular economy.

## 1 Introduction

The building and construction sector is a major industrial user of plastics, particularly of polyvinyl chloride (PVC). In Europe, 71% of PVC is used in building and construction, contributing to 38% of all plastics used in the sector.^1,2^ Flooring is one major building and construction application (7–10% of PVC) employing primarily flexible PVC, which is also used for flexible films, sheets, cables, and tubes (14% of PVC) and is generally extensively plasticized.^3,4^ Many chemical substances are present in plastics, including residual monomers, additives, processing aids, and so-called “non-intentionally added substances” such as contaminants, by-products, and breakdown products.^5−7^ Generally, PVC requires heat- and UV-stabilization (0.05–5 wt %), flexible applications such as flooring require plasticization (5–65 wt %), and may contain large amounts of fillers (5–50 wt %), in addition to other additives (such as colorants, antioxidants).^8−10^ Among the additives, plasticizers and stabilizers are particularly interesting, as they are used in comparatively large amounts and have been the subject of regulatory scrutiny in recent years. For example, PVC plastics have been notorious for their extensive use of multiple hazardous *ortho*-phthalates as plasticizers and cadmium, lead, and tin as stabilizers. Consequently, the use patterns of plasticizers and stabilizers are changing in the PVC industry, shifting from well-known hazardous substances to alternative ones.^11,12^

Multiple *ortho*-phthalate plasticizers have been associated with various adverse health effects, including lower semen quality, altered anogenital distance, endometriosis, decreased testosterone, neurodevelopmental effects, attention-deficit hyperactivity disorder, autism, development of breast/uterine/testicular cancers, asthma, and type 2 diabetes, leading to increased regulatory scrutiny.^13,14^ For example, bis(2-ethylhexyl) phthalate [DEHP, Chemical Abstracts Service Registry Number (CASRN): 117-81-7] was added to the Authorization List of the European Union (EU)’s Chemicals Regulation, REACH, in 2011 with a sunset date in 2015, which means their use after this date is prohibited on the EU market (unless authorization has been sought and granted, which is the case for DEHP in recycled PVC).^15,16^ Similarly, benzyl butyl phthalate (BBP, CASRN: 85-68-7), di-*n*-butyl phthalate (DBP, CASRN: 84-74-2), and di-*iso*-butyl phthalate (DiBP, CASRN: 84-69-5) have also been added to the EU REACH Authorization List as well as the Swiss Chemical Risk Reduction Ordinance (ORRChem).^122^ While group-based assessment and regulation for other *ortho*-phthalates is currently being discussed on the European level.^17^ Phase-out of some *ortho*-phthalates has led to an increased demand for alternative plasticizers including terephthalates, trimellitates, cyclohexane dicarboxylic acid esters, phosphates, adipates, citrates, vegetable oil derivatives, and polymeric plasticizers (see Section S1 in the Supporting Information 1).^11,18^ Currently, these alternative plasticizers are generally less studied, and partially lack important physicochemical and toxicological data.^18^ While available hazard data indicate that many of them are likely safer than *ortho*-phthalates, some have shown some cause for concern, for example, tricresyl phosphate (CASRN: 1330-78-5, likely toxic for reproduction), and tris(2-ethylhexyl) trimellitate (TEHTM, CASRN: 3319-31-1, likely persistent and endocrine disrupting).^18−20^

Heat- and UV-stabilizers have undergone a shift in recent years. Earlier stabilizer systems were mainly based on cadmium and lead, known for posing health and environmental risks.^21,22^ The PVC industry in the EU voluntarily phased out cadmium- or lead-based stabilizer systems in 2001 and 2015, respectively, and replaced them with (organo-)tin-, barium- and zinc–calcium-based systems.^11,23,24^ Some of these replacements may lead to diverse adverse health effects. Organotins are known for their endocrine-disrupting potential, ecotoxicity, neurotoxicity, and liver toxicity.^25−27^ Barium exposure may lead to kidney diseases, neurological, cardiovascular, mental, and metabolic disorders.^28^ Zinc possesses properties indicating hazards for human health and the environment.^29^ Current regulation mainly covers legacy metal(loid) elements (chromium, cadmium, lead, arsenic, mercury, nickel), which must be below 0.1 wt % in certain plastic products.^30−33^

Building and construction plastics contribute to long-term exposure to hazardous chemicals in two ways. On the one hand, due to the long lifetime of these plastics, legacy chemicals that have been phased out from new production and use may still be common in products that are in use.^34−36^ On the other hand, the comparatively high recycling rate of building and construction PVC plastics (17% in the EU in 2012, 16% in Switzerland in 2017) and the common practice of closed-loop recycling can prolong the presence of hazardous chemicals through contamination of new products.^37,38^ This extends the consumer and occupational exposure to these substances. The EU waste framework directive aims to increase the recycling rate further, while also providing information on substances of very high concern (SVHCs) in products with the SCIP database established by the European Chemicals Agency (ECHA).^39,40^

PVC floorings have been identified as a key source of indoor chemical exposure to hazardous chemicals, especially to multiple *ortho*-phthalates and are an important source of recycled material.^41−48^ Despite that, only limited information on the chemical compositions of PVC floorings is publicly available: (1) the SCIP database contains 51 relevant entries (Sheet S11 in the Supporting Information 2), and (2) the few conducted studies have typically had a small sample size and tested a limited number of chemicals.^40,48−56^ To our knowledge, only one recent study measured many PVC flooring samples, which were from the United States market, using a nontargeted screening approach.^55^ Furthermore, various studies focused on other PVC products or indoor dust, which may allow for inferences on the possible chemical content of PVC floorings, but with significant uncertainties.^57−70^ Plasticizer handbooks suggest large variations across PVC flooring products from different times and regions, as well as across different PVC products.^3,9−12^ Furthermore, dust samples may also contain plasticizers from other products in the indoor environment. Thus, a research gap remains regarding the chemicals present in a representative selection of PVC floorings in markets other than the United States.

In this study, we aim to comprehensively understand the chemicals present in PVC floorings sold in Switzerland. Using a combination of targeted analysis and suspect screening, we present the occurrence and concentrations of legacy and novel substances in new PVC floorings from the Swiss market, focusing on plasticizers and metal heat/UV-stabilizers. In addition, we analyzed the potential biological activities of selected flooring extracts by using several bioassays. We then contextualized our observations in terms of implications for human health, the environment, and the transition to a circular economy. Finally, we provide recommendations to researchers, policymakers, industry, and citizens.

## 2 Materials and Methods

A total of 204 new flooring samples were collected from various do-it-yourself (DIY) stores and one flooring retailer for large-scale projects in Switzerland during 2021 and 2022. The samples were first screened using X-ray fluorescence (XRF) for their elemental compositions and using attenuated total reflection–Fourier transform infrared spectroscopy (ATR–FTIR) for their polymer compositions and *ortho*-phthalate presence. Only PVC samples (*n* = 151), identified by XRF and ATR–FTIR screening, were further analyzed. Targeted gas chromatography–mass spectrometry (GC–MS) was used to quantify *ortho*-phthalates. Alternative plasticizers were detected using suspect screening GC–MS. Furthermore, a selection of samples underwent several bioassays to determine potential biological activity. Detailed methods are described in the sections below.

**Figure 1:**
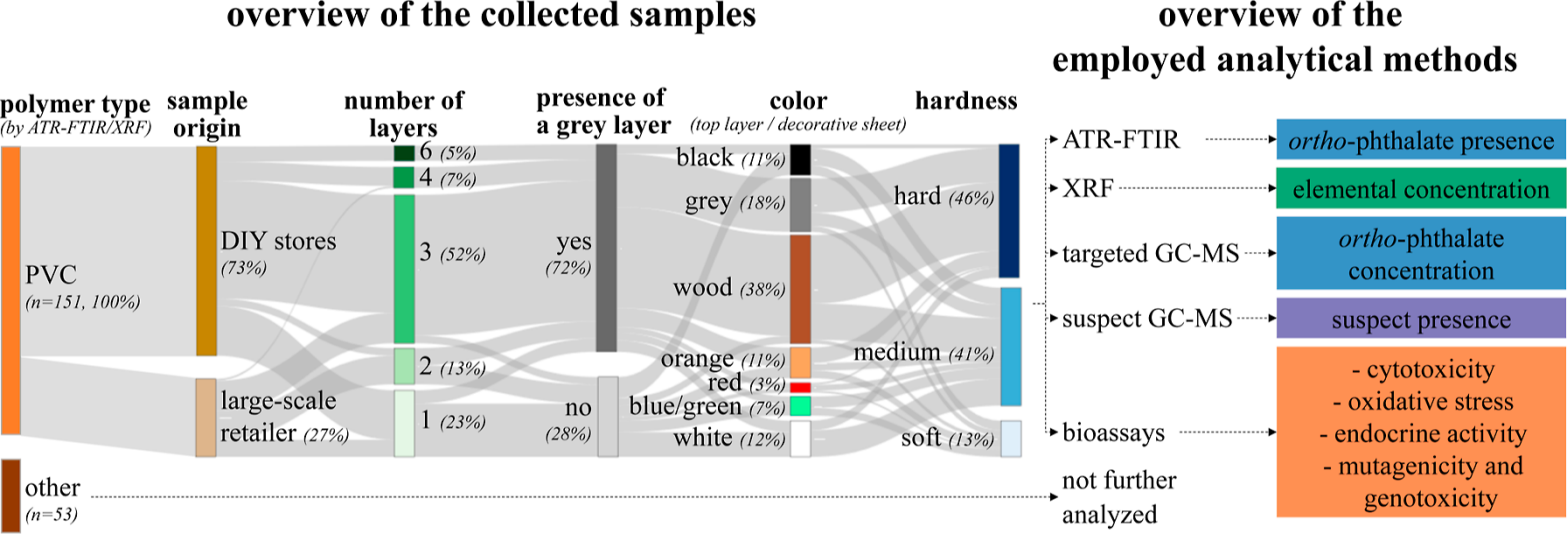
Schematic overview of the characteristics of the samples analyzed in this study and the analytical methods employed analytical methods. The presence of a gray layer may be an indication of the use of recycled PVC in the product.^71,72^ PVC = polyvinyl chloride, DIY = “do-it-yourself”, ATR-FTIR = attenuated total reflectance-Fourier transform infrared spectroscopy, XRF = X-ray fluorescence, and GC–MS = gas chromatography–mass spectrometry.

A breakdown by sample characteristics (i.e., color, hardness, number of layers, presence of a gray layer, and origin; these were manually assigned through simple visual inspection without specific testing) can be found in Figure 1. For more details on the characteristics of each sample and the assignment of characteristics, see Sheet S1 in the Supporting Information 2 and Section S2.1 in the Supporting Information 1, respectively. Information about the presence of recycled PVC in individual samples could not be obtained from the respective stores and retailers. Instead, the presence of a gray layer in a product was used as a nonconclusive proxy for recycled PVC, as color mixing and discoloration of insufficiently stabilized PVC at high temperature during recycling may cause a gray shade.^71,72^

### 2.1 Materials

An overview of the targeted compounds and the suspect list compounds and further details (e.g., CASRNs, suppliers) can be found in Table S4 in the Supporting Information 1 and Sheet S2 in the Supporting Information 2. All reagents were of analytical grade. The solutions were prepared and stored in amber glass vials. Reference materials with certified levels of metals and metalloids (ERM-EC681m) and *ortho*-phthalates (SPEX CRM-PVC001) were used as quality controls for XRF and GC–MS respectively (Table S3 in the Supporting Information 1).

### 2.2 Chemical Analysis

#### 2.2.1 ATR-FTIR Polymer Identification and *Ortho*-phthalates Screening

All samples (*n* = 204) were screened with an ATR-FTIR (ThermoScientific Nicolet iS5 with iD7 ATR module) to determine the polymer type and the presence of *ortho*-phthalates (further details in Section S2.3.1 in the Supporting Information 1, and all recorded spectra in the Supporting Information 6 − Rawdata-ATR-FTIR). Non-PVC samples (*n* = 53) were not analyzed further.

#### 2.2.2 XRF Screening of Elemental Composition

The elemental composition of all samples (*n* = 204) was determined using a hand-held XRF (Thermo Scientific Niton XL3 Gold Analyzer) with a plastic calibration (further details in Section S2.3.2 in the Supporting Information 1, and all XRF readings in Sheet S3 in the Supporting Information 2). A certified reference material (ERM-EC681m—polyethylene high level) was used to check operation and equipment calibration (measured values had to be within 20% of the certified levels).

#### 2.2.3 GC–MS Quantification of *Ortho*-phthalates

*Ortho*-phthalate quantification was performed on all PVC samples (*n* = 151) using a validated method (protocol in the Supporting Information 3) covered in the accreditation perimeter of a laboratory complying with ISO 17025:2017. The sample preparation was optimized for *ortho*-phthalate extraction and followed the validated method (the Supporting Information 3), including regular quality checks with the certified reference material (measured concentrations had to be within 20% of the certified levels). The polymer was dissolved in tetrahydrofuran (THF, CASRN: 109-99-9), followed by matrix precipitation in acetonitrile (ACN, CASRN: 75-05-8) and filtration (0.45 μm nylon filters, BGB SF2503-2). Subsequently, GC–MS analysis and quantification were carried out by using an internal standard calibration. Seventeen *ortho*-phthalates were used as standards for the calibration curve and seven deuterated *ortho*-phthalates were used as internal standards, which were added to every sample (Table S8 in the Supporting Information 1). The lowest calibration point was reported as the method limit of quantification (LOQ) for the target *ortho*-phthalates. All analyses were carried out on an Agilent GC–MS system (GC: Agilent 7890A, MS: Agilent 5975C) in single-ion mode with splitless injections. Compounds were separated on a DB-5MS column using a temperature gradient from 80 to 320 °C. Measurements were performed in batches, each containing calibration solutions, samples, blank solutions (procedural blank and solvent blank), and reference solutions (a solution with known concentration and a reference material extract). From the recorded chromatograms and mass spectra (available as Agilent files in the Supporting Information 7 − Rawdata-GCMS-Phthalates), compounds were automatically detected, identified, and quantified using weighted (1/×) quadratic calibration curves (using the quantitative Agilent Masshunter workflow in the Supporting Information 4). Further details on the *ortho*-phthalate quantification workflow are given in Section S2.3.3 in the Supporting Information 1.

#### 2.2.4 GC–MS Suspect Screening

All PVC samples (*n* = 151) were screened for other substances on our suspect list, mainly containing alternative plasticizers and antioxidants (Table S10 in the Supporting Information 1). The suspect screening was conducted with low-resolution GC–MS using electron impact ionization (EI). The method parameters were based on Löschner et al. (2011) but adapted and optimized for detecting the substances on our suspect list (Section S2.3.4 in the Supporting Information 1).^73^ Furthermore, for all substances on our suspect list, the suitability of the extraction method was checked, a custom library was created, and a dilution series for semiquantification and determination of the limits of detection (LOD) was conducted. The analyses were conducted on an Agilent GC–MS system (GC: Agilent 7890A, MS: Agilent 5975C) in scan mode with splitless injections. Generally, a nonpolar column (DB 5MS), a slow temperature gradient (8 °C/min), a high final temperature (40–300 °C), a long runtime (55 min), and a wide scan range (30–800 amu) were chosen to ensure elution, separation, and identification of all contained compounds. Measurements were performed in one batch per dilution (40×/1600×), containing all samples, regular blanks, and regular suspect standard solutions.

Recorded chromatograms and mass spectra (available as Agilent files in the Supporting Information 8-Rawdata-GCMS-Suspect) were analyzed for (1) the presence and approximate concentration of the compounds on the suspect list and (2) the presence of other unknown substances. For this, the “Agilent MassHunter—Qualitative analysis” software (workflow in the Supporting Information 4), some manual processing, and Python-based data processing (Python scripts in the Supporting Information 5) were employed. For the compound discovery, both “Find by integration” (considering all Lorentzian chromatogram peaks with an area larger than 0.001% of the largest peak) and “Find by molecular feature” (limited to Lorentzian peaks with more than 500 counts and the largest 200 compounds) were used. For the compound identification, two libraries were used, (1) a manually created custom suspect list library of the scanned suspect list standards and (2) the NIST 14 library (which contains fewer but more commonly used substances to limit overfitting the data). Results were considered acceptable if the mass error ranged from −0.3 to +0.7 Da and the matching score was over 70. Further details on the suspect screening workflow are in Section S2.3.4 in the Supporting Information 1.

### 2.3 Testing of Biological Activities

Due to time and resource constraints, only selected samples were tested using various bioassays. Randomly selected samples (*n* = 85) were tested for cytotoxicity using the MTT assay and induction of oxidative stress using the ROS assay. Several samples were selected to cover maximal differences regarding their *ortho*-phthalate contents and cytotoxicity, and were further tested for endocrine activity using the YES/YAS assays, mutagenic activity using the AMES assay, and genotoxic activity using the planar-umuC assay (for sample selection, see Table S11 in Supporting Information 1). The same extraction procedure as above was used (Section 2.2.3) except that the samples were concentrated after filtration from 6 mL to 300 μL using the Syncore system from Buchi (which avoids losses of volatile substances). This was done because most bioassays have a low solvent tolerance (MTT/ROS: maximally 0.1 vol %). Due to the high volatility of THF, the sample volumes decreased during the storage (−20 °C) and transport (max 20 °C for 2–3 days) and were filled up to 300 μL with THF before each assay. The bioassays generally used procedural blanks, solvent (negative) controls, and positive controls unless otherwise required by the respective manufacturer protocol.

#### 2.3.1 Cytotoxicity and Oxidative Stress

The highest possible test concentrations (1-μL concentrate containing the dissolved fraction of ∼1.1 mg PVC was used on 1 mL cell culture medium) of the randomly selected extracts (*n* = 85) were screened for cytotoxicity using the MTT assay and for oxidative stress using the ROS assay. Both assays were conducted on human liver cells (*Huh7*), according to Christen et al. 2014 (detailed conditions in Section S2.4 in Supporting Information 1).^74^ Samples were categorized based on their cell viability in the MTT assay (<30%: “highly toxic”; 30–60%: “moderately toxic”; 60–90%: “slightly toxic”; >90%: “not toxic”, variation of solvent control).

#### 2.3.2 Other End Points (Endocrine Activity, Mutagenicity, Genotoxicity)

Eight selected extracts were screened for potential estrogenic, antiestrogenic, androgenic, and antiandrogenic activities using the XenoScreen YES/YAS assays from Xenometrix (Allschwil, Switzerland). Five selected extracts were screened for potential mutagenic activity using the Ames MPF 98/100 assay from Xenometrix (Allschwil, Switzerland) with *Salmonella typhimurium* strains TA98 (for detection of frameshift mutations) and TA100 (for detection of base substitution mutations), following the manufacturer’s protocol which conforms with the OECD Test Guideline 471. Twelve selected extracts were screened for potential direct genotoxic activity using the planar-umuC bioassay protocol of planar4 GmbH (Stäfa, Switzerland). Further details are given in Section S2.4 in the Supporting Information 1.

## 3 Results

### 3.1 Presence and Concentrations of Chemicals Detected in the PVC Floorings

#### 3.1.1 Elemental Compositions of the Samples

**Figure 2:**
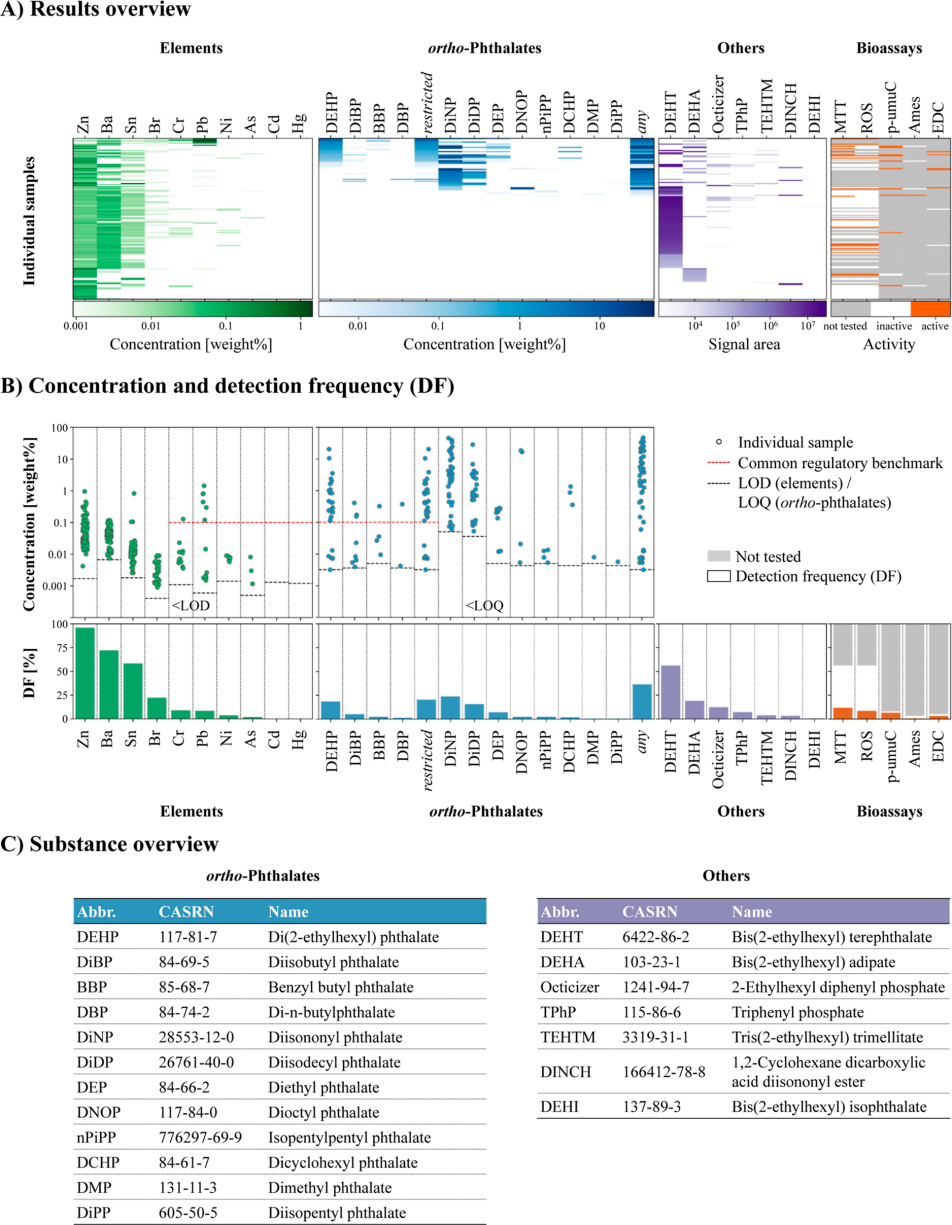
(A) Heatmap of the concentrations of selected elements and targeted *ortho*-phthalates, the presence of alternative plasticizers, and the activities in bioassay tests, with every row representing one sample. (B) Measured concentrations (top) and DF, (bottom) of selected elements and targeted *ortho*-phthalates, alternative plasticizers, and bioassay tests. Note that the DF are calculated in the following ways: elements had to be above the LOD (0.0004–0.0067 wt %) and *ortho*-phthalates above the LOQ (∼0.05 wt % for DiNP and DiDP, ∼0.005 wt % for the others), whereas other substances were considered “detected” when they were (tentatively) identified in the suspect screening workflow. DF = detection frequency, LOD = limit of detection, LOQ = limit of quantification, and *p*-umuC = planar-umuC.

The detection frequencies (DFs) and concentration ranges of various elements in the 151 PVC samples are shown in Figure 2, and details are given in Sheet S1 in the Supporting Information 2. The most prevalent elements besides chlorine (which is part of the PVC matrix) are zinc (DF: 96%), iron (DF: 76%), barium (DF: 72%), titanium (DF: 68%), tin (DF: 58%), and vanadium (DF: 46%). Surprisingly, also several potentially toxic metals and metalloids including chromium, lead, arsenic, and nickel are detected in 29 samples (DF: 19%; range 0.001–1.562 wt %), with six samples surpassing a common regulatory reference level of 0.1 wt %, most of which contain lead (Figure 4). None of the samples contain cadmium or mercury.

**Figure 3:**
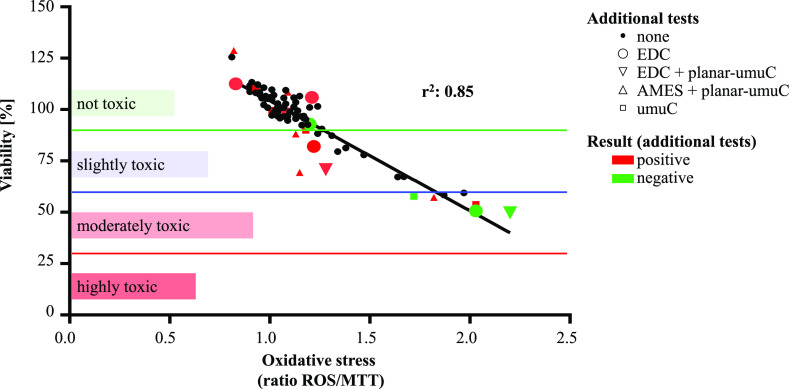
Biological activities of the tested samples (*n* = 85). All markers show the results of cytotoxicity (*y*-axis) against oxidative stress (*x*-axis) in Huh7 cells after 24 h exposure, with the mean values of three independent experiments shown here. Special markers signify if samples were tested in additional bioassays, and the marker color signifies the result of the additional tests.

**Figure 4:**
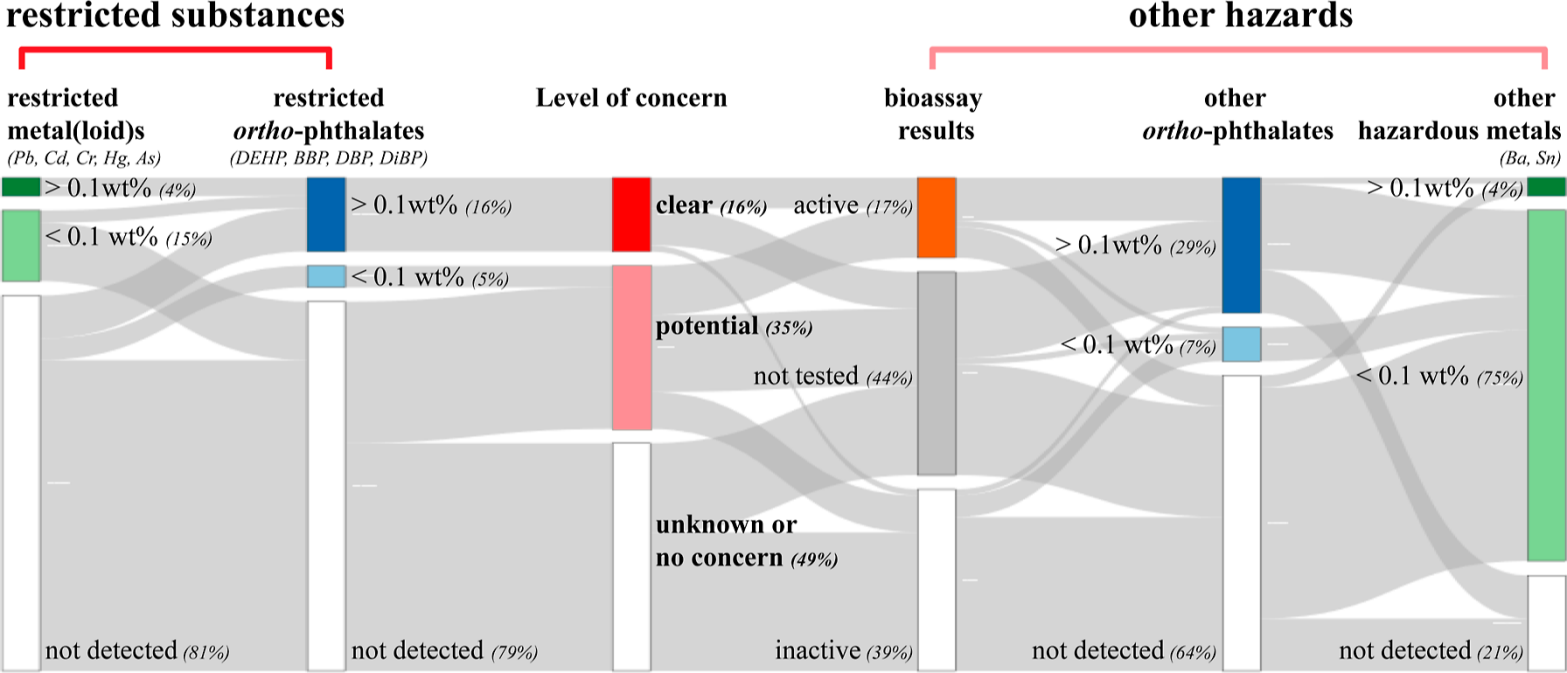
Percentage of samples with a reason for clear or potential concern. DEHP = di(2-ethylhexyl) phthalate (CASRN: 117-81-7); BBP = benzyl butyl phthalate (CASRN: 85-68-7); DBP = di-*n*-butyl phthalate (CASRN: 84-74-2); DiBP = diisobutyl phthalate (CASRN: 84-69-5).

Correlations between some elements and the product color are observed. For example, titanium concentrations strongly correlate with white color. Also, the concentrations of the toxic metals and metalloids correlate positively with the presence of a gray layer (which may indicate recycled PVC) and negatively with the number of layers (see Section S3.5 in the Supporting Information 1).^71,72^ Cadmium and lead had been the major heat stabilizers before they were voluntarily phased out by the PVC industry in the EU (cadmium in 2000 and lead in 2015). Today, heat stabilization for PVC in the EU is achieved mainly using zinc–calcium, zinc–tin, and zinc–barium systems.^11,24^ The observed elemental compositions provide supporting evidence for this industrial shift. In particular, no samples contain cadmium, indicating its phase-out in new products. Instead, most samples contain zinc, tin, and/or barium, suggesting the wide use of novel heat-stabilization systems (noting that calcium, another substance commonly used in novel heat-stabilizer systems, was not measured in this study). Meanwhile, the presence of lead in several samples (DF: 9%) is most likely associated with recycled PVC in products (see Section S3.5 in the Supporting Information 1).

#### 3.1.2 Plasticizers

The *ortho*-phthalate quantification with GC–MS showed that 55 samples (DF: 36%) contain *ortho*-phthalates, ranging from 0.01–47 wt % (Figure 2 and Table S13 in the Supporting Information 1), most of which were also captured by ATR-FTIR screening with a sensitivity of 78% and a specificity of 85% (see Section S2.3.1 in Supporting Information 1). The most prevalent *ortho*-phthalates are DiNP (diisonoyl phthalate; CASRN: 68515-48-0; DF: 24%; 0.05–47 wt %), DEHP (DF: 19%; 0.003–20 wt %), and DiDP (diisodecyl phthalate; CASRN: 68515-49-1; DF: 16%; 0.05–28 wt %). This is despite DiDP and DiNP having a LOQ approximately ten times higher (∼0.05 wt %) than the other *ortho*-phthalates (∼0.005 wt %). They were mostly found in soft or medium-hard products (Section S3.5 in Supporting Information 1). DBP, DiBP, BBP, and DEHP are regulated under the EU REACH Authorization List and the Swiss ORRChem , which means that their use is prohibited on the Swiss and the common EU market and new products shall not contain more than 0.1 wt % of these substances (unless an authorization has been sought and granted).^15,122^ In the case of DEHP, specific authorization for recycled soft PVC was granted in 2016 and is now expired.^16^ Overall, 31 samples (DF: 21%) contain these restricted *ortho*-phthalates (mainly DEHP), ranging from 0.003 to 21 wt %, with 24 samples surpassing the 0.1 wt % threshold.

In addition to *ortho*-phthalates, the qualitative suspect screening shows the presence of alternative plasticizers in 123 samples (DF: 81%, see Figure 2, Sheet S1 in the Supporting Information 2); the most frequently detected ones are DEHT [Bis(2-ethylhexyl) terephthalate; CASRN: 6422-86-2; DF: 56%], DEHA [Bis(2-ethylhexyl) adipate, CASRN: 103-23-1; DF: 19%], and Octicizer [2-ethylhexyl diphenyl phosphate; CASRN: 1241-94-7; DF: 13%]. Most alternative plasticizers were confirmed using corresponding analytical standards and semiquantified (Table S14 in the Supporting Information 1). However, semiquantification results remain highly uncertain as no internal standard was used and some signals were beyond the calibration range (even leading to implausible concentration estimates above 100 wt %; see Figure S9 in the Supporting Information 1). With these uncertainties in mind, DINCH [1,2-cyclohexane dicarboxylic acid diisononyl ester; CASRN: 166412-78-8] and DEHT are present in high concentrations. The overall estimated plasticizer composition per sample can be found in Figure S9 in the Supporting Information 1. Alternative plasticizers are more common in hard PVC samples with many layers (Section S3.5 in the Supporting Information 1).

The observed plasticizer profiles in Figure 2 visualize the ongoing industrial shift from legacy *ortho*-phthalates such as DBP, DiBP, BBP, and DEHP to an increased use of other *ortho*-phthalates (mainly DiNP and DiDP) and alternative plasticizers (mainly DEHT, DEHA, and Octicizer).^18^ Interestingly, samples typically contain one major plasticizer, either DiNP/DiDP or an alternative plasticizer (Figure S9 in the Supporting Information 1). DEHP was generally present along with other major plasticizers and at concentrations below the usual plasticizer range for flexible PVC (5–65 wt %).^9^ This suggests that the presence of DEHP comes mainly from recycling rather than intentional use.

#### 3.1.3 Other Substances Detected in the Suspect Screening

In total, nearly 400 substances are tentatively identified using chromatogram integration and NIST library matching, mostly without further confirmation (Sheet S9 in the Supporting Information 2). Some more frequently detected substances identified through library matches include oleamide (DF: 21%, CASRN: 301-02-0), 5-hexen-1-ol (DF: 11%, CASRN: 821-41-0), dodecane (DF: 11%, CASRN: 112-40-3), hexanamide (DF: 10%, CASRN: 628-02-4), and isobutyric anhydride (DF: 10%, CASRN: 97-72-3). Some of the tentatively identified substances are or may be hazardous. For example, endocrine-disrupting bisphenol A (CASRN: 80-05-7) was present in two samples (confirmed using an analytical standard, DF: 1%), possibly persistent, bioaccumulative, and toxic UV-326 (bumetrizole, CASRN: 3896-11-5) was likely present in the six samples (library match, DF: 4%) and short-chain chlorinated paraffins (SCCPs, matched by CASRN: 111-85-3 and CASRN: 73772-39-1) were likely present in the two samples (DF: 1%).

### 3.2 Bioassays

From the 85 tested samples, 26 show some biological activities (DF: 17%; Figures 2 and 3):

(1) Seven of the 85 tested samples show moderate cytotoxicity and clear induction of oxidative stress (i.e., the ratio of oxidative stress/viability >1.5), whereas another 11 samples display slight cytotoxicity. There is a clear correlation between cell viability and ROS (Figure 3).
(2) Endocrine activities are observed in five of the eight tested samples and show no correlation with the cytotoxicity (Figure 3).
(3) For one of the five tested samples, mutagenic potential cannot be ruled out (Figure S13 in the Supporting Information 1).
(4) Genotoxic activity in the planar-umuC assay is observed in 11 of the 12 tested samples (Figure S14 in the Supporting Information 1), with one showing activity in the 1:1000 dilution, five showing activity in the 1:100 dilution, and another five showing activity in the 1:10 dilution.

Generally, these observed biological activities do not correlate with the product characteristics such as color, hardness, or presence of a gray layer, nor the content of specific chemicals detected in this study (except for endocrine activity, which only occurred in samples containing *ortho*-phthalates, see Figure 2; however, this result needs to be read with caution, as only eight samples were tested). Furthermore, although cytotoxicity and oxidative stress correlated strongly, they could not be used to predict other biological activities (Figure 3).

## 4 Discussion

### 4.1 Comparison with Previous Studies

Over time, the dominant use of DEHP has been replaced by other *ortho*-phthalates (e.g., DiNP, DiDP) and alternative plasticizers (e.g., DEHT, DINCH, DEHA, and Octicizer), as demonstrated by the changing substances reported in this study and previous studies on PVC flooring (see Table S24 in the Supporting Information 1).^48−54^ Meanwhile, some regional differences in this industrial transition, especially in alternative plasticizer use, can be observed. For example, the major emerging plasticizers identified in Switzerland in this study are DiDP, DiNP, DEHT, and Octicizer. However, a study in Norway that focused on phosphor plasticizers and flame retardants mainly found TBEP [tris(2-butoxyethyl) phosphate, CASRN: 78-51-3] and TPhP (triphenyl phosphate, CASRN: 115-86-6).^52^ A nontargeted study in the United States found DEHA, DEP, DBP, BBP, TXIB (2,2,4-trimethyl-1,3-pentandiol diisobutyrate, CASRN: 6846-50-0), and ATBC (acetyltributyl citrate, CASRN: 77-90-7).^55^ Note that these differences could also be due to different target substances, instrumentation, and/or extraction procedures.

Other products made from PVC, mainly medical devices and toys, have frequently been studied for their plasticizer content (Table S26 in the Supporting Information 1). For PVC medical devices, DEHP has been present in high concentrations (up to 40 wt %) and has only partially been replaced with alternatives (e.g., DiNP, DEHT, DINCH, TEHTM, and ATBC) in recent years.^57,58,63−70^ The use of DEHP in medical devices in the EU had still been specifically authorized until recently, which may explain these findings.^70,75^ For PVC toys, due to increased regulatory scrutiny in the sector, DEHP and other commonly restricted *ortho*-phthalates have been replaced with alternatives comparatively early on [mainly with ATBC, DEHT, TXIB, DINCH, ESBO (epoxidized soybean oil, CASRN: 8013-07-8)].^76−82^ However, DEHP and other commonly restricted *ortho*-phthalates are still widely found in many PVC toys across the globe (present in 11 of 118 toys in Switzerland, 89 of 700 in the EU, 17 of 49 in New Zealand, and 1 of 1 in Jordan).^79−82^ The wide presence of such well-known hazardous substances across a wide range of PVC products points to issues in monitoring and enforcement of existing regulations and may pose a risk of contamination to any PVC product, including flooring, should open-loop recycling occur.

To the best of our knowledge, metals have not explicitly been studied in PVC floorings but only in other PVC products (Table S25 in the Supporting Information 1). Similarly to this study, barium and tin have often been found to be the main heat-stabilizers.^83−85^ Some studies have also reported the presence of lead and cadmium, which are not detected (cadmium) or only detected in a few samples (lead) in this study.^85−87^ This may indicate an ongoing industrial transition to alternative heat-stabilizers.

### 4.2 Implications of Our Findings on Human Health and the Environment

#### 4.2.1 Restricted Substances

A wide range of chemicals was detected in this study, with many quantified as well. Many of them are hazardous or potentially hazardous substances. Using a simple common regulatory threshold of 0.1 wt % (e.g., used as a threshold under the EU Restriction of Hazardous Substances in Electrical and Electronic Equipment (RoHS) Directive, and as a reporting threshold for SVHCs in articles under the EU REACH), 16% of the samples show a clear cause for concern. These contain restricted *ortho*-phthalates (16%), and some additionally contain lead (4%) and/or chromium (0.5%) (Figure 4).

#### 4.2.2 Other Hazards

Meanwhile, there could be more samples of potential concern in addition to those samples showing a clear cause for concern. First, an additional 11% of the samples showed activity in one of the bioassays, indicating the potential to cause biological effects. Second, additional 16% of the samples containing some other hazardous plasticizers (several *ortho*-phthalates) and stabilizers (barium and tin) above the common threshold of 0.1 wt % and thus may be of potential concern due to the toxicity of these chemicals.^13,14,25−28^ Third, some samples contain the already restricted substances below the common threshold of 0.1 wt % and are thus not counted as of clear concern. However, several of these chemicals are endocrine disrupting or genotoxic and thus may have a safety threshold below 0.1 wt % (e.g., lead has no safety threshold). Therefore, these samples may still be of potential concern, accounting for an additional 16% of the samples. In total, 16% of samples show a clear reason for concern and 35% of samples are of potential concern (Figure 4).

Furthermore, this does not imply that the other samples are guaranteed to be entirely safe. For example, while currently available evidence suggests that the detected alternative plasticizers may be safer than restricted *ortho*-phthalates, many are present in high concentrations in the samples (especially DINCH and DEHT) and their continued release may cause significant exposure and render them ubiquitous in the environment.^18,88^ Furthermore, research on the environmental and human health effects of alternative plasticizers and stabilizers is ongoing, which may warrant further assessment in the future.^18,25−28,89^ In addition, some other hazardous substances could have been present in the samples but are not detected/quantified in this study, chemically and/or through bioassays.

#### 4.2.3 Human Exposure Potential from PVC Floorings

While many substances are detected in PVC floorings, one may question whether they can be released from the products and result in actual exposure.

For metals and metalloids, it is not an easy question to answer, as release depends on their metal(loid) form (e.g., chemical species, matrix, particle size) and several other environmental variables (e.g., environmental pH, exposure route).^90^ Previous studies have demonstrated the release of lead from PVC during use and associated toxic effects on human health.^91,92^ Thus, the continued presence of toxic metals in PVC products may to a certain extent pose a risk to humans and the environment.

For plasticizers, literature on exposure from PVC flooring and other sources has been abundant (see Section S4.3 in the Supporting Information 1), with the following learnings that are relevant to our results. For *ortho*-phthalates, PVC floorings are a major contributor to indoor air and dust concentrations and are responsible for a large portion of total indoor exposure (low μg kg_bw_^–1^ d^–1^ range; ingestion or inhalation of dust, inhalation of airborne particles, and direct skin contact being the major exposure pathways).^41−48^ Together with dietary intake (which is the main exposure pathway, higher μg kg_bw_^–1^ d^–1^), occupational exposure, and, for some individuals, medical exposure (low mg kg_bw_^–1^ d^–1^), relevant health limit values can be approached or even exceeded, especially for susceptible populations (e.g., toddlers).^41−44,70^ Ongoing exposure is a particular concern, as recent meta-reviews suggest that “safe levels” for typical health concerns posed by some *ortho*-phthalates (e.g., endocrine disruption, developmental toxicity) might be lower than the current regulatory health limit values, especially when also considering additive or synergistic mixture effects.^13,14,88,93,94^ This suggests that many existing PVC floorings will continue to contribute to *ortho*-phthalate exposure and potential negative human-health outcomes and that recycling of such PVC floorings may lead to further prolonging these.

For alternative plasticizers, fewer exposure assessments have been conducted.^18,95−98^ Alternatives are found in similar concentrations in indoor media, albeit slightly lower than *ortho*-phthalates, and thus result in slightly lower exposures.^95−98^ Currently, health limit values for alternative plasticizers (e.g., tolerable daily intake, reference dose) are either yet to be set, or orders of magnitude higher than those for *ortho*-phthalates.^18^ As research on alternative plasticizers is still ongoing, this space has yet to be monitored.

#### 4.2.4 Health Impacts and Epidemiological Evidence

Exposure to plasticized PVC, whether from indoor PVC floorings or during production and recycling, has a strong link to plasticizer concentrations in biological tissues. Several biomarkers (e.g., urine levels of metabolites, typically in the ng/mL range) were found to correlate with exposure to PVC floorings or with occupational exposure to PVC.^43,99−101^ Several other studies point to an association of asthma and allergies with residential PVC floorings, and to the development of liver cancer in an occupational context (which is likely caused by vinyl chloride exposure, rather than additives).^102−105^

### 4.3 Implications of Our Findings on a Transition to a Safe, Circular Economy

Sustainable circular economy practices should take the chemical level into account.^35,106^ About 16% of the samples measured in this study contain legacy, regulated hazardous substances, such as DEHP and lead, at significant levels (Figure 4). Interestingly, these substances are mostly present at levels lower than typically necessary for fulfilling their functions (DEHP for plasticization: 5–65 wt %, lead for heat-stabilization: 0.05–5 wt %), suggesting their origins being ongoing uncontrolled recycling (i.e., recycling of contaminated waste materials containing these substances into new products) rather than intentional use.^9,36,107^ While DEHP had been explicitly authorized in recycled PVC materials, this practice was controversial.^16,108^ Recycling contaminated materials into long-lived products, such as floorings, prolongs exposure to and hampers an effective phase-out of hazardous chemicals. In fact, recycling can result in legacy hazardous chemicals remaining in products and materials for many decades after their initial use. For instance, the new PVC flooring sampled in this study would stay relevant for waste managers until mid-2030 or later, since floorings have a long lifetime of at least 10–15 years (this may even be increased by lifetime prolongation measures or reuse).^34^ In other words, reuse, sorting, and recycling systems decades from now will still have to deal with significant amounts of hazardous substances in end-of-life products, requiring efficient identification tools, safe disposal options, and possibly new virgin material to replace the disposed fractions.

While identifying products that contain legacy or other hazardous substances is an important tool for realizing a safe and sustainable circular economy, it remains challenging. In this study, no single product characteristics (e.g., the presence of a gray layer, which hints at recycled content; color; hardness), nor analytical technique, could serve as a simple proxy for identifying all samples of concern (Section S3.6 in the Supporting Information 1). For example, sorting out samples with a gray layer (66% of the samples) would remove only about 67% of the samples with legacy hazardous substances (“*Sensitivity*”), while losing a significant portion, 65%, of (comparatively) clean materials (“100-specificity”). Several different screening tools are compared in Section S3.6 in the Supporting Information 1 and Figure S15 in the Supporting Information 1. In this study, a combination of ATR-FTIR and XRF screening is the most effective for identifying the majority of concerning samples; however, such a combination can yet not measure many other hazardous chemicals or detect mixture effects. While bioassays may provide evidence for unknown hazardous substances and possible mixture effects, the bioassays employed in this study are very time- and resource-intensive. While YES/YAS assays identified *ortho*-phthalates well, time and resource constraints associated with sample extraction, preparation, and subsequent testing make them not suitable to realistically serve as a screening tool. Meanwhile, high-throughput screening for cytotoxicity and oxidative stress cannot be used as an indicator to replace other bioassays, such as endocrine disruption, genotoxicity, and mutagenicity. Overall, to make bioassays efficient and helpful screening tools for problematic plastics, their further development, including alternative sample preparation techniques (e.g., direct sample probing or leaching to water instead of organic solvent extraction and concentration) and higher sensitivities are needed.

### 4.4 Limitations and Uncertainties of the Present Study

Some limitations and uncertainties remain, mainly stemming from sampling, solvent selection, and the selection of analytical and data analysis parameters.

PVC flooring samples were collected from four largest DIY stores and one large retailer near Zurich, possibly leaving out supply chains for small- or medium-sized building projects. Despite our inquiries, we were not able to obtain sales or tonnage data for individual PVC flooring products; thus, their relative importance remains unclear. Product characteristics such as color, hardness, or “containing a gray layer” were manually assigned and, thus, depended on individual perception. The recycling content of products was not openly communicated, thus gray layers were used as an initial proxy, but with uncertainties as the color of recycled material may vary depending on the pretreatment (e.g., color separation) and posttreatment (e.g., coloration).^71,72^

Our study focuses on stabilizers, plasticizers, and several biological effects and covers neither all substances present in PVC floorings nor all biological effects that may be caused. To gain a complete picture, additional extraction procedures, solvents, analytical techniques, and bioassays including other cell lines and end points would be needed.^109^ For example, not all substances are soluble in THF or ACN, and mainly (semi)volatile compounds can be detected with GC–MS.^110,111^ Furthermore, some uncertainties are related to the substance identification. The low-resolution GC–MS approach employed in this study provides only approximate masses and thus leaves many uncertainties in library matching including possible misidentification. To limit the number of matches, we relied on the smaller NIST 14 library; however, with this procedure, we may have misidentified substances. High-resolution mass spectrometry, newer libraries (e.g., NIST 20 library), and other suspect lists (e.g., NORMAN Suspect List Exchange) may help overcome this issue in future research. Another issue for substance identification was the identification of UVCBs, mixtures, and chemical products with different compositions on the market (such as DiDP and DINCH). For example, differentiating individual substances from a mixture [e.g., di(2-propylheptyl) phthalate (DPHP, CASRN: 53306-54-0) from DiDP] cannot be guaranteed with the standards we employed.^112^

### 4.5 Recommendations for Future Action and Research

Hazardous chemicals in long-lived or recycled products pose a challenge to the society as a whole. Our case study on PVC flooring shows that (1) hazardous substances are present in long-lived materials, (2) uncontrolled recycling is taking place, and (3) monitoring or screening of products containing hazardous chemicals is challenging, expensive, and time-consuming. Based on our experience, we recommend the following actionable points.

Implementing current regulations, including the phase-out of hazardous chemicals, does not sufficiently cover risks associated with chemicals in long-lived or recycled products. The presence of substances in products needs to be tracked and monitored throughout their life cycle. Initiatives such as the SCIP database in the EU, and chemical audits by market surveillance bodies in different countries, are valuable steps in this this direction. However, they should ideally not only rely on self-reporting, extend to products already in use, and (for the case of databases) make a clearer link to concrete products/waste streams in the real world.^40,113^ Importantly, more stringent regulation based on the precautionary principle would be necessary to avoid burdening future recycled materials, to avoid undermining the idea and social acceptance of a circular economy, and to ensure that only clean, safe, and recyclable materials are put on the market.^114^ This may include (1) swift restriction of hazardous substances that show sufficient but not necessarily conclusive evidence for health or environmental concerns, (2) incentivizing simplification and harmonization of material options, including chemical compositions, toward standard formulations, and (3) enacting extended producer responsibility toward true recyclability.^115^

Industry action has in some cases preceded regulation of hazardous chemicals, including the early phase-out of lead and cadmium stabilizers by the EU PVC industry.^23,24^ Learning from these examples and utilizing existing industry-wide organizations (e.g., Vinyl Plus), manufacturers may pioneer and push for a swift phase-out of other hazardous substances and transition to safer and more sustainable alternatives. Furthermore, learning from the PET water bottles, manufacturers could come together, along with other actors throughout the value chains, and establish positive lists that greatly simplify and harmonize material options and chemical composition.^106^ The recycling industry may enhance sorting by employing available fast screening techniques for hazardous substances at scale (e.g., using XRF for toxic metals and bromine, ATR-FTIR for *ortho*-phthalates) and thereby avoiding at least some contamination of recycled materials. However, such efforts are expensive and may not be available to all recyclers (especially in low-income countries) and shift the burden of hazardous materials from the manufacturers to the waste management sector. Nevertheless, enhancing the traceability of chemicals throughout the life cycle of products is urgently needed, for example, by labeling. This would make it possible to proactively react to current or future findings, and make informed decisions on whether and how to recycle materials or whether disposal is the most sensible option. In practice, digital product passports that are currently being discussed could include information on the chemical composition.^116^

For consumers and designers, it is difficult to judge the safety of products based on visible characteristics; neither color, presence of layers, nor softness reliably predict the presence of hazardous substances. An independent, reliable, and easily interpretable label for building and construction products (similar to the “Blue Angel” in Germany) may simplify consumers’ decision-making. Furthermore, citizens can and should demand more transparency, appropriate regulation, and industrial responsibility for hazardous chemicals in products.^117^

Researchers should develop or improve simple, fast, and ideally comprehensive methods for identifying and removing hazardous chemicals in plastics. Ideally, this includes screening tools that can work with present and future sorting infrastructure, tolerate contamination well, and analyze the plastic directly with minimal preprocessing. Importantly, novel processes of removing hazardous chemicals will need to ensure high-quality output materials and lower environmental burden compared to incineration and other final disposal options.^118,119^ Furthermore, researchers should fill knowledge gaps regarding hazards of commonly detected emerging substances (e.g., DEHT, DINCH, tin or barium stabilizers) and their mixture toxicity in realistic exposure scenarios, taking into account everyday exposure from other sources as well.^120^

## Supporting Information Available

Methods, additional results (concentration statistics, substance correlations, screening test quality), and additional discussions (exposure to plasticizers) (PDF)
Final analysis results for each sample, further information on the analytical standards, and outputs of the different analytical techniques (XLSX)
Protocol used for *ortho*-phthalate quantitation (ZIP)
Due to size limitations of the Supporting Information in this journal, large files containing raw data (SI4–SI8) have been deposited in Mendeley Data.^121^ Available at: 10.17632/s4g2y7c7c7.2.

## References

[ref1] Schwarz A. E.; Ligthart T. N.; Godoi Bizarro D.; De Wild P.; Vreugdenhil B.; van Harmelen T. Plastic Recycling in a Circular Economy; Determining Environmental Performance through an LCA Matrix Model Approach. Waste Manag. 2021, 121, 331–342. 10.1016/j.wasman.2020.12.020. 33412464

[ref2] Plastics Europe. Plastics—the Facts 2022. https://plasticseurope.org/knowledge-hub/plastics-the-facts-2022/(accessed Sep 25, 2023).

[ref3] Wypych G. Handbook of Plasticizers, 4th ed.; Wypych G., Ed.; Elsevier, 2023. 10.1016/C2022-0-02210-8.

[ref4] ECVM—The European Council of Vinyl Manufacturers. PVC Applications. https://pvc.org/pvc-applications/.

[ref5] Weber R.; Ashta N. M.; Aurisano N.; Wang Z.; Outters M.; De Miguel K.; Schlummer M.; Blepp M.; Wiesinger H.; Andrade H.; Scheringer M.; Fantke P. Chemicals in Plastics - A Technical Report; United Nations Environment Programme, 2023. 10.59117/20.500.11822/42366.

[ref6] Wiesinger H.; Wang Z.; Hellweg S. Deep Dive into Plastic Monomers, Additives, and Processing Aids. Environ. Sci. Technol. 2021, 55, 9339–9351. 10.1021/acs.est.1c00976. 34154322

[ref7] Geueke B. Dossier - Non-intenionally added substances (NIAS) https://www.foodpackagingforum.org/food-packaging-health/non-intentionally-added-substances-nias (accessed Sep 25, 2023).

[ref8] Fischer I.; Schmitt W. F.; Porth H.-C.; Allsopp M. W.; Vianello G. Poly(Vinyl Chloride). In Ullmann’s Encyclopedia of Industrial Chemistry; Wiley-VCH Verlag GmbH & Co. KGaA: Weinheim, Germany, 2014, pp 1–30. 10.1002/14356007.a21_717.pub2.

[ref9] Wypych G. PVC Additives. PVC Formulary 2020, 47–94. 10.1016/B978-1-927885-63-5.50006-9.

[ref10] Grossman R. F. Handbook of Vinyl Formulating; Grossman R. F.; Wiley Series on Plastics Engineering and Technology, Ed.; John Wiley & Sons, Inc.: Hoboken, NJ, USA, 2008. 10.1002/9780470253595.

[ref11] Everard M. 5 - PVC and Sustainability. In PVC Additives; Schiller M., Ed.; Hanser, 2015, pp 369–410. 10.3139/9781569905449.005.

[ref12] European Plasticisers; European Chemical Industry Council (cefic). ORTHO-PHTHALATES https://www.plasticisers.org/plasticiser/ortho-phthalates/(accessed Sep 25, 2023).

[ref13] Meeker J. D.; Sathyanarayana S.; Swan S. H. Phthalates and Other Additives in Plastics: Human Exposure and Associated Health Outcomes. Philos. Trans. R. Soc. Lond. B Biol. Sci. 2009, 364 (1526), 2097–2113. 10.1098/rstb.2008.0268. 19528058 PMC2873014

[ref14] Eales J.; Bethel A.; Galloway T.; Hopkinson P.; Morrissey K.; Short R. E.; Garside R. Human Health Impacts of Exposure to Phthalate Plasticizers: An Overview of Reviews. Environ. Int. 2022, 158, 106903 10.1016/j.envint.2021.106903. 34601394

[ref15] European Chemicals Agency (ECHA). Authorisation List https://echa.europa.eu/authorisation-list (accessed Sep 25, 2023).

[ref16] European Chemicals Agency. Adopted opinion and previous consultations on applications for authorisation https://echa.europa.eu/applications-for-authorisation-previous-consultations (accessed Sep 25, 2023).

[ref122] Der Schweizerische Bundesrat. SR 814.81 - Chemikalien-Risikoreduktions-Verordnung, ChemRRV. https://www.fedlex.admin.ch/eli/cc/2005/478/de (accessed Sep 25, 2023).

[ref17] European Chemicals Agency (ECHA). Phthalates https://echa.europa.eu/hot-topics/phthalates (accessed Sep 25, 2023).

[ref18] Bui T. T.; Giovanoulis G.; Cousins A. P.; Magnér J.; Cousins I. T.; de Wit C. A. Human Exposure, Hazard and Risk of Alternative Plasticizers to Phthalate Esters. Sci. Total Environ. 2016, 541, 451–467. 10.1016/j.scitotenv.2015.09.036. 26410720

[ref19] European Chemicals Agency (ECHA). Tris(2-ethylhexyl) benzene-1,2,4-tricarboxylate (TEHTM) https://echa.europa.eu/substance-information/-/substanceinfo/100.020.019 (accessed Sep 25, 2023).

[ref20] European Chemicals Agency (ECHA); Umweltbundesamt Österreich GmbH. CoRAP - Tris(2-ethylhexyl) benzene-1,2,4-tricarboxylate (TEHTM) https://echa.europa.eu/information-on-chemicals/evaluation/community-rolling-action-plan/corap-table/-/dislist/details/0b0236e1807e4cae (accessed Sep 25, 2023).

[ref21] World Health Organization (WHO). Exposure to Lead: A Major Public Health Concern, 2nd edition https://www.who.int/publications/i/item/9789240037656 (accessed Sep 25, 2023).

[ref22] World Health Organization (WHO). Exposure to Cadmium: A Major Public Health Concern https://www.who.int/publications/i/item/WHO-CED-PHE-EPE-19-4-3 (accessed Sep 25, 2023).

[ref23] VinylPlus. The European PVC industry’s experience in replacing lead and cadmium-based stabilisers https://www.stabilisers.eu/wp-content/uploads/2015/11/VinylPlus_Contribution-Cefic_Eu-Industry.pdf (accessed Sep 25, 2023).

[ref24] European Stabilisers Producers Association (ESPA). Stabilisers – What’s new ? https://www.stabilisers.eu/wp-content/uploads/2016/01/ESPA-stabilisers_update_January-2017.pdf (accessed Sep 25, 2023).

[ref25] Fent K. Ecotoxicology of Organotin Compounds. Crit. Rev. Toxicol. 1996, 26 (1), 3–117. 10.3109/10408449609089891. 8833456

[ref26] Graceli J. B.; Sena G. C.; Lopes P. F. I.; Zamprogno G. C.; da Costa M. B.; Godoi A. F. L.; dos Santos D. M.; de Marchi M. R. R.; dos Santos Fernandez M. A. Organotins: A Review of Their Reproductive Toxicity, Biochemistry, and Environmental Fate. Reprod. Toxicol. 2013, 36, 40–52. 10.1016/j.reprotox.2012.11.008. 23228341

[ref27] Kimbrough R. D. Toxicity and Health Effects of Selected Organotin Compounds: A Review. Environ. Health Perspect. 1976, 14 (April), 51–56. 10.1289/ehp.761451. 789069 PMC1475111

[ref28] Peana M.; Medici S.; Dadar M.; Zoroddu M. A.; Pelucelli A.; Chasapis C. T.; Bjørklund G. Environmental Barium: Potential Exposure and Health-Hazards. Arch. Toxicol. 2021, 95 (8), 2605–2612. 10.1007/s00204-021-03049-5. 33870439

[ref29] Organisation for Economic Cooperation and Development (OECD). SIDS Initial Assessment Profile on zinc metal, zinc oxide, zinc distearate, zinc chloride, zinc sulphate, trizinc bis (orthophosphate) http://webnet.oecd.org/HPV/UI/handler.axd?id=9a66eb20-4489-4c7e-9711-8302cde5565b (accessed Sep 25, 2023).

[ref30] Naz M.; Ghani M. I.; Sarraf M.; Liu M.; Fan X. Ecotoxicity of Nickel and Its Possible Remediation. Phytoremediation 2022, 297–322. 10.1016/B978-0-323-89874-4.00022-4.

[ref31] Tchounwou P. B.; Yedjou C. G.; Patlolla A. K.; Sutton D. J. Heavy Metal Toxicity and the Environment BT - Molecular, Clinical and Environmental Toxicology. In Environmental Toxicology; Luch A., Ed.; Springer Basel: Basel, 2012; Vol. 3, pp 133–164. 10.1007/978-3-7643-8340-4_6. PMC4144270 22945569

[ref32] European Parliament; Council of the European Union. Council Directive 2009/48/EC on the safety of toys http://data.europa.eu/eli/dir/2009/48/2019-11-18 (accessed Sep 25, 2023).

[ref33] European Parliament; Council of the European Union. Council Directive 2011/65/EU on the restriction of the use of certain hazardous substances (RoHS) in electrical and electronic equipment (EEE) http://data.europa.eu/eli/dir/2011/65/2021-04-01 (accessed Sep 25, 2023).

[ref34] Geyer R.; Jambeck J. R.; Law K. L. Production, Use, and Fate of All Plastics Ever Made. Sci. Adv. 2017, 3 (7), 19–24. 10.1126/sciadv.1700782. PMC5517107 28776036

[ref35] Kral U.; Kellner K.; Brunner P. H. Sustainable Resource Use Requires “Clean Cycles” and Safe “Final Sinks. Sci. Total Environ. 2013, 461–462, 819–822. 10.1016/j.scitotenv.2012.08.094. PMC3749382 23017730

[ref36] Wagner S.; Schlummer M. Legacy Additives in a Circular Economy of Plastics: Current Dilemma, Policy Analysis, and Emerging Countermeasures. Resour. Conserv. Recycl. 2020, 158 (February), 104800 10.1016/j.resconrec.2020.104800.

[ref37] Klotz M.; Haupt M. A High-Resolution Dataset on the Plastic Material Flows in Switzerland. Data Brief 2022, 41, 108001 10.1016/j.dib.2022.108001. 35282173 PMC8914542

[ref38] Ciacci L.; Passarini F.; Vassura I. The European PVC Cycle: In-Use Stock and Flows. Resour. Conserv. Recycl. 2017, 123, 108–116. 10.1016/j.resconrec.2016.08.008.

[ref39] European Parliament; Council of the European Union. Council Directive 2008/98/EC on waste (Waste Framework Directive) http://data.europa.eu/eli/dir/2008/98/oj (accessed Sep 25, 2023).

[ref40] European Chemicals Agency (ECHA). SCIP Database https://echa.europa.eu/scip-database (accessed Sep 25, 2023).

[ref41] Giovanoulis G.; Bui T.; Xu F.; Papadopoulou E.; Padilla-Sanchez J. A.; Covaci A.; Haug L. S.; Cousins A. P.; Magnér J.; Cousins I. T.; de Wit C. A. Multi-Pathway Human Exposure Assessment of Phthalate Esters and DINCH. Environ. Int. 2018, 112 (April 2017), 115–126. 10.1016/j.envint.2017.12.016. 29272775

[ref42] Wang W.; Wu F. Y.; Huang M. J.; Kang Y.; Cheung K. C.; Wong M. H. Size Fraction Effect on Phthalate Esters Accumulation, Bioaccessibility and in Vitro Cytotoxicity of Indoor/Outdoor Dust, and Risk Assessment of Human Exposure. J. Hazard. Mater. 2013, 261, 753–762. 10.1016/j.jhazmat.2013.04.039. 23755845

[ref43] Carlstedt F.; Jönsson B. A. G.; Bornehag C. G. PVC Flooring Is Related to Human Uptake of Phthalates in Infants. Indoor Air 2013, 23 (1), 32–39. 10.1111/j.1600-0668.2012.00788.x. 22563949

[ref44] Xu Y.; Cohen Hubal E. A.; Little J. C. Predicting Residential Exposure to Phthalate Plasticizer Emitted from Vinyl Flooring: Sensitivity, Uncertainty, and Implications for Biomonitoring. Environ. Health Perspect. 2010, 118 (2), 253–258. 10.1289/ehp.0900559. 20123613 PMC2831926

[ref45] Little J. C.; Weschler C. J.; Nazaroff W. W.; Liu Z.; Cohen Hubal E. A. Rapid Methods to Estimate Potential Exposure to Semivolatile Organic Compounds in the Indoor Environment. Environ. Sci. Technol. 2012, 46 (20), 11171–11178. 10.1021/es301088a. 22856628

[ref46] Eichler C. M. A.; Hubal E. A. C.; Xu Y.; Cao J.; Bi C.; Weschler C. J.; Salthammer T.; Morrison G. C.; Koivisto A. J.; Zhang Y.; Mandin C.; Wei W.; Blondeau P.; Poppendieck D.; Liu X.; Delmaar C. J. E.; Fantke P.; Jolliet O.; Shin H. M.; Diamond M. L.; Shiraiwa M.; Zuend A.; Hopke P. K.; Von Goetz N.; Kulmala M.; Little J. C. Assessing Human Exposure to SVOCs in Materials, Products, and Articles: A Modular Mechanistic Framework. Environ. Sci. Technol. 2021, 55 (1), 25–43. 10.1021/acs.est.0c02329. 33319994 PMC7877794

[ref47] Kim H.-H.; Yang J.-Y.; Kim S.-D.; Yang S.-H.; Lee C.-S.; Shin D.-C.; Lim Y.-W. Health Risks Assessment in Children for Phthalate Exposure Associated with Childcare Facilities and Indoor Playgrounds. Environ. Health Toxicol. 2011, 26, e2011008 10.5620/eht.2011.26.e2011008. 22125769 PMC3214980

[ref48] Clausen P. A.; Hansen V.; Gunnarsen L.; Afshari A.; Wolkoff P. Emission of Di-2-ethylhexyl Phthalate from PVC Flooring into Air and Uptake in Dust: Emission and Sorption Experiments in FLEC and CLIMPAQ. Environ. Sci. Technol. 2004, 38 (9), 2531–2537. 10.1021/es0347944. 15180047

[ref49] Afshari A.; Gunnarsen L.; Clausen P. A.; Hansen V. Emission of Phthalates from PVC and Other Materials. Indoor Air 2004, 14 (2), 120–128. 10.1046/j.1600-0668.2003.00220.x. 15009418

[ref50] Xu Y.; Liu Z.; Park J.; Clausen P. A.; Benning J. L.; Little J. C. Measuring and Predicting the Emission Rate of Phthalate Plasticizer from Vinyl Flooring in a Specially-Designed Chamber. Environ. Sci. Technol. 2012, 46 (22), 12534–12541. 10.1021/es302319m. 23095118

[ref51] Chino S.; Kato S.; Seo J.; Ataka Y. Study on Emission of Decomposed Chemicals of Esters Contained in PVC Flooring and Adhesive. Build. Environ. 2009, 44 (7), 1337–1342. 10.1016/j.buildenv.2008.07.003.

[ref52] Bohlin-Nizzetto P. Content and migration of chemical additives from plastic products. (NILU report 9/2022). https://hdl.handle.net/11250/2992965 (accessed Sep 25, 2023).

[ref53] Liang Y.; Xu Y. Emission of Phthalates and Phthalate Alternatives from Vinyl Flooring and Crib Mattress Covers: The Influence of Temperature. Environ. Sci. Technol. 2014, 48 (24), 14228–14237. 10.1021/es504801x. 25419579

[ref54] Shi S.; Cao J.; Zhang Y.; Zhao B. Emissions of Phthalates from Indoor Flat Materials in Chinese Residences. Environ. Sci. Technol. 2018, 52 (22), 13166–13173. 10.1021/acs.est.8b03580. 30372054

[ref55] Lowe C. N.; Phillips K. A.; Favela K. A.; Yau A. Y.; Wambaugh J. F.; Sobus J. R.; Williams A. J.; Pfirrman A. J.; Isaacs K. K. Chemical Characterization of Recycled Consumer Products Using Suspect Screening Analysis. Environ. Sci. Technol. 2021, 55 (16), 11375–11387. 10.1021/acs.est.1c01907. 34347456 PMC8475772

[ref56] Kumari K.; Sharma J. K.; Kanade G. S.; Kashyap S. M.; Juwarkar A. A.; Wate S. R. Investigation of Polybrominated Diphenyl Ethers in Old Consumer Products in India. Environ. Monit. Assess. 2014, 186 (5), 3001–3009. 10.1007/s10661-013-3596-2. 24497080

[ref57] Wahl H. G.; Hoffmann A.; Häring H. U.; Liebich H. M. Identification of Plasticizers in Medical Products by a Combined Direct Thermodesorption–Cooled Injection System and Gas Chromatography–Mass Spectrometry. J. Chromatogr. A 1999, 847 (1–2), 1–7. 10.1016/S0021-9673(99)00138-7. 10515691

[ref58] Wang Q.; Storm B. K. Separation and Analysis of Low Molecular Weight Plasticizers in Poly(Vinyl Chloride) Tubes. Polym. Test. 2005, 24 (3), 290–300. 10.1016/j.polymertesting.2004.12.002.

[ref59] Yang C.; Harris S. A.; Jantunen L. M.; Kvasnicka J.; Nguyen L. V.; Diamond M. L. Phthalates: Relationships between Air, Dust, Electronic Devices, and Hands with Implications for Exposure. Environ. Sci. Technol. 2020, 54 (13), 8186–8197. 10.1021/acs.est.0c00229. 32539399

[ref60] Clausen P. A.; Liu Z.; Kofoed-Sørensen V.; Little J.; Wolkoff P. Influence of Temperature on the Emission of Di-(2-Ethylhexyl)Phthalate (DEHP) from PVC Flooring in the Emission Cell FLEC. Environ. Sci. Technol. 2012, 46 (2), 909–915. 10.1021/es2035625. 22191658

[ref61] Peeters J. R.; Vanegas P.; Kellens K.; Wang F.; Huisman J.; Dewulf W.; Duflou J. R. Forecasting Waste Compositions: A Case Study on Plastic Waste of Electronic Display Housings. Waste Manag. 2015, 46, 28–39. 10.1016/j.wasman.2015.09.019. 26431677

[ref62] Zhang L.; Wang F.; Ji Y.; Jiao J.; Zou D.; Liu L.; Shan C.; Bai Z.; Sun Z. Phthalate Esters (PAEs) in Indoor PM10/PM2.5 and Human Exposure to PAEs via Inhalation of Indoor Air in Tianjin, China. Atmos. Environ. 2014, 85, 139–146. 10.1016/j.atmosenv.2013.11.068.

[ref63] Welle F.; Wolz G.; Franz R. Migration of plasticizers from PVC tubes into enteral feeding solutions http://pieweb.plasteurope.com/members/pdf/P204322b.PDF (accessed Sep 25, 2023).

[ref64] Radaniel T.; Genay S.; Simon N.; Feutry F.; Quagliozzi F.; Barthélémy C.; Lecoeur M.; Sautou V.; Décaudin B.; Odou P.; Bernard L.; Bourdeaux D.; Chennell P.; Richard D.; Pereira B.; Azaroual N.; Christine Barthélémy Décaudin B.; Dine T.; Feutry F.; Genay S.; Kambia N.; Lecoeur M.; Odou P.; Simon N.; Vaccher C.; Cueff R.; Feschet E.; Breysse C. Quantification of Five Plasticizers Used in PVC Tubing through High Performance Liquid Chromatographic-UV Detection. J. Chromatogr., B: Anal. Technol. Biomed. Life Sci. 2014, 965, 158–163. 10.1016/j.jchromb.2014.06.027. 25023228

[ref65] Bernard L.; Cueff R.; Breysse C.; Décaudin B.; Sautou V. Migrability of PVC Plasticizers from Medical Devices into a Simulant of Infused Solutions. Int. J. Pharm. 2015, 485 (1–2), 341–347. 10.1016/j.ijpharm.2015.03.030. 25796128

[ref66] Bourdeaux D.; Yessaad M.; Chennell P.; Larbre V.; Eljezi T.; Bernard L.; Sautou V.; Azaroual N.; Barthelémy C.; Décaudin B.; Dine T.; Feutry F.; Genay S.; Kambia N. L.; Lecoeur M.; Masse M.; Odou P.; Simon N.; Vaccher C.; Daudet X.; Richard D.; Pereira B.; Clauson H.; Cueff R.; Feschet E.; Breysse C. Analysis of PVC Plasticizers in Medical Devices and Infused Solutions by GC-MS. J. Pharm. Biomed. Anal. 2016, 118, 206–213. 10.1016/j.jpba.2015.10.034. 26562183

[ref67] Faessler D.; McCombie G.; Biedermann M.; Felder F.; Subotic U. Leaching of Plasticizers from Polyvinylchloride Perfusion Lines by Different Lipid Emulsions for Premature Infants under Clinical Conditions. Int. J. Pharm. 2017, 520 (1–2), 119–125. 10.1016/j.ijpharm.2017.01.046. 28126549

[ref68] Jeon S. H.; Kim Y. P.; Kho Y.; Shin J. H.; Ji W. H.; Ahn Y. G. Development and Validation of Gas Chromatography-Triple Quadrupole Mass Spectrometric Method for Quantitative Determination of Regulated Plasticizers in Medical Infusion Sets. J. Anal. Methods Chem. 2018, 2018, 1–9. 10.1155/2018/9470254. PMC5832103 29629214

[ref69] Fernandez-Canal C.; Pinta P. G.; Eljezi T.; Larbre V.; Kauffmann S.; Camilleri L.; Cosserant B.; Bernard L.; Pereira B.; Constantin J. M.; Grimandi G.; Sautou V. Patients’ Exposure to PVC Plasticizers from ECMO Circuits. Expert Rev. Med. Devices 2018, 15 (5), 377–383. 10.1080/17434440.2018.1462698. 29658331

[ref70] Den Braver-Sewradj S. P.; Piersma A.; Hessel E. V. S. An Update on the Hazard of and Exposure to Diethyl Hexyl Phthalate (DEHP) Alternatives Used in Medical Devices. Crit. Rev. Toxicol. 2020, 50 (8), 650–672. 10.1080/10408444.2020.1816896. 33006299

[ref71] Plinke E.; Wenk N.; Gunter W.; Castiglione D.; Plamark M. Mechanical recycling of PVC wastes https://ec.europa.eu/environment/pdf/waste/studies/pvc/mech_recylce.pdf (accessed Sep 25, 2023).

[ref72] Leadbitter J. Mechanical Recycling of PVC. In Recycling of Plastics; Smith M., Ed.; Carl Hanser Verlag GmbH & Co. KG: München, 2022, pp 411–426. 10.3139/9781569908570.008.

[ref73] Löschner D.; Rapp T.; Schlosser F.-U.; Schuster R.; Stottmeister E.; Zander S. Experience with the Application of the Draft European Standard PrEN 15768 to the Identification of Leachable Organic Substances from Materials in Contact with Drinking Water by GC-MS. Anal. Methods 2011, 3 (11), 2547 10.1039/c1ay05471f.

[ref74] Christen V.; Camenzind M.; Fent K. Silica Nanoparticles Induce Endoplasmic Reticulum Stress Response, Oxidative Stress and Activate the Mitogen-Activated Protein Kinase (MAPK) Signaling Pathway. Toxicol. Rep. 2014, 1, 1143–1151. 10.1016/j.toxrep.2014.10.023. 28962324 PMC5598250

[ref75] European Commission; European Parliament. Comission regulation (EU 2021/2045) amending REACH (EC No 1907/2006) http://data.europa.eu/eli/reg/2021/2045/oj (accessed Sep 25, 2023).

[ref76] European Parliament; Council of the European Union. Council Directive 2005/84/EC on phthalates in toys and childcare articles https://eur-lex.europa.eu/eli/dir/2005/84/oj (accessed Sep 25, 2023).

[ref77] United States Consumer Product Safety Comission (US CPSC). Phthalates and Phthalate Substitutes in Children’s Toys https://www.cpsc.gov/s3fs-public/phthallab.pdf (accessed Sep 25, 2023).

[ref78] Rastogi S. C. Gas Chromatographic Analysis of Phthalate Esters in Plastic Toys. Chromatographia 1998, 47 (11–12), 724–726. 10.1007/BF02467461.

[ref79] McCombie G.; Biedermann S.; Suter G.; Biedermann M. Survey on Plasticizers Currently Found in PVC Toys on the Swiss Market: Banned Phthalates Are Only a Minor Concern. J. Environ. Sci. Health, Part A: Toxic/Hazard. Subst. Environ. Eng. 2017, 52 (5), 491–496. 10.1080/10934529.2016.1274176. 28129041

[ref80] Ashworth M.; Chappell A.; Ashmore E.; Fowles J. Analysis and Assessment of Exposure to Selected Phthalates Found in Children’s Toys in Christchurch, New Zealand. Int. J. Environ. Res. Public Health 2018, 15 (2), 200 10.3390/ijerph15020200. 29370098 PMC5858269

[ref81] Al-Natsheh M.; Alawi M.; Fayyad M.; Tarawneh I. Simultaneous GC-MS Determination of Eight Phthalates in Total and Migrated Portions of Plasticized Polymeric Toys and Childcare Articles. J. Chromatogr., B: Anal. Technol. Biomed. Life Sci. 2015, 985, 103–109. 10.1016/j.jchromb.2015.01.010. 25667041

[ref82] European Chemicals Agency (ECHA). Harmonised enforcement project on restrictions https://echa.europa.eu/documents/10162/17088/ref_4_report_en.pdf/b53f5cd9-64a4-c120-1953-e9e176b9c282 (accessed Sep 25, 2023).

[ref83] Ismail S. N. S.; Mohamad N. S.; Karuppiah K.; Abidin E. Z.; Rasdi I.; Praveena S. M. Heavy metals content in low-priced toys http://www.arpnjournals.org/jeas/research_papers/rp_2017/jeas_0317_5787.pdf (accessed Sep 25, 2023).

[ref84] Kumar A.; Pastore P. Lead and cadmium in soft plastic toys http://www.jstor.org/stable/24099126 (accessed Sep 25, 2023).

[ref85] Turner A.; Filella M. Polyvinyl Chloride in Consumer and Environmental Plastics, with a Particular Focus on Metal-Based Additives. Environ. Sci.: Processes Impacts 2021, 23 (9), 1376–1384. 10.1039/D1EM00213A. 34368828

[ref86] Meng J.; Xu B.; Liu F.; Li W.; Sy N.; Zhou X.; Yan B. Effects of Chemical and Natural Ageing on the Release of Potentially Toxic Metal Additives in Commercial PVC Microplastics. Chemosphere 2021, 283 (October 2020), 131274 10.1016/j.chemosphere.2021.131274. 34182647

[ref87] Oyeyiola A. O.; Akinyemi M. I.; Chiedu I. E.; Fatunsin O. T.; Olayinka K. O. Statistical Analyses and Risk Assessment of Potentially Toxic Metals (PTMS) in Children’s Toys. J. Taibah Univ. Sci. 2017, 11 (6), 842–849. 10.1016/j.jtusci.2017.02.005.

[ref88] Vogel N.; Schmidt P.; Lange R.; Gerofke A.; Sakhi A. K.; Haug L. S.; Jensen T. K.; Frederiksen H.; Szigeti T.; Csákó Z.; Murinova L. P.; Sidlovska M.; Janasik B.; Wasowicz W.; Tratnik J. S.; Mazej D.; Gabriel C.; Karakitsios S.; Barbone F.; Rosolen V.; Rambaud L.; Riou M.; Murawski A.; Leseman D.; Koppen G.; Covaci A.; Lignell S.; Lindroos A. K.; Zvonar M.; Andryskova L.; Fabelova L.; Richterova D.; Horvat M.; Kosjek T.; Sarigiannis D.; Maroulis M.; Pedraza-Diaz S.; Cañas A.; Verheyen V. J.; Bastiaensen M.; Gilles L.; Schoeters G.; Esteban-López M.; Castaño A.; Govarts E.; Koch H. M.; Kolossa-Gehring M. Current Exposure to Phthalates and DINCH in European Children and Adolescents – Results from the HBM4 EU Aligned Studies 2014 to 2021. Int. J. Hyg Environ. Health 2023, 249, 114101 10.1016/j.ijheh.2022.114101. 36805185

[ref89] Qadeer A.; Kirsten K. L.; Ajmal Z.; Xingru Z. Rebuttal to Comment on “Alternative Plasticizers As Emerging Global Environmental and Health Threat: Another Regrettable Substitution?” - Focus on DINCH as an Example. Environ. Sci. Technol. 2022, 56 (8), 5294–5297. 10.1021/acs.est.2c01849. 35377621

[ref90] U.S. EPA (Environmental Protection Agency). Framework for Metals Risk Assessment https://www.epa.gov/risk/framework-metals-risk-assessment (accessed Sep 25, 2023).

[ref91] Njati S. Y.; Maguta M. M. Lead-Based Paints and Children’s PVC Toys Are Potential Sources of Domestic Lead Poisoning – A Review. Environ. Pollut. 2019, 249, 1091–1105. 10.1016/j.envpol.2019.03.062. 31146315

[ref92] Boyle D.; Catarino A. I.; Clark N. J.; Henry T. B. Polyvinyl Chloride (PVC) Plastic Fragments Release Pb Additives That Are Bioavailable in Zebrafish. Environ. Pollut. 2020, 263, 114422 10.1016/j.envpol.2020.114422. 32244159

[ref93] Caporale N.; Leemans M.; Birgersson L.; Germain P. L.; Cheroni C.; Borbély G.; Engdahl E.; Lindh C.; Bressan R. B.; Cavallo F.; Chorev N. E.; D’Agostino G. A.; Pollard S. M.; Rigoli M. T.; Tenderini E.; Tobon A. L.; Trattaro S.; Troglio F.; Zanella M.; Bergman Å.; Damdimopoulou P.; Jönsson M.; Kiess W.; Kitraki E.; Kiviranta H.; Nånberg E.; Öberg M.; Rantakokko P.; Rudén C.; Söder O.; Bornehag C. G.; Demeneix B.; Fini J. B.; Gennings C.; Rüegg J.; Sturve J.; Testa G. From Cohorts to Molecules: Adverse Impacts of Endocrine Disrupting Mixtures. Science 2022, 375 (6582), eabe8244 10.1126/science.abe8244. 35175820

[ref94] HBM4 EU. Phthalates and Hexamoll® DINCH https://www.hbm4eu.eu/hbm4eu-substances/phthalates-and-hexamoll-dinch/(accessed Sep 25, 2023).

[ref95] Rudel R. A.; Camann D. E.; Spengler J. D.; Korn L. R.; Brody J. G. Phthalates, Alkylphenols, Pesticides, Polybrominated Diphenyl Ethers, and Other Endocrine-Disrupting Compounds in Indoor Air and Dust. Environ. Sci. Technol. 2003, 37 (20), 4543–4553. 10.1021/es0264596. 14594359

[ref96] Fromme H.; Schütze A.; Lahrz T.; Kraft M.; Fembacher L.; Siewering S.; Burkardt R.; Dietrich S.; Koch H. M.; Völkel W. Non-Phthalate Plasticizers in German Daycare Centers and Human Biomonitoring of DINCH Metabolites in Children Attending the Centers (LUPE 3). Int. J. Hyg Environ. Health 2016, 219 (1), 33–39. 10.1016/j.ijheh.2015.08.002. 26338253

[ref97] Nagorka R.; Conrad A.; Scheller C.; Süßenbach B.; Moriske H. J. Diisononyl 1,2-Cyclohexanedicarboxylic Acid (DINCH) and Di(2-Ethylhexyl) Terephthalate (DEHT) in Indoor Dust Samples: Concentration and Analytical Problems. Int. J. Hyg Environ. Health 2011, 214 (1), 26–35. 10.1016/j.ijheh.2010.08.005. 20851676

[ref98] Larsson K.; Lindh C. H.; Jönsson B. A.; Giovanoulis G.; Bibi M.; Bottai M.; Bergström A.; Berglund M. Phthalates, Non-Phthalate Plasticizers and Bisphenols in Swedish Preschool Dust in Relation to Children’s Exposure. Environ. Int. 2017, 102, 114–124. 10.1016/j.envint.2017.02.006. 28274486

[ref99] Fréry N.; Santonen T.; Porras S. P.; Fucic A.; Leso V.; Bousoumah R.; Duca R. C.; El Yamani M.; Kolossa-Gehring M.; Ndaw S.; Viegas S.; Iavicoli I. Biomonitoring of Occupational Exposure to Phthalates: A Systematic Review. Int. J. Hyg Environ. Health 2020, 229 (January), 113548 10.1016/j.ijheh.2020.113548. 32659708

[ref100] Just A. C.; Miller R. L.; Perzanowski M. S.; Rundle A. G.; Chen Q.; Jung K. H.; Hoepner L.; Camann D. E.; Calafat A. M.; Perera F. P.; Whyatt R. M. Vinyl Flooring in the Home Is Associated with Children’s Airborne Butylbenzyl Phthalate and Urinary Metabolite Concentrations. J. Expo. Sci. Environ. Epidemiol. 2015, 25 (6), 574–579. 10.1038/jes.2015.4. 25690585 PMC4540696

[ref101] Shu H.; Jönsson B. A. G.; Gennings C.; Lindh C. H.; Nånberg E.; Bornehag C. G. PVC Flooring at Home and Uptake of Phthalates in Pregnant Women. Indoor Air 2019, 29 (1), 43–54. 10.1111/ina.12508. 30240038

[ref102] Larsson M.; Hägerhed-Engman L.; Kolarik B.; James P.; Lundin F.; Janson S.; Sundell J.; Bornehag C. G. PVC - as Flooring Material - and Its Association with Incident Asthma in a Swedish Child Cohort Study. Indoor Air 2010, 20 (6), 494–501. 10.1111/j.1600-0668.2010.00671.x. 21070375

[ref103] Tuomainen A.; Seuri M.; Sieppi A. Indoor Air Quality and Health Problems Associated with Damp Floor Coverings. Int. Arch. Occup. Environ. Health 2004, 77 (3), 222–226. 10.1007/s00420-003-0481-2. 14689309

[ref104] Bornehag C. G.; Nanberg E. Phthalate Exposure and Asthma in Children. Int. J. Androl. 2010, 33 (2), 333–345. 10.1111/j.1365-2605.2009.01023.x. 20059582

[ref105] Choi J.; Chun C.; Sun Y.; Choi Y.; Kwon S.; Bornehag C. G.; Sundell J. Associations between Building Characteristics and Children’s Allergic Symptoms - A Cross-Sectional Study on Child’s Health and Home in Seoul, South Korea. Build. Environ. 2014, 75, 176–181. 10.1016/j.buildenv.2014.01.019.

[ref106] Wang Z.; Praetorius A. Integrating a Chemicals Perspective into the Global Plastic Treaty. Environ. Sci. Technol. Lett. 2022, 9 (12), 1000–1006. 10.1021/acs.estlett.2c00763. 36530847 PMC9753957

[ref107] Guzzonato A.; Puype F.; Harrad S. J. Evidence of Bad Recycling Practices: BFRs in Children’s Toys and Food-Contact Articles. Environ. Sci.: Processes Impacts 2017, 19 (7), 956–963. 10.1039/C7EM00160F. 28636053

[ref108] ClientEarth. EU Commission illegally allowed use of toxic chemical DEHP in recycled plastic – Advocate General https://www.clientearth.org/latest/press-office/press/eu-commission-illegally-allowed-use-of-toxic-chemical-dehp-in-recycled-plastic-advocate-general/(accessed Sep 25, 2023).

[ref109] Severin I.; Souton E.; Dahbi L.; Chagnon M. C. Use of Bioassays to Assess Hazard of Food Contact Material Extracts: State of the Art. Food Chem. Toxicol. 2017, 105, 429–447. 10.1016/j.fct.2017.04.046. 28476634

[ref110] Möller J.; Strömberg E.; Karlsson S. Comparison of Extraction Methods for Sampling of Low Molecular Compounds in Polymers Degraded during Recycling. Eur. Polym. J. 2008, 44 (6), 1583–1593. 10.1016/j.eurpolymj.2008.03.027.

[ref111] Deutsch J. C. [2] Gas chromatographic/mass spectrometric measurement of ascorbic acid and analysis of ascorbic acid degradation in solution. Methods Enzymol. 1997, 279 (1945), 13–24. 10.1016/S0076-6879(97)79004-9. 9211252

[ref112] Gries W.; Ellrich D.; Küpper K.; Ladermann B.; Leng G. Analytical Method for the Sensitive Determination of Major Di-(2-Propylheptyl)-Phthalate Metabolites in Human Urine. J. Chromatogr., B: Anal. Technol. Biomed. Life Sci. 2012, 908, 128–136. 10.1016/j.jchromb.2012.09.019. 23040987

[ref113] United Nations Environment Programme (UNEP); Strategic Approach to International Chemicals Management (SAICM); Inter-Organization Programme for the sound management of chemicals (IOMC). The Chemicals in Products (CiP) Programme https://www.unep.org/resources/other-evaluation-reportsdocuments/chemicals-products-cip-programme (accessed Sep 25, 2023).

[ref114] Bourguignon D.; The Precautionary Principle: Definitions, Applications and Governance - in-Depth Analysis. Eur. Parliam. Res. Serv. 2016, 10.2861/821468.

[ref115] Klotz M.; Haupt M.; Hellweg S. Limited Utilization Options for Secondary Plastics May Restrict Their Circularity. Waste Manag. 2022, 141 (February), 251–270. 10.1016/j.wasman.2022.01.002. 35158311

[ref116] European Commission; European Health and Digital Executive Agency (HaDEA). Digital Product Passport https://hadea.ec.europa.eu/calls-proposals/digital-product-passport_en (accessed Sep 25, 2023).

[ref117] Blue Angel-The German Ecolabel; German Environmental Agency. Environmentally Friendly Floor Coverings, Panels, Doors (DE-UZ 176) https://www.blauer-engel.de/en/productworld/floor-coverings-panels-doors-made-of-wood (accessed Sep 25, 2023).

[ref118] Freegard K.; Tan G.; Morton R.; wrap. Develop a process to separate brominated flame retardants from WEEE polymers https://polymerandfire.files.wordpress.com/2015/04/brominatedwithappendices-3712.pdf (accessed Sep 25, 2023).

[ref119] Klotz M.; Oberschelp C.; Salah C.; Subal L.; Hellweg S. The Role of Chemical and Solvent-Based Recycling within a Sustainable Circular Economy for Plastics. Sci. Total Environ. 2024, 906, 167586 10.1016/j.scitotenv.2023.167586. 37804985

[ref120] Escher B. I.; Stapleton H. M.; Schymanski E. L. Tracking Complex Mixtures of Chemicals in Our Changing Environment. Science 2020, 367 (6476), 388–392. 10.1126/science.aay6636. 31974244 PMC7153918

[ref121] Wiesinger H. Legacy and Emerging Plasticizers and Stabilizers in PVC Floorings - Rawdata. Mendeley Data 2023, 10.17632/s4g2y7c7c7.1. PMC10832040 38241221

