# Supplementary material for: Legacy and Emerging Plasticizers and Stabilizers in PVC Floorings and Implications for Recycling
Source: Environ Sci Technol. 2024 Jan 19;58(4):1894–907. doi: 10.1021/acs.est.3c04851 (PMC10832040; doi:10.1021/acs.est.3c04851)
Supplement: Supplementary file 1 — es3c04851_si_001.pdf [file es3c04851_si_001.pdf]

Supporting Information File 1 (SI1)

Legacy and emerging plasticizers and stabilizers in PVC floorings and implications for recycling

Environmental, Science & Technology

https://doi.org/10.1021/acs.est.3c04851

Helene Wiesinger[1*], Christophe Bleuler[2], Verena Christen[3], Philippe Favreau[2], Stefanie Hellweg[1,4], Miriam Langer[3,5], Roxane Pasquettaz[2], Andreas Schönborn[6], Zhanyun Wang[1,4,7*]

[1] Chair of Ecological Systems Design, Institute of Environmental Engineering, ETH Zürich, 8093 Zürich, Switzerland

[2] Service de l'air, du bruit et des rayonnements non ionisants (SABRA), Geneva cantonal office for the environment, 1205 Geneva, Switzerland

[3] Institute for Ecopreneurship, School of Life Sciences, University of Applied Sciences and Arts Northwestern Switzerland, FHNW, 4132 Muttenz, Switzerland

[4] National Centre of Competence in Research (NCCR) Catalysis, Institute of Environmental Engineering, ETH Zürich, 8093 Zürich, Switzerland

[5] Eawag – Swiss Federal Institute of Aquatic Science and Technology, 8600 Dübendorf

[6] Institute of Natural Resource Sciences, ZHAW Zurich University of Applied Science, 8820 Wädenswil, Switzerland

[7] Empa – Swiss Federal Laboratories for Materials Science and Technology, 9014 St. Gallen, Switzerland

* Contact information: Helene Wiesinger: wiesinger@ifu.baug.ethz.ch; Zhanyun Wang: zhanyun.wang@ifu.baug.ethz.ch

TABLE OF CONTENTS

S1 Background S1
S2 Metods S3
S2.1 Samples S3
S2.2 Materials S6
S2.3 Chemical analysis S10
S2.3.1 ATR-FTIR S11
S2.3.2 XRF elemental composition S12
S2.3.3 GC-MS quantification of phthalates S13
S2.3.4 GC-MS suspect screening S19
S2.4 Details Bioassays S32
S2.5 Data treatment S35
S3 Results S36
S3.1 Concentrations and presence of individual substances S36
S3.2 Total plasticizer content S41
S3.3 Correlation between Substances S42
S3.4 Bioassay results S43
S3.5 Linear regression models S46
S3.5.1 Toxic metals – presence and concentration S46
S3.5.2 *ortho*-Phthalates – presence and concentration S47
S3.5.3 Alternative plasticizers – presence S49
S3.5.4 Bioassays S49
S3.6 Screening quality metrics S50
S4 Discussion S54
S4.1 Chemical substances in PVC flooring S54
S4.2 Chemical substances in other PVC products S54
S4.3 Exposure to *ortho*-Phthalates and alternative plasticizers S56
References S60

TABLE OF TABLES

Table S 1: Legal and industrial developments in the European Union relevant for *ortho*-phthalates and metal(loids) in plastic products. S1

Table S 2 Pictures of samples used for the GC-MS analysis S3

Table S 3: Overview over certified reference materials (CRM) used in this study. S7

Table S 4: Overview over employed standards and in which workflow they were used. S8

Table S 5: ATR-FTIR settings used to determine the polymer type and the presence of *ortho*-phthalates. S11

Table S 6: Quality of ATR-FTIR screening for *ortho*-phthalates, using confusion matrices, sensitivity and specificity S11

Table S 7 Limits of detection (LODs) for the Niton XL3 GOLDD XRF for PVC matrices for a 30-second analysis time per filter, calculated from our measurements and under ideal conditions according to the instruments plastic calibration. S12

Table S 8: *ortho*-Phthaltate standards used in the *ortho*-phthalate quantification workflow. S16

Table S 9: Selection of most suitable value based on detection situation. S22

Table S 10: Analytical standards (including *ortho*-phthalates, alternative plasticizer, phosphate plasticizers, brominated flame retardants, antioxidants and bisphenols) used in the suspect-screening workflow. Overview of massspectra and approximate calibration curves for GC-MS suspect-screening.. .... S24

Table S11: Selected extracts for further screening with YES/YAS, umuC and AMES bioassays based on MTT viability and *ortho*-phthalate content. S32

Table S12: Concentration and presence of individual elements based on XRF elemental analysis. S36

Table S 13: Concentration and presence of individual *ortho*-phthalates based on the phthalate GC-MS quantification workflow... .... S37

Table S14: Concentration and presence of individual substances based on GC-MS suspect screening workflow. S38

Table S 15: Linear regression model for predicting the chance of any toxic metal(loids) being present (in %) based on sample properties (independent variables). S46

Table S 16: Linear regression model for predicting the concentration in ppm of toxic metal(loids), i.e. Cd, Pb, Cr, Ni, Hg, As, based on sample properties (independent variables) S46

Table S 17: Linear regression model for predicting the chance of any of *ortho*-phthalates being present (in %) based on sample properties (independent variables). S47

Table S 18: Linear regression model for predicting the concentration (in wt%) of *ortho*-phthalates based on sample properties (independent variables). S47

Table S 19: Linear regression model for predicting the chance of regulated *ortho*-phthalates being present (in %) based on sample properties (independent variables). S48

Table S 20: Linear regression model for predicting the concentration (in wt%) of regulated *ortho*-phthalates based on sample properties (independent variables). S48

Table S 21 Linear regression model for predicting the chance of alternative plasticizers being present (in %) based on sample properties (independent variables). S49

Table S 22: Linear regression model for predicting the chance of activity in any of the bioassay (in %) based on sample properties (independent variables). S49

Table S 23: Quality of different screening methods for determining samples of clear concern and those of any concern (possible + clear concern) using confusion matrices. S50

Table S 24 Recent studies investigating plasticizers and other substances present in PVC flooring. The country was not specified for all studies, the location of the main authors are given in parenthesis if no details were mentioned. S54

Table S 25: Recent studies investigating stabilizers and other metals present in PVC products. S54

Table S 26: Recent studies investigating plasticizers and other organic substances present in other PVC products (not flooring). S55

Table S 27: Measured and modelled indoor media concentrations of different plasticizers. S58

Table S 28 Estimated exposure to different plasticizers. S59

TABLE OF FIGURES

Figure S 1: Chemical space of the substances in the suspect list S6

Figure S 2: Sample preparation and overview for GC-MS *ortho*-phthalate quantifcaiton, GC-MS suspect screening and testing of biological activities. S10

Figure S 3: Chromatogram of all *ortho*-phthalate standards at ~5 µg/mL (DiNP and DiDP at ~50 µg/mL) using the *ortho*-phthalate quantification workflow. S14

Figure S 4: Chromatogram of all *ortho*-phthalate standards (PHT solution) using the suspect screening workflow S20

Figure S 5: Chromatogram of alternative plasticizer standards (Add solution) using the suspect screening workflow S21

Figure S 6: Peak area vs concentration for different types of standards used in the suspect screening workflow. S23

Figure S 7: Chemical space of the substances detected in suspect list screening, marker size scaled to their total chromatogram area. S39

Figure S 8: Chemical space of the substances detected in suspect list screening, marker size scaled to their detection frequency. S40

Figure S9: Plasticizer composition and amount by sample. S41

Figure S 10: Correlation matrix for detection and logarithmic concentration of all measured samples S42

Figure S 11: Cell viability and induction of oxidative stress in Huh7 cells after exposure to plastic extracts S43

Figure S 12: Endocrine activity of selected plastic extracts. S44

Figure S 13: Induction of mutagenicity by extract d21.1. .... S44

Figure S 14 Genotoxicity of selected samples, measured with the planar-umuC bioassay S45

Figure S 15: Utility of different screening methods.. .... S53

S1 BACKGROUND

Table S 1: Legal and industrial developments in the European Union relevant for *ortho*-phthalates and metal(loids) in plastic products.

Chemical	Development	Year	Description	PVC flooring relevance	source
***ortho*-phthalates**	SVHC Candidate list and SCIP database	several	Several phthalates are on the candidate list and are substances of very high concern (SVHCs), products containing more than 0.1 weight% of these must be notified to the SCIP database.<br>2008-2010 DiBP, DBP, BBP, DEHP<br>2011 DMEP, Diisoheptyl and Diisooctyl pht<br>2012-2013 DPP, DiPP, nPiPP, DHP, Diisohexyl pht<br>2018 DCHP	x	[1,2]
	Authorisation list	several	Several phthalates require authorization in the EU market with the following sunset dates<br>2015 DiBP, DBP, BBP, DEHP<br>2020 DiPP, DMEP, DPP, nPiPP, Diisoheptyl and Diiso pht<br>2023 DHP, Diisohexyl and Diisooctyl pht	x	[3]
	Restriction list	several	Several phthalates are restricted in several products<br>2016 DiBP, DBP, BBP, DEHP, DNOP DiNP, DiDP, Diisooctyl pht.	x	[4]
	ECHA opinion	Future	ECHA has assessed similar substances (*ortho*-phthalates, isophthalates, terephthalates; and, trimellitates.) as groups and found that:<br>…the use of many *ortho*-phthalates may need to be limited in the future. Some will need harmonised classification and labelling or identification as substances of very high concern (SVHC). But there are also phthalates for which more data is needed to confirm the potential hazard, and for a few no regulatory actions are needed for the time being.	x	[5]
	Toys directive	2009	Bans any carcinogens, mutagens or reprotoxicants (CMRs) in toys:<br>CMRs DiBP, DBP, BBP, DEHP, DiNP, DiDP,…<br>Earlier some were regulated by the phthalates in toys and childcare articles directive:<br>earlier dir. DBP, BBP, DEHP, DiNP, DiDP		[6,7]
	RoHS directive	2015	This directive restricts the use of several hazardous substances in the manufacture and recycling of various types of electronic and electrical equipment<br>2015 DiBP, DBP, BBP, DEHP		[8]

https://doi.org/10.1021/acs.est.3c04851

**Metal(loids)**	Cadmium and lead phase	several	The PVC industry has voluntarily phased-out cadmium and lead stabilizers<br>2001 — cadmium<br>2015 — lead	x	[9–11]
	Toys directive	2009	Sets thresholds for several metal(loids) in hard toys:<br><0.1 weight% — antimony, arsenic, cadmium, chromium, cobalt, lead, mercury, nickel, selenium, organic tin<br>>0.1 weight% — aluminium, barium, boron, copper, manganese, strontium, tin, zinc		[6]
	RoHS directive	2006	This directive restricts the use of several hazardous substances in the manufacture and recycling of various types of electronic and electrical equipment. Substances have to be below a threshold of 0.1 weight% (exception Cadmium: 0.01 weight%)<br>2006 — lead, mercury, cadmium, hexavalent chromium		[8]

S2 METODS

S2.1 Samples

An overview for all samples and the analysis results for each sample are provided on Sheet S1 in the supplementary information File 2 (SI2).

Table S 2 Pictures of samples used for the GC-MS analysis

Samples	Picture	Samples	Picture
**Batch 1**<br>d20-1 g1<br>g2 g3<br>g5 g6<br>ga1	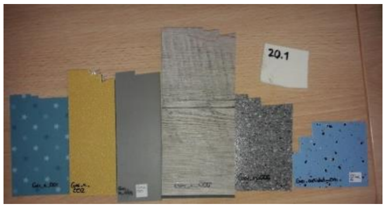	**Batch 2**<br>d21-1 d21-2<br>d30-2 d52-2<br>d54-2	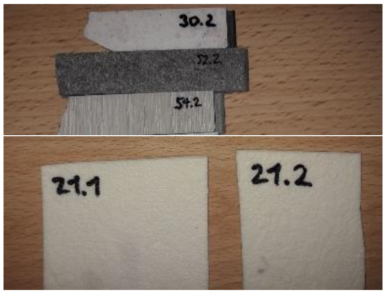
**Batch 3**<br>d1-1 d1-2<br>d2-1 d2-2<br>d4-1 d4-2<br>d5-1 d5-2<br>d20-2 d80-1	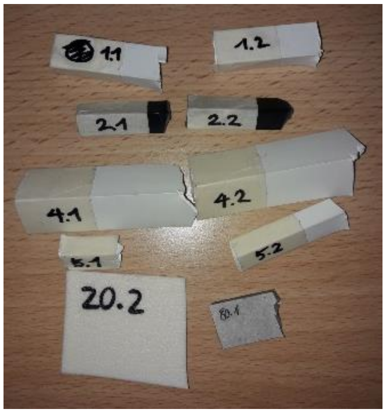	**Batch 4**<br>d6-1 d6-2<br>d7-1 d7-2<br>g4 g7<br>ga2 gar1<br>gb1	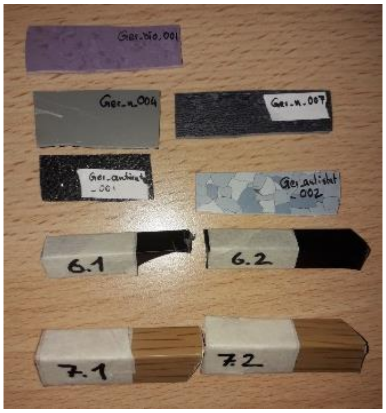
**Batch 5**<br>d8-1 d8-2<br>d9-1 d9-2<br>d10-1 d10-2<br>d11-1 d11-2<br>d12-1 d12-2<br>d13-1 d13-2	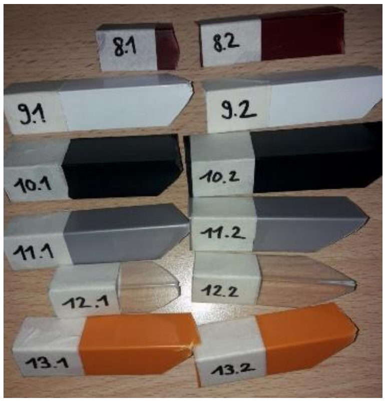	**Batch 6**<br>d42-1 d42-2<br>d43-1 d43-2<br>d44-1 d44-2<br>d71-1 d71-2<br>d73-1 d73-2<br>d74-1 d74-2<br>d75-1 d75-2<br>d76-1 d76-2<br>d77-1 d77-2	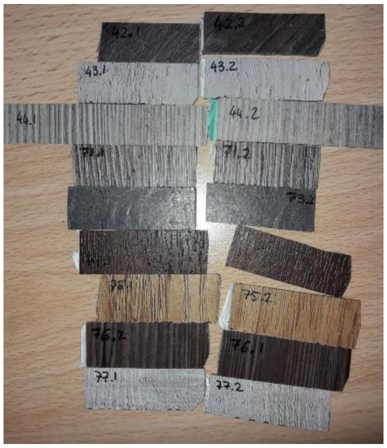

Samples	Picture	Samples	Picture
**Batch 7**<br>d30-1<br>d31-1 d31-2<br>d32-1 d32-2<br>d33-1 d33-2<br>d34-1 d34-2<br>d35-1 d35-2<br>d36-1 d36-2<br>d37-1 d37-2<br>d38-1 d38-2<br>d39-1 d39-2	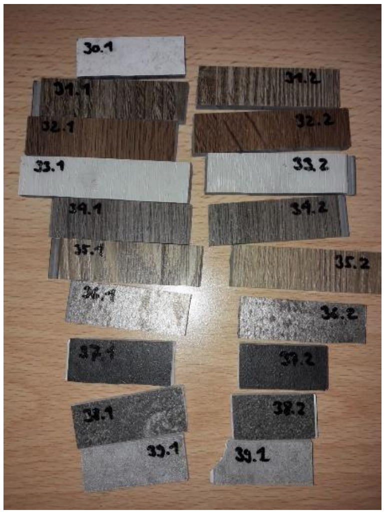	**Batch 8**<br>d27-1 d27-2<br>d28-1 d28-2<br>d29-1 d29-2	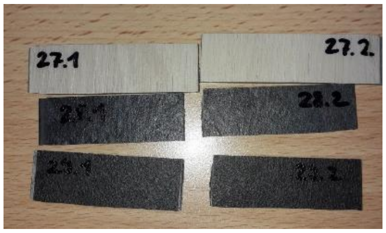
**Batch 9**<br>d40-1 d40-2<br>d41-1 d41-2<br>d45-1 d45-2<br>d46-1 d46-2<br>d48-1 d48-2<br>d52-1 d53-1<br>d53-2 d54-1<br>d55-1 d55-2<br>d59-1 d59-2	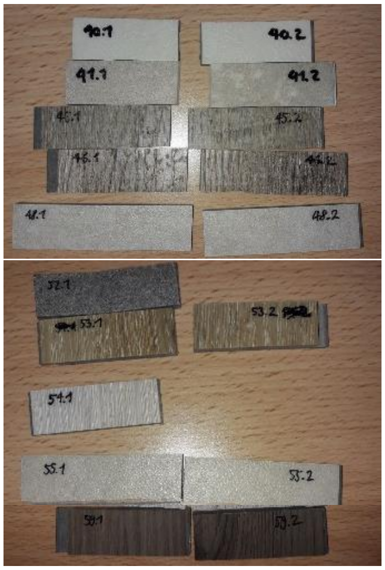	**Batch 10**<br>d60-1 d60-2<br>d61-1 d61-2<br>d62-1 d62-2<br>d63-1 d63-2	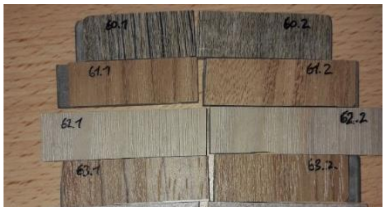
**Batch 11**<br>d03-1 d03-2<br>d50-1 d50-2<br>d51-1 d51-2<br>d72-1 d72-2<br>d80-2	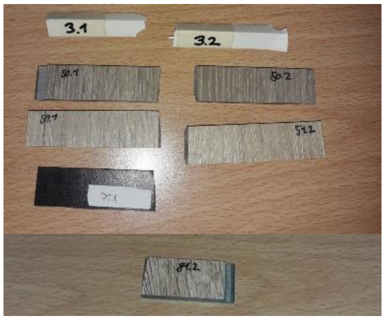	**Batch 12**<br>g08 g09<br>g10 g11<br>g12 g13<br>g14 g15<br>g16 g17	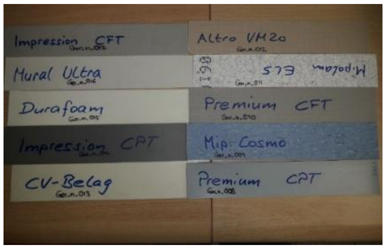
**Batch 13**<br>g18 g19<br>g20 g21<br>g22 g23<br>g24 g25<br>g26 g27	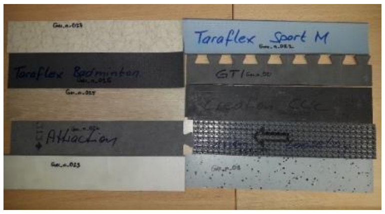	**Batch 14**<br>g28 g29<br>g30 g31<br>g32 g33<br>g34 g35<br>g36 g37	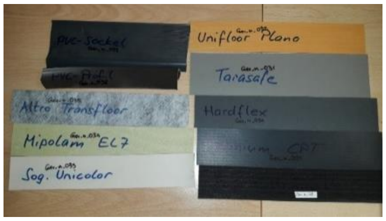

Sample characteristics. Color of the top layer or decorative sheet, hardness, number of layers, presence of a grey layer, and presence of a glass-fiber layer were assigned to each sample (see Sheet S1 in SI2), based on one author’s perception.

- Color of the top layer sometimes contained patterns or multiple colors (see Table S 2 for pictures) and was simplified to fit these categories based on the “main“ color: *black, grey, wood, orange/beige/brown, red, blue/green, white/transparent*
- Hardness was determined by bending the samples: *hard* – sample cannot be bent by hand, *medium* – sample can be bent, but with significant resistance, *soft* – sample can be easily bent, with little to no resistance
- Number of layers was determined by the number of different colored layers that can be seen without further magnification.
- Presence of a grey layer was determined based on all layers (including the top layer / coloring) and was used a proxy for recycled material (based on personal communication with a large PVC flooring retailer)
- Presence of a glass-fiber layer was determined based on a “cracking” sound, when bending the sample (based on personal communication with a large PVC flooring retailer)

S2.2 Materials

All chemical standards that were used in this study can be found in Table S 4, their position on the chemical space plot (logKow – logKaw) can be found in Figure S 1. Further information on the substances, including other identifiers, physical-chemical properties and experimental properties are provided in the Sheet S2 – Substances in SI2.

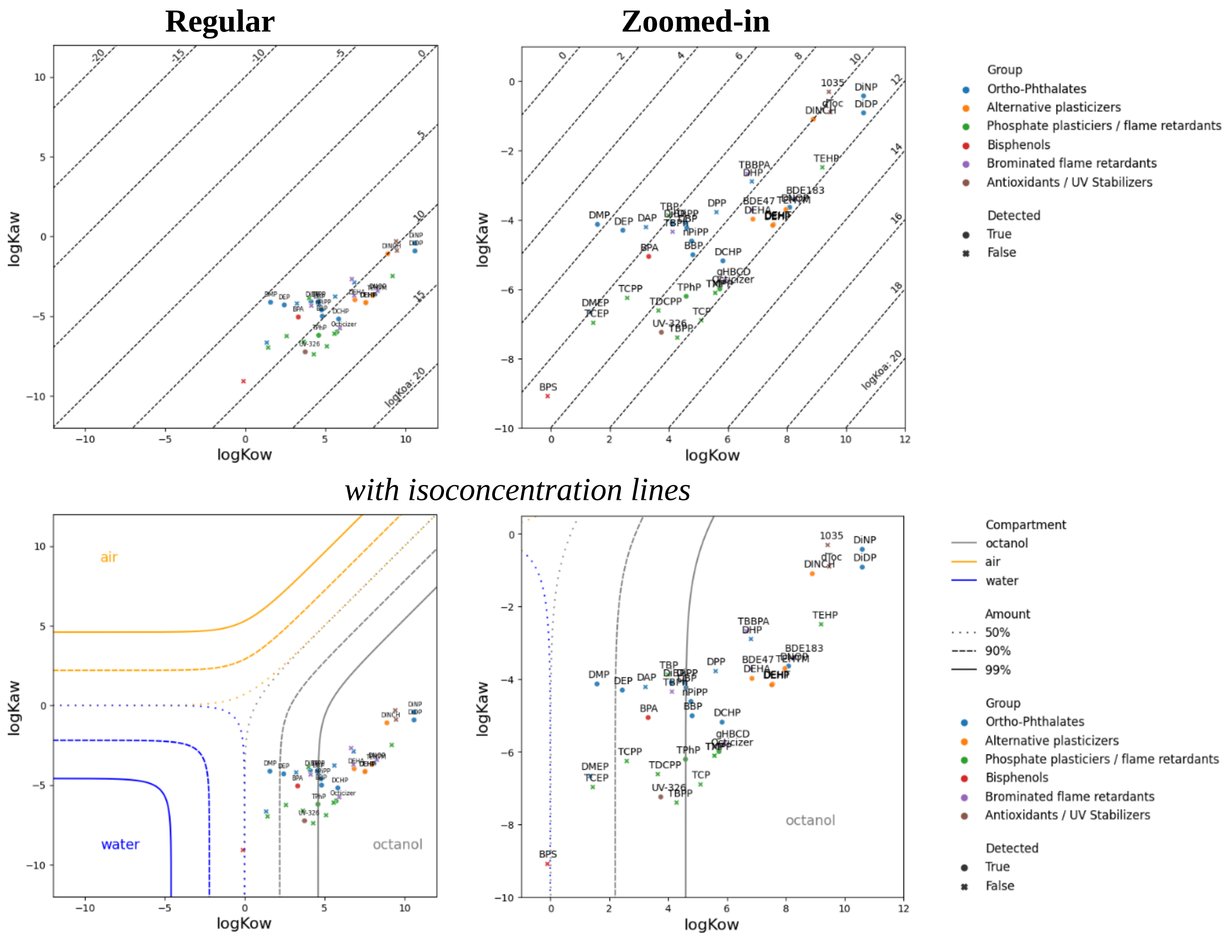

Figure S 1: Chemical space of the substances in the suspect list. For the bottom plots, the iso-concentration curves are calculated for equal volumes of each compartment (i.e. water, air and octanol are exactly the same volume). For the bottom plot the Most suspects have a very high Kow and a low Kaw, meaning they are mainly found in octanol-like environments, this is especially striking for DEHP, DiNP, DiDP and the alternative plasticizers. Phthalates are more likely to vaporize than phosphate based plasticizers due to their higher Kaw.

 https://doi.org/10.1021/acs.est.3c04851

Information on the employed reference materials for the chemical analyses and materials for the bioassays can be found in Table S 3.

Table S 3: Overview over certified reference materials (CRM) used in this study.

<table>
<tr><th>Material</th><th>Description</th><th>Supplier</th><th>Use</th></tr>
<tr><td>ERM(R) – EC681m</td><td>Polyethylene with elements at high levels</td><td>European Joint Research Center (JRC) – Certified Reference Materials Catalogue
https://crm.jrc.ec.europa.eu/p/40455/40468/By-material-matrix/Other-manufactured-materials/ERM-EC681m-POLYETHYLENE-elements-high-level/ERM-EC681m</td><td>XRF calibration validation:
<table>
<tr><th>Element</th><th>Level [mg/kg]</th></tr>
<tr><td>As</td><td>17 ± 1.2</td></tr>
<tr><td>Br</td><td>1430 ± 80</td></tr>
<tr><td>Cd</td><td>146 ± 5</td></tr>
<tr><td>Cl</td><td>380 ± 60</td></tr>
<tr><td>Cr</td><td>45.1 ± 1.9</td></tr>
<tr><td>Hg</td><td>9.9 ± 0.8</td></tr>
<tr><td>Pb</td><td>69.7 ± 2.5</td></tr>
<tr><td>S</td><td>640 ± 100</td></tr>
<tr><td>Sb</td><td>86 ± 7</td></tr>
</table>
(real value within 20% of measured value)</td></tr>
<tr><td>SPEX Certiprep – CRM-PVC001</td><td>PVC with ortho-phthalates (3000-30000 µg/g)</td><td>Spex CertiPrep
https://www.spex.com/Product/Detail/Plastic-Standards-and-Additives/00a6b55b-3f70-43ed-a24d-7fc613abe41c/Phthalates-Polyvinyl-Chloride-Standard</td><td>ortho-phthalate calibration validation:
<table>
<tr><th>Substance</th><th>Level [mg/kg]</th></tr>
<tr><td>DMP</td><td>3’000</td></tr>
<tr><td>DEP</td><td>3’000</td></tr>
<tr><td>DBP</td><td>3’000</td></tr>
<tr><td>BBP</td><td>3’000</td></tr>
<tr><td>DBP</td><td>3’000</td></tr>
<tr><td>DNOP</td><td>3’000</td></tr>
<tr><td>DEHP</td><td>3’000</td></tr>
<tr><td>DiNP</td><td>30’000</td></tr>
<tr><td>DiDP</td><td>30’000</td></tr>
</table>
(real value within 20% of measured value)</td></tr>
</table>

Table S 4: Overview over employed standards and in which workflow they were used (q = quantification of *ortho*-phthalates, s = suspect screening). The table is sorted based on the substance group and the molecular weight.

**Substance name**	**Abbr.**	**CASRN**	**MW [g/mol]**	**Workflow**	**Supplier**
ortho-Phthalates					
Dimethyl phthalate	DMP	131-11-3	194.06	q,s	Sigma-Aldrich: 41320 (Lot:BCBZ7340)
Diethyl phthalate	DEP	84-66-2	222.09	q,s	Sigma-Aldrich: 53008 (Lot:BCBV6074)
Diallyl phthalate	DAP	131-17-9	246.09	q,s	Sigma-Aldrich: 36925 (Lot:BCBS8034V)
Diisobutyl phthalate	DiBP	84-69-5	278.15	q,s	CHEM Service: N-11589-1G (Lot:7047300)
Di-n-butyl phthalate	DBP	84-74-2	278.15	q,s	Sigma-Aldrich: 43540 (Lot:BCBV9941)
Bis(-2-methoxyethyl) phthalate	DMEP	117-82-8	282.11	q,s	CHEM Service: N-11304-500MG (Lot:6923600)
Diisopentyl phthalate	DiPP	605-50-5	306.18	q,s	CHEM Service: N-11620-500mg (Lot:7060400)
Isopentylpentyl phthalate	nPiPP	776297-69-9	306.18	q,s	CHEM Service: N13811-1G (Lot:6777200)
Di-n-pentyl phthalate	DPP	131-18-0	306.18	q,s	synthonix: P59310 (Lot:994)
Benzyl butyl phthalate	BBP	85-68-7	312.14	q,s	CHEM Service: N11360-1G (Lot:6894600)
Dicyclohexyl phthalate	DCHP	84-61-7	330.18	q,s	Aldrich: 306150 (Lot:09019JD)
Dihexyl phthalate	DHP	84-75-3	334.21	q,s	CHEM Service: N-11596-1G (Lot:6748400)
Di(2-ethylhexyl) phthalate	DEHP	117-81-7	390.28	q,s	CHEM Service: N11226-1G (Lot:6962500)
Dioctyl phthalate	DNOP	117-84-0	390.28	q,s	Sigma-Aldrich: 88173 (Lot:BCBV7232)
Diisononyl phthalate	DiNP	68515-48-0	418.31	q,s	Aldrich: 376663 (Lot:STBH9661)
Diisodecyl phthalate	DiDP	68515-49-1	446.34	q,s	Sigma-Aldrich: 80135 (Lot:BCCB0561)
Deuterated ortho-Phthalates					
LGC phthalates mixture	LGC	n.a.	n.a.	q	LGC: DRE-A50000576DI (Lot: -)
Deuterated(d4) diisobutyl phthalate	DiBP-d4	358730-88-8	282.18	q	CHIRON: 3123.16-100-IO (Lot:8282)
Deuterated(d4) di-n-butyl phthalate	DBP-d4	93952-11-5	282.18	q	CHEM Service: N-FD68-C-0.25G (Lot:7108100)
Deuterated(d4) diisopentyl phthalate	DiPP-d4	1346597-80-5	310.21	q	CHEM Cruz: SC-498746 (Lot:B0818)
Deuterated(d4) dipentyl phthalate	DPP-d4	358730-89-9	310.21	q	CHIRON: 2893.18-100-IO (Lot:13203)
Deuterated(d4) benzyl butyl phthalate	BBP-d4	93951-88-3	316.16	q	CHEM Service: S-FD67S-1.2ML (Lot:7108200)
Deuterated(d4) dihexyl phthalate	DHP-d4	1015854-55-3	338.24	q	CHIRON: 9367.20-100-IO (Lot:11572)
Deuterated(d4) di(2-ethylhexyl) phthalate	DEHP-d4	93951-87-2	394.30	q	CHEM Service: N-FD66-C-0.25G (Lot:7109200)

Table S 4 - continued

Substance name	Abbr.	CASRN	MW [g/mol]	Workflow	Supplier
Alternative plasticizers					
Bis(2-ethylhexyl) adipate	DEHA	103-23-1	370.31	s	Sigma-Aldrich: 442492 (Lot:LRAC6049)
Bis(2-ethylhexyl) terephthalate	DEHT	6422-86-2	390.28	s	Sigma-Aldrich: 49234-1mL (Lot:BCCD3728)
1,2-Cyclohexane dicarboxylic acid diisononyl ester	DINCH	166412-78-8	424.36	s	European Pharmacopoeia Ref. Std.: Y0002022 (Lot:2)
Phosphate plasticiers / flame retardants					
Tributylphosphate	TBP	126-73-8	266.16	s	Aldrich: 240494 (Lot:MKBL5358V)
Tris-(2-chloroethyl) phosphate	TCEP	115-96-8	283.95	s	Aldrich: 119660 (Lot:U08057V)
Tris-(2-chloroisopropyl) phosphate	TCPP	13674-84-5	326.00	s	Fluka: TCPP / 32952 (Lot:SZBC180XV)
Triphenyl phosphate	TPhP	115-86-6	326.07	s	Aldrich: 241288 (Lot:BCBM3828V)
2-Ethylhexyl diphenyl phosphate	Octicizer	1241-94-7	362.16	s	Sigma: 34064 (Lot:SZBE274XV)
Tricresyl phosphate	TCP	1330-78-5	368.12	s	Aldrich: 268917 (Lot:30696EKV)
Tri(3,4-dimethylphenyl)phosphate	TMPP	3862-11-1	410.16	s	Aldrich: S365378 (Lot:-)
Tri(2,4-dimethylphenyl)phosphate	TXP	3862-12-2	410.16	s	Aldrich: S405752 (Lot:1636204)
Tris(1,3-dichloro-2-propyl)phosphate	TDCPP	13674-87-8	427.88	s	Aldrich: TDCPP (Lot:SZBE090XV)
Tris(2-ethylhexyl) phosphate	TEHP	78-42-2	434.35	s	Sigma: 289922 (Lot:S44036V)
Tris(2,3-dibromopropyl) phophate	TBPP	126-72-7	691.58	s	Chem Service: N-13722-100MG (Lot:9772000)
Brominated flame retardants					
2,4,6-Tribromophenol	TBPh	118-79-6	327.77	s	Aldrich: 137715 (Lot:29699MJV)
2,2',4,4' - Tetrabromodiphenyl ether	BDE47	5436-43-1	481.72	s	Wellington: TetraBDE / BDE-47 (Lot:BDE470409)
3,3',5,5'-Tetrabromobisphénol A	TBBPA	79-94-7	539.76	s	Wellington: TBBPA (Lot:TBBPA0114)
γ-1,2,5,6,9,10 - Hexabromocyclododecane	gHBCD	134237-52-8	635.65	s	Wellington: g-HBCD (Lot:gHBCD1119)
2,2′,3,4,4′,5′,6-Heptabromdiphenylether	BDE183	207122-16-5	715.45	s	Wellington: HeptaBDE / BDE-183 (Lot:BDE1830611)
Antioxidants					
δ-Tocopherol	dToc	119-13-1	402.35	s	Sigma-Aldrich: 47784 (Lot: -)
Irganox 1035	1035	41484-35-9	642.40	s	Sigma-Aldrich: BL3H160C36E1 (Lot: -)
Bisphenols					
Bisphenol-A	BPA	80-05-7	228.12	s	CHEM Service: N-12907-100MG (Lot:6606700)
Bisphenol-S	BPS	80-09-1	250.03	s	CHEM Service: N-14105-100MG (Lot:7060500)
Solvents					
Methanol	MeOH	67-56-1	32.04	q,s	Merck: 34860 (Lot: -)
Acetonitrile	ACN	75-05-8	41.05	q,s	Merck: 1155002500 (Lot: I640800 232)
Tetrahydrofuran	THF	109-99-9	72.11	q,s	Honeywell: 34865-1L (Lot: L348M)
n-Hexane	n-Hex	110-54-3	86.18	q,s	Sigma-Aldrich: 139386 (Lot: -)
Toluene	Tol	108-88-3	92.14	q,s	Merck: 34866 (Lot: -)

S2.3 Chemical analysis

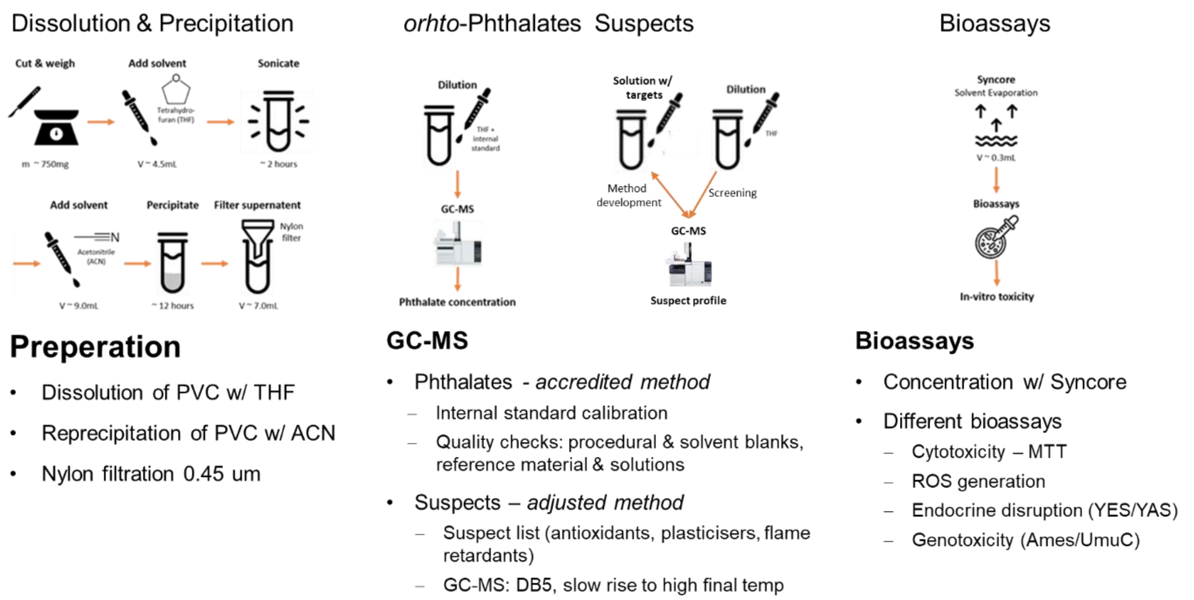

Figure S 2: Sample preparation and overview for GC-MS *ortho*-phthalate quantifcaiton, GC-MS suspect screening and testing of biological activities.

The conducted analyses use different types of sample processing which may impact the detected substances or effects. While XRF and FTIR are surface-specific techniques and thus need minimal processing, they but only yield information on the layer on top or bottom of the sample. By contrast, GC-MS and the bioassays required the extraction of compounds of interest from the polymer matrix but and yield results from the entire sample, furthermore bioassays required solvent evaporation removing any very volatile substances. For the results from surface-specific techniques and extraction techniques to be comparable, it has to be assumed that substances need to be equally dispersed in the product.

S2.3.1 ATR-FTIR

An ATR-FTIR spectrum was recorded for each sample and each side using a Thermo Scientific Nicolet™ iS spectrometer with iD7 ATR accessory (settings in Table S 5). All recorded spectra can be found in SI6-Rawdata-ATR-FTIR. No sample pre-treatment was made, apart from cleaning the sample surface with ethanol where necessary.

Table S 5: ATR-FTIR settings used to determine the polymer type and the presence of *ortho*-phthalates.

**parameter**	**value**
**Spectral Range [cm^{-1}]**	500 - 4000
**Number of scans per sample**	15

Polymer type determination: The polymer type was determined using the ThermoFischer OMNIC Spectra Polymer Package and selected reference spectra.[12,13] Non-PVC samples (n=35) were not analyzed further.

***ortho*-Phthalates screening**: The presence of *ortho*-phthalates was determined using the characteristic *ortho*-phthalate peaks at 1600cm^{-1} and 1580cm^{-1}, with an approximate sensitivity according to the instrument manufacturer of 0.1weight% of *ortho*-phthalates.[14,15] The quality of the screening was compared to the GC-MS quantification *ortho*-phthalates (Table S 6). FTIR screening detected the majority of samples containing *ortho*-phthalates (sensitivity: 78.2%), and almost all samples containing more than 0.1wt% of *ortho*-phthalates (sensitivity: 97.2%), without many false positives (specificity: 85.4% resp. 80.9%).

Table S 6: Quality of ATR-FTIR screening for *ortho*-phthalates, using confusion matrices, sensitivity and specificity.

<table>
<tr><th>Any o-phthalates</th><th>High amounts (>0.1wt%) of o-phthalates</th></tr>
<tr><td>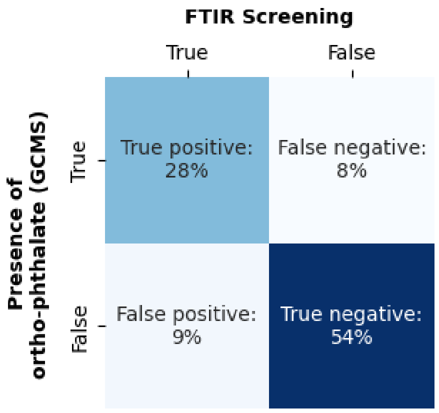
</td><td>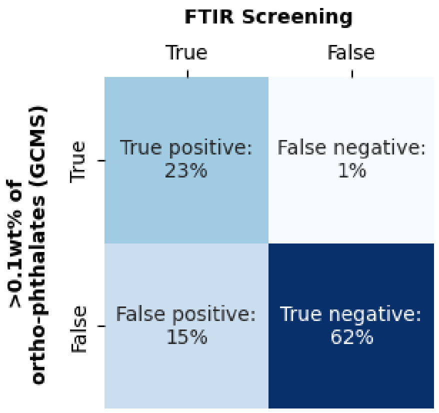
</td></tr>
<tr><td>Sensitivity:78.2%
Specificity:85.4%</td><td>Sensitivity:97.2%
Specificity:80.9%</td></tr>
</table>

S2.3.2 XRF elemental composition

The elemental composition of the samples was determined using a handheld XRF (Thermo Scientific™ Niton™ XL3 Gold Analyzer) with a plastic calibration. No specific sample pre-treatment was made, apart from cleaning the sample surface with ethanol where necessary. Each side was measured for at least 30 seconds with each filter. Correct operation and equipment calibration was checked using a certified reference material, ERM-EC681m – Polyethylene (high level): the measured concentrations had to be within 20% of the certified levels. The XRF's limits of detection (LODs) are calculated according to the instrument manufacturer's protocol, as three times the minimum standard deviation of the analyte.[16] The calculated LODs and the LODs reported by the manufacturer are provided in Table S 7. Concentrations and standard deviations were noted as determined by the instrument's plastic calibration, the original fluorescence spectra were not exported.

Table S 7 Limits of detection (LODs) for the Niton XL3 GOLDD XRF for PVC matrices for a 30-second analysis time per filter, calculated from our measurements and under ideal conditions according to the instruments plastic calibration.

Element	Number samples for LOD determination	LOD, calculated [mg/kg]	LOD, reported [mg/kg]
**As**	408	5	15
**Au**	418	17	
**Ba**	161	67	100
**Bi**	408	9	
**Br**	319	4	8
**Cd**	411	13	15
**Cl**	60	250	
**Cr**	363	11	20
**Cu**	393	18	
**Fe**	183	33	
**Hg**	413	12	25
**Ni**	376	14	
**Pb**	371	6	15
**Sb**	387	24	25
**Se**	418	7	20
**Sn**	268	18	
**Ti**	176	24	
**V**	276	10	
**Zn**	121	17	

S2.3.3 GC-MS quantification of phthalates

The official laboratory protocol was in French and was translated by DEEPL to English for better understanding of the reader. Both original and English version can be found in SI3.

Sample preparation. The samples were cut into smaller pieces and weighed exactly (~750mg), dissolved in a weighed amount of *tetrahydrofuran* (*THF*, CASRN: 109-99-9, ~4.5 mL) using an ultrasound bath for about two hours at room temperature. After adding a weighted amount of *acetonitrile* (*ACN*, CASRN: 75-05-8, ~9 mL), samples were left in the fridge (4°C) overnight for the polymers to re-precipitate, and subsequently filtered using 0.45um nylon filters (BGB SF2503-2). The resulting filtrate had a known concentration of PVC at ~55 mg/mL. Subsequently, the extracts were diluted using THF to two levels (40-fold and 1600-fold dilution) and spiked with the internal standards. Sample preparation was conducted in batches due to spatial and temporal constraints. For each batch, a procedural blank containing no PVC sample and a PVC reference material (SPEX CRM-PVC001) with certified levels of *ortho*-phthalate was prepared analogously to the samples.

GC-MS analysis. Seventeen *ortho*-phthalates were used as standards for the calibration curves (Table S 4, Table S 8), and seven deuterated *ortho*-phthalates were used as internal standards (Table S 4). The calibration curve spanned points from 0.05 to 10 mg/mL for most standards (for *DiNP* and *DiDP*, it spanned 0.5 to 100 mg/mL). GC-MS analysis was conducted in batches to ensure proper operation. Besides calibration solutions and samples, each batch also contained a blank solution, a reference solution with a known concentration, and the solutions from the procedural blank and the reference material.

Briefly, all analyses were carried out on an Agilent GC-MS system (GC: Agilent 7890A, MS: Agilent 5975C) in single ion mode (SIM) with splitless injections with internal standard calibration. The compounds were separated on a DB 5MS column using a temperature gradient from 80°C to 320°C. The injection was performed in pulsed splitless mode (injection volume: 2 uL), to a wool-filled liner (Topaz, 4mm Single Taper w/Wool) to avoid build-up of dissolved short-chain PVC on the column. The compounds were separated on a DB-5MS column (length: 15 m, inner diameter: 0.25 mm, film thickness: 0.1 mm), using Helium as a carrier gas (constant flow rate: 1mL/min). The oven temperature was set from 80°C (initial hold: 2 min) to 320°C with a changing temperature gradient (20°C/min until 200°C, 8°C/min until 320°C). The interface temperature was set to 280°C.

Ionization was done by electron impact (Ionisation energy: 70 eV, Ion source temperature: 250°C). The MS was set to SIM mode with several retention time windows, with a quantification- and a control-ion for each calibration standard or internal standard eluting within a given window (see Table S 8 for retention time windows and the target ions for each standard). To preserve the detector, a solvent delay was set to 3.5 minutes and the 1600-fold dilutions were run first, and 40-fold dilutions were only run if no signal was recorded.

The chromatogram of the *ortho*-phthalate standards can be found below (Figure S 3). The retention time and calibration curves are listed in Table S 8.

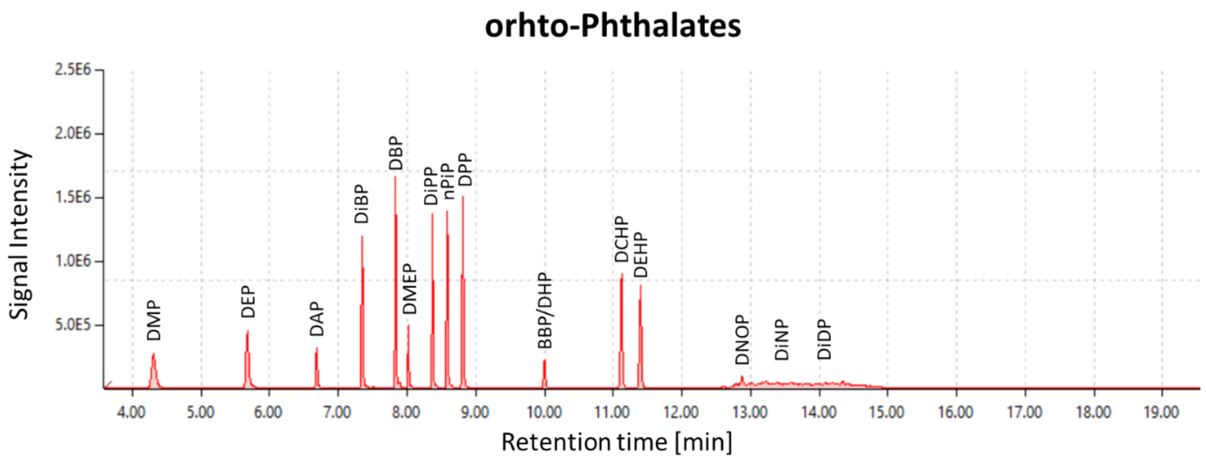

Figure S 3: Chromatogram of all *ortho*-phthalate standards at ~5 µg/mL (DiNP and DiDP at ~50 µg/mL) using the *ortho*-phthalate quantification workflow.

Data Analysis. The recorded spectra were analyzed in an automatic quantitative workflow using Agilent Masshunter (the raw data are available as Agilent files in SI7-Rawdata-GCMS-Phthalates). Quadratic and weighted calibration curves using the relative signal of calibration standard to internal standard were used (Weight: 1/x). The automatic integration, the calibration curves, and the quantification of blanks, reference solution, and reference material, were double-checked manually.

Quality assurance and control (QA/QC). Quality assurance and control were implemented throughout the process. Specific quality management practices in the accredited laboratory were observed, including regular maintenance of the GC-MS and replacement of liners and septa, daily tune evaluation to ensure correct MS detection, analysis of blanks and references solutions to ensure correct GC-MS operation, analysis of procedural blanks and certified reference material to ensure correct extraction, and manual checks of the automatic data analysis workflow. For the

quality control, measured concentrations of the reference solutions and certified reference material had to be within 20% of the certified levels

 https://doi.org/10.1021/acs.est.3c04851

S2.3.3.1 *Target compounds*

Table S 8: *ortho*-Phthaltate standards used in the *ortho*-phthalate quantification workflow. Calibration curves were fitted to a linear model (Relative area = a0 + a1 *concentration) and a quadratic model (Relative area = a0 + a1* concentration + a2*concentration^2). The calibration was redone for each run (the displayed calibration curves were extracted from the run "220517_Batch7-int"). The table is sorted based on retention time (RT). RT= retention time, Q-ion = Quantification ion, C-ion = Control ion, CASRN = Chemical Abstract Service Registry Number, MW = Molecular weight of isotope, Dyn. Range = Dynamic range.

Substance name	Structure	RT [min]	window [min]	Q-ion [m/Z]	C-ion [m/Z]	ISTD	Calibration curve (with ISTD)
**DMP**<br>Dimethyl phthalate<br>CASRN: 131-11-3<br>MW: 194.06 g/mol	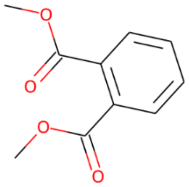	4.33	3.5 → 5.0	163.0	*194.0*	*DiBP-d4*	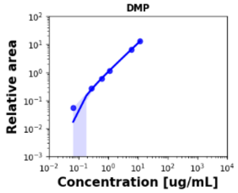<br>*Regressions:*<br>***Model*** ***a0*** ***a1*** ***a2*** ***R2***<br>***quad*** *-0.02* *1.04* *0.005* *1*<br>***lin*** *-0.06* *1.09* *n.a.* *1*<br>*Dyn. range:* 0.07 - 11.87 µg/mL
**DEP**<br>Diethyl phthalate<br>CASRN: 84-66-2<br>MW: 222.09	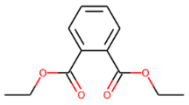	5.68	5.00 → 6.50	149.0	177.0	*DiBP-d4*	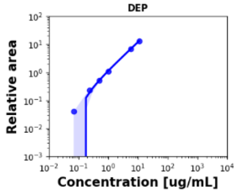<br>*Regressions:*<br>***Model*** ***a0*** ***a1*** ***a2*** ***R2***<br>***quad*** *-0.04* *1.12* *0.008* *1*<br>***lin*** *-0.09* *1.21* *n.a.* *1*<br>*Dyn. range:* 0.07 - 10.88 µg/mL
**DAP**<br>Diallyl phthalate<br>CASRN: 131-17-9<br>MW: 246.09	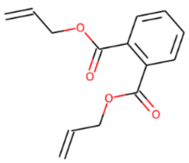	6.68	6.50 → 7.20	149.0	189.0	*DiBP-d4*	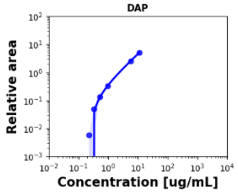<br>*Regressions:*<br>***Model*** ***a0*** ***a1*** ***a2*** ***R2***<br>***quad*** *-0.09* *0.43* *0.003* *1*<br>***lin*** *-0.12* *0.46* *n.a.* *1*<br>*Dyn. range:* 0.07 - 11.15 µg/mL
**DiBP**<br>Diisobutyl phthalate<br>CASRN: 84-69-5<br>MW: 278.15	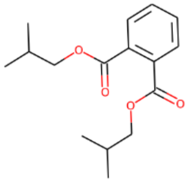	7.35	7.20 → 7.70	149.1	223.1	*DiBP-d4*	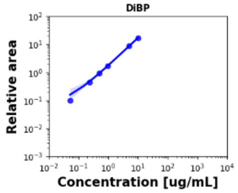<br>*Regressions:*<br>***Model*** ***a0*** ***a1*** ***a2*** ***R2***<br>***quad*** *0.004* *1.80* *-0.011* *1*<br>***lin*** *0.07* *1.69* *n.a.* *1*<br>*Dyn. range:* 0.05 - 9.96 µg/mL

https://doi.org/10.1021/acs.est.3c04851

Substance name	Structure	RT	window	Q-ion	C-ion	ISTD	Calibration curve (with ISTD)
**DBP**<br>Di-n-butylphthalate<br>CASRN: 84-74-2<br>MW: 278.15	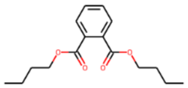	7.83	7.70 → 8.00	149.1	223.1	*DBP-d4*	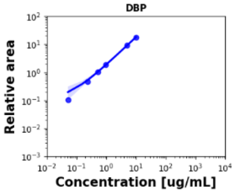<br>*Regressions:*<br>***Model*** ***a0*** ***a1*** ***a2*** ***R2***<br>***quad*** *0.003* *1.95* *0.016* *1*<br>***lin*** *0.10* *1.79* *n.a.* *0.999*<br>*Dyn. range:* 0.05 - 9.98 µg/mL
**DMEP**<br>Bis(-2-methoxyethyl) phthalate<br>CASRN: 117-82-8<br>MW: 282.11	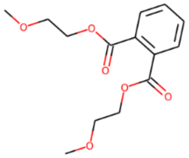	8.02	8.00 → 8.30	59.1	149.0	*DiPP-d4*	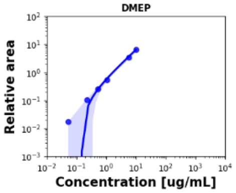<br>*Regressions:*<br>***Model*** ***a0*** ***a1*** ***a2*** ***R2***<br>***quad*** *-0.009* *0.50* *0.015* *1*<br>***lin*** *-0.10* *0.65* *n.a.* *0.997*<br>*Dyn. range:* 0.05 - 9.85 µg/mL
**DiPP**<br>Diisopentyl phthalate<br>CASRN: 605-50-5<br>MW: 306.18	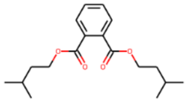	8.37	8.30 → 8.55	149.0	237.1	*DiPP-d4*	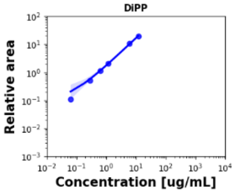<br>*Regressions:*<br>***Model*** ***a0*** ***a1*** ***a2*** ***R2***<br>***quad*** *-0.006* *1.80* *-0.011* *1*<br>***lin*** *0.010* *1.66* *n.a.* *0.999*<br>*Dyn. range:* 0.06 - 12.04 µg/mL
**nPiPP**<br>Isopentylpentyl phthalate<br>CASRN: 776297-69-9<br>MW: 306.18	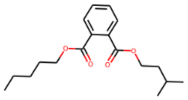	8.59	8.55 → 8.75	149.0	237.1	*DPP-d4*	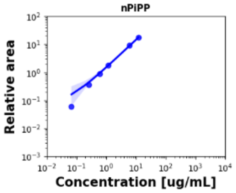<br>*Regressions:*<br>***Model*** ***a0*** ***a1*** ***a2*** ***R2***<br>***quad*** *-0.05* *1.59* *-0.012* *1*<br>***lin*** *0.06* *1.44* *n.a.* *0.999*<br>*Dyn. Range: 0.07 - 12.12 µg/mL*
**DPP**<br>Di-n-pentyl phthalate<br>CASRN: 131-18-0<br>MW: 306.18	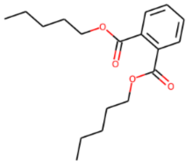	8.81	8.75 → 9.20	149.1	237.1	*DPP-d4*	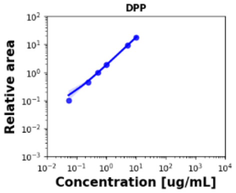<br>*Regressions:*<br>***Model*** ***a0*** ***a1*** ***a2*** ***R2***<br>***quad*** *-0.004* *1.84* *-0.009* *1*<br>***lin*** *0.06* *1.75* *n.a.* *1*<br>*Dyn. range:* 0.05 - 10.31 µg/mL
**BBP**<br>Benzyl butyl phthalate<br>CASRN: 85-68-7<br>MW: 312.14	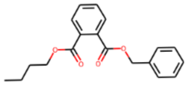	10.00	9.20 → 10.80	206.1	238.0	BBP-d4	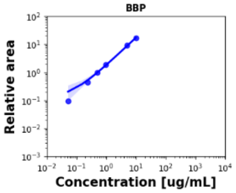<br>*Regressions:*<br>***Model*** ***a0*** ***a1*** ***a2*** ***R2***<br>***quad*** *-0.005* *1.91* *-0.019* *1*<br>***lin*** *0.11* *1.72* *n.a.* *0.999*<br>*Dyn. range:* 0.05 - 9.98 µg/mL

https://doi.org/10.1021/acs.est.3c04851

<table>
<tr><th>Substance name</th><th>Structure</th><th>RT</th><th>window</th><th>Q-ion</th><th>C-ion</th><th>ISTD</th><th>Calibration curve (with ISTD)</th></tr>
<tr><td>DHP
Dihexyl phthalate
/CASRN: 84-75-3
MW: 334.21</td><td>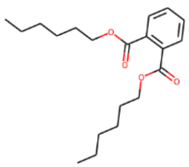</td><td>10.00</td><td>9.20 → 10.80</td><td>251.1</td><td>233.1</td><td>DHP-d4</td><td>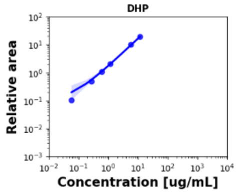

Regressions:
Model a0 a1 a2 R2
quad -0.002 1.78 0.012 1.000
lin 0.10 1.64 n.a. 0.999
Dyn. range: 0.06 - 11.64 µg/mL</td></tr>
<tr><td>DCHP
Dicyclohexyl phthalate
CASRN: 84-61-7
MW: 330.18</td><td>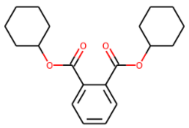</td><td>11.12</td><td>10.80 → 11.35</td><td>149.0</td><td>167.0</td><td>DEHP-d4</td><td>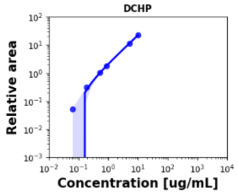

Regressions:
Model a0 a1 a2 R2
quad -0.08 2.08 0.015 1
lin -0.17 2.22 n.a. 1
Dyn. range: 0.06 - 9.96 µg/mL</td></tr>
<tr><td>DEHP
Di(2-ethylhexyl) phthalate
CASRN: 117-81-7
MW: 390.28</td><td>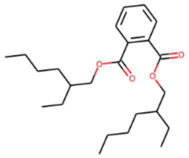</td><td>11.39</td><td>11.35 → 11.90</td><td>149.0</td><td>167.0</td><td>DEHP-d4</td><td>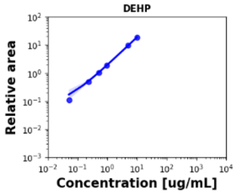

Regressions:
Model a0 a1 a2 R2
quad 0.007 1.97 -0.011 1
lin 0.07 1.86 n.a. 1
Dyn. range: 0.05 - 9.97 µg/mL</td></tr>
<tr><td>DNOP
Dioctyl phthalate
CASRN: 117-84-0
MW: 390.28</td><td>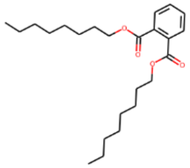</td><td>12.88</td><td>11.90 → end</td><td>279.1</td><td>261.1</td><td>DEHP-d4</td><td>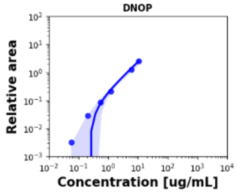

Regressions:
Model a0 a1 a2 R2
quad -0.006 0.16 0.008 1
lin -0.06 0.24 n.a. 0.993
Dyn. range: 0.06 - 10.48 µg/mL</td></tr>
<tr><td>DiNP
Diisononyl phthalate
CASRN: 68515-48-0
MW: 418.31</td><td>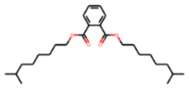
+ isomers</td><td>12.1-15.1</td><td>11.90 → end</td><td>293.2</td><td>127.1</td><td>DEHP-d4</td><td>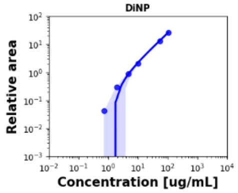

Regressions:
Model a0 a1 a2 R2
quad -0.11 0.22 0.0004 1
lin -0.37 0.26 n.a. 0.998
Dyn. range: 0.7 - 103.4 µg/mL</td></tr>
<tr><td>DiDP
Diisodecyl phthalate
CASRN: 68515-49-1
MW: 446.34</td><td>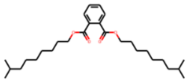
+ isomers</td><td>12.5-15.6</td><td>11.90 → end</td><td>307.2</td><td>289.2</td><td>DEHP-d4</td><td>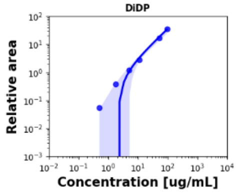

Regressions:
Model a0 a1 a2 R2
quad -0.07 0.24 0.001 1
lin -0.79 0.37 n.a. 0.992
Dyn. range: 0.5 - 93.9 µg/mL</td></tr>
</table>

https://doi.org/10.1021/acs.est.3c04851

S2.3.4 GC-MS suspect screening

Sample preparation. The same extraction procedure and dilutions as above were used (section S2.3.3), but without adding internal standards.

Suspect substances and custom library: Common alternative plasticizers and some antioxidants were used as suspect substances, for which analytical standards were used (Table S 4). For example, *DEHT* [*Bis(2-ethylhexyl) terephthalate*, CASRN: 6422-86-2], *DINCH* [*Di(isononyl) cyclohexane-1,2-dicarboxylate*, CASRN: 166412-78-8], *DEHA* [*Bis(2-ethylhexyl) adipate*, CASRN: 103-23-1], *TPhP* [*Triphenyl Phosphate*, CASRN: 115-86-6], *TCP* [*Tricresyl phosphate*, CASRN: 78-32-0], and *Octicizer* (*2-Ethylhexyl diphenyl phosphate*, CASRN: 1241-94-7) were used.

The suitability of the extraction procedure for the suspects was ensured (1) by doing a simple solubility check in relevant solvent systems (THF, 1:2 THF:ACN) and (2) by spiking a PVC sample and following the regular extraction procedure. Semi-quantification (based on a signal calibration curve) and approximate detection limits (based on the lowest concentration with correct identification) were determined using a dilution series for the suspect standards (see below).

An Agilent custom library was created from their measured mass spectra at 5 mg/L (SI4). The chromatogram of the suspect standards can be found below (Figure S 3), their retention time and mass spectra are in Table S 8.

A dilution series (different dilutions) of the investigated standards were run to determine (a) approximate detection limits and (b) approximate calibration curves for the semi-quantification. This semi-quantification is more uncertain compared to the phthalate quantification as:

(1) no internal standard was used and the MS response of a standards may depend on various external factors other than the concentration,
(2) fewer concentration-response data points were collected for most standards as the aim of this was not proper quantification
(3) the dilutions of our samples did not always fall within the dynamic range of our approximate calibration.

Overall, most standards had an approximate calibration slope (Area/concentration in µg/L) of 7.4 ± 9.8 x 10^5 (1.3 x 10^2 – 3.3 x 10^6), the detector response for *ortho*-phthalates was generally higher than for other standards (Figure S 6).

GC-MS analysis: All measurements were conducted on a low-resolution Agilent GC-MS system (GC: Agilent 7890A, MS: Agilent 5975C) in scan mode. The injection was performed in splitless mode (injection volume: 2uL, injection temp: 140°C), to a wool-filled liner (Topaz, 4mm Single Taper w/Wool) to avoid build-up of dissolved short-chain PVC on the column. The compounds were separated on a DB-5MS column (length: 15 m, inner diameter: 0.25 mm, film thickness: 0.1 mm), using Helium as a carrier gas (flow rate: 1mL/min). The oven temperature was set from 40°C (initial hold: 2 min) to 300°C (final hold: 20 min) with a change of 8°C/min. The interface temperature was set to 280°C. Ionization was done by electron impact (Ionisation energy: 70 eV, Ion source temperature: 250°C). The MS was set to scan mode with a range of 30 - 800 amu (scan speed: 1.2 scan/s). To preserve the detector, the solvent delay was set to 8 minutes and the 1'600-fold dilutions were run first, and 40-fold dilutions were only run if a low signal was recorded.

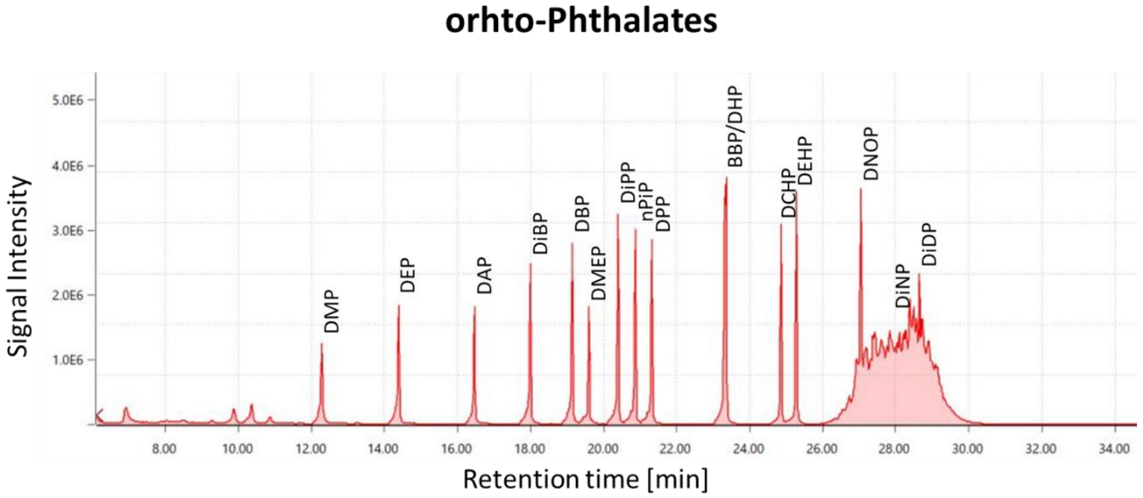

Figure S 4: Chromatogram of all *ortho*-phthalate standards (PHT solution) using the suspect screening workflow

 https://doi.org/10.1021/acs.est.3c04851

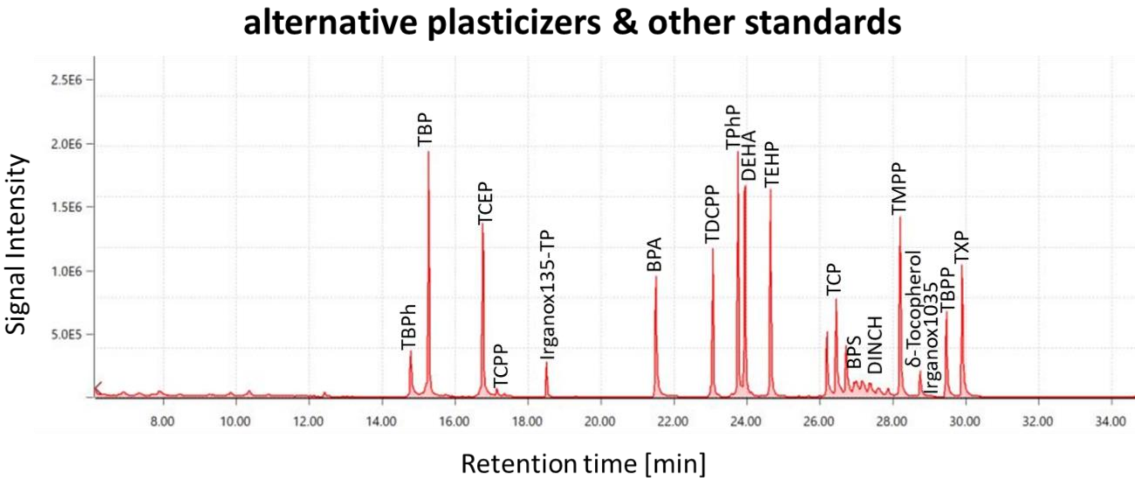

Figure S 5: Chromatogram of alternative plasticizer standards (Add solution) using the suspect screening workflow

Data analysis. All recorded chromatograms and mass spectra (available as Agilent files in SI8-Rawdata-GCMS-Suspect) were analyzed for the presence and approximate concentration of the suspect compounds, and for unknown substances using library identification. A qualitative Agilent Masshunter workflow was used for compound discovery (either using chromatogram integration or molecular feature) and compound identification (using the custom library first, and the NIST 14 library second) with the final output exported as an Excel file. For compound discovery, both "Find by integration" (considering all Lorentzian chromatogram peaks with an area larger than 0.001% of the largest peak) and "Find by molecular feature" (limited to Lorentzian peaks with more than 500 counts and the largest 200 compounds) were used. For compound identification, (1) a manually created suspect library of the scanned suspect standards was searched first and then (2) the NIST 14 library was searched (this old library version was used to limit overfitting the data). The suspect library was constructed from measurements of the suspect standards at 5 mg/L. The minimum matching score for both libraries was set to 50, but was usually above 70; only *TMPP* [*Tri(3,4-dimethylphenyl) phosphate*, CASRN: 3862-11-1], *TCPP* [*Tris-(2-chloroisopropyl) phosphate*, CASRN: 13674-84-5] and *DINCH* scored slightly below 70. The assignment of suspects was partially manually double-checked, based on retention time. Substances that appeared several times under different identifiers in the library were manually harmonized (e.g., *DEHT* appears in the NIST library under the CASRN "6422-86-2" or under the name "*1,4-Benzenedicarboxylic acid, 1,4-bis(2-ethylhexyl) ester*").

Further data processing was done in Python (SI5) and included:

1. combining the individual Excel files
2. flagging compounds discovered in procedural blanks as “Blanks”
3. assigning identification confidence of compounds (confirmed with standards as “Level 1 – standard confirmed”, others as “Level 2 – library confirmed”)
4. ranking substances based on their importance (total signal area, number of samples)
5. semi-quantifying suspects based on the calibration curves from the dilution series (calibration curves in Figure S 6 for all standards, in Table S 10 for individual standard).

Samples were run at two dilutions, the final concentration was selected based on which detections were in range (the detailed algorithm is portrayed in Table S 9 and the final selected concentrations for the semi-quantification can be found in Sheet S8 in SI2).

QA/QC. The aforementioned QA/QC were also applied here. Furthermore, the workflow for the suspect substances was thoroughly pre-tested, including, (1) testing the suitability of the extraction procedure, (2) optimizing GC-MS and data analysis parameters, and (3) determining approximate LODs for all suspects. Blank samples and suspect standards were included in regular intervals to ensure correct GC-MS operation, and correct suspect identification was ensured by employing a costume suspect library with a matching score above 70 (in most cases) and manual checks.

Table S 9: Selection of most suitable value based on detection situation. Selected value is in bold, comment in normal text, color signifies possible mistakes

		**FINAL (1600x dilution)**			
		n.d.	*below range*	*in range*	*above range*
**INT (40x dilution)**	*n.d.*	**none:**<br>0, None detected	**FINAL**:<br>1, FINAL – below range	**FINAL**:<br>1, FINAL – in range	**FINAL**:<br>1, FINAL – above range
	below range	**INT**:<br>1, INT - below range	**Mean**:<br>2, Mean – both below range	**Mean**:<br>2, Mean - FINAL in range, INT below range	**Mean**:<br>2, Mean – FINAL above range, INT below range
	in range	**INT** –<br>1, INT - in range	**INT:**<br>2, INT – in range	**Mean**<br>2, Mean – both in range	**Mean**:<br>2, Mean - FINAL above range, INT in range
	above range	**INT** –<br>1, INT - above range	**Mean** –<br>2, Mean – FINAL below range, INT above range	**FINAL:**<br>2, FINAL – in range	**Mean** –<br>2, Mean – both above range

https://doi.org/10.1021/acs.est.3c04851

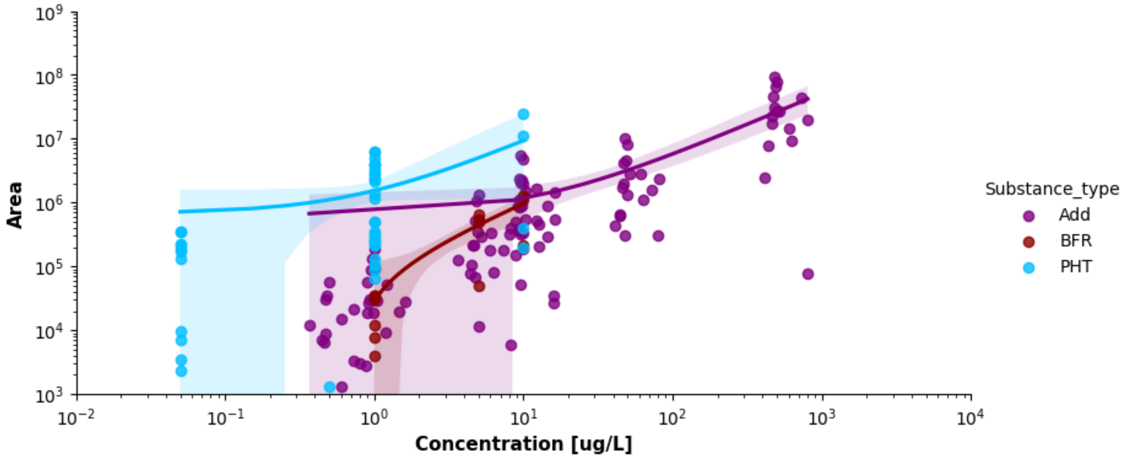

Figure S 6: Peak area vs concentration for different types of standards used in the suspect screening workflow.

S2.3.4.1 Suspect list

Table S 10: Analytical standards (including *ortho*-phthalates, alternative plasticizer, phosphate plasticizers, brominated flame retardants, antioxidants and bisphenols) used in the suspect-screening workflow. Overview of massspectra and approximate calibration curves for GC-MS suspect-screening. Approximate calibration curves were fitted to a constrained linear model with the intercept forced through zero (Area = a1*concentration) and a regular linear model (Area = a0 + a1 *concentration). The table is sorted based on substance group and retention time (RT). RT = Retention time, CASRN = Chemical Abstract Service Registry Number, MW = Molecular weight of isotope, Dyn. Range = Dynamic range.

Substance name	Structure	RT [min]	Massspectrum	Calibration curve	
ortho-Phthalates					
**DMP**<br>Dimethyl phthalate<br>CASRN: 131-11-3<br>MW: 194.06 g/mol	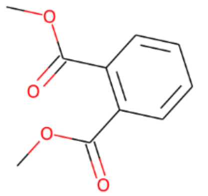	12.3	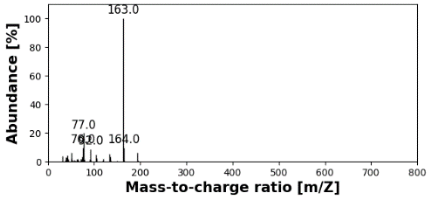	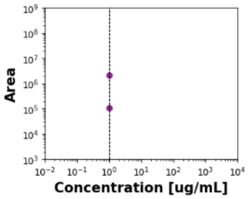	*Linear regressions:*<br>***Model*** ***a0*** ***a1*** ***R^2***<br>***lin*** *n.a.* *n.a.* *n.a.*<br>***constr.*** *-* *1.1e6* *n.a.*<br>*Dyn. range: > 1 µg/mL*
**DEP**<br>Diethyl phthalate<br>CASRN: 84-66-2<br>MW: 222.09 g/mol	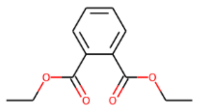	14.4	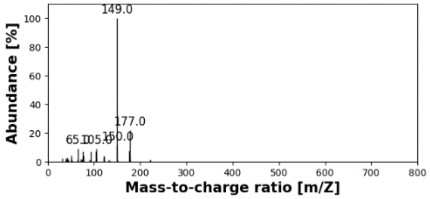	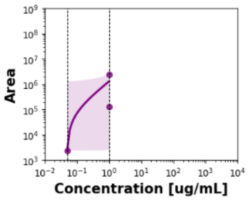	*Linear regressions:*<br>***Model*** ***a0*** ***a1*** ***R^2***<br>***lin*** *-6.5e4* *1.4e6* *0.999*<br>***constr.*** *-* *1.3e6* *0.290*<br>*Dyn. range: 0.05 – 1.00 µg/mL*
**DAP**<br>Diallyl phthalate<br>CASRN: 131-17-9<br>MW: 246.09 g/mol	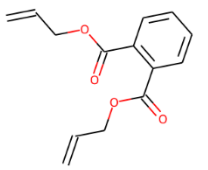	16.5	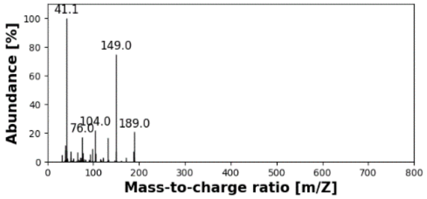	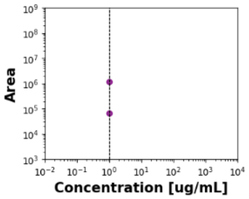	*Linear regressions:*<br>***Model*** ***a0*** ***a1*** ***R^2***<br>***lin*** *n.a.* *n.a.* *n.a.*<br>***constr.*** *-* *6.2e5* *n.a.*<br>*Dyn. range: > 1 µg/mL*
**DiBP**<br>Diisobutyl phthalate<br>CASRN: 84-69-5<br>MW: 278.15 g/mol	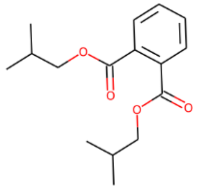	18.0	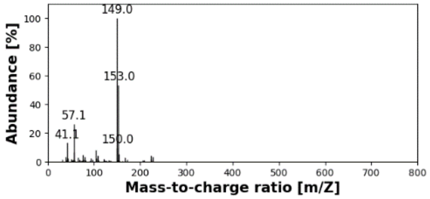	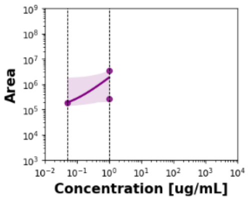	*Linear regressions:*<br>***Model*** ***a0*** ***a1*** ***R^2***<br>***lin*** *1.0e5* *1.7e6* *0.270*<br>***constr.*** *-* *1.8e6* *0.267*<br>*Dyn. range: 0.05 – 1.00 µg/mL*

https://doi.org/10.1021/acs.est.3c04851

Substance name	Structure	RT [min]	Massspectrum	Calibration curve	
**DBP**<br>Di-n-butylphthalate<br>CASRN: 84-74-2<br>MW: 278.15 g/mol	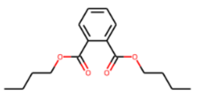	19.2	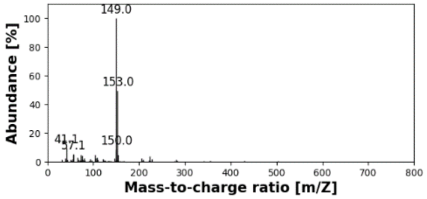	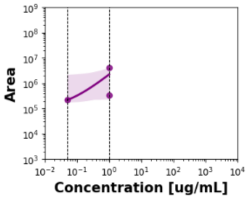	*Linear regressions:*<br>***Model*** ***a0*** ***a1*** ***R^2***<br>***lin*** 1.1e5 2.1e6 0.272<br>***constr.*** - 2.2e6 0.271<br><br>*Dyn. range:* 0.05 – 1.00 µg/mL
**DMEP**<br>Bis(-2-methoxyethyl) phthalate<br>CASRN: 117-82-8<br>MW: 282.11 g/mol	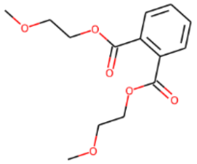	19.6	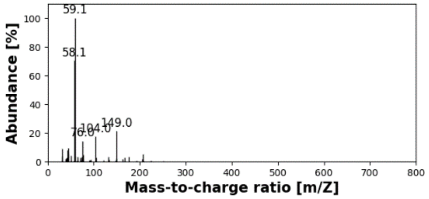	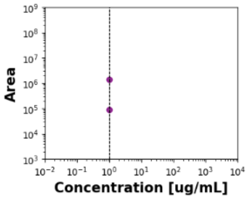	*Linear regressions:*<br>***Model*** ***a0*** ***a1*** ***R^2***<br>***lin*** *n.a.* *n.a.* *n.a.*<br>***constr.*** - *7.3e5* *n.a.*<br><br>*Dyn. range:* > 1 µg/mL
**DiPP**<br>Diisopentyl phthalate<br>CASRN: 605-50-5<br>MW: 306.18 g/mol	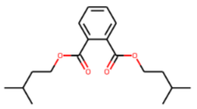	20.4	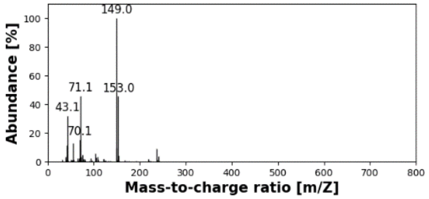	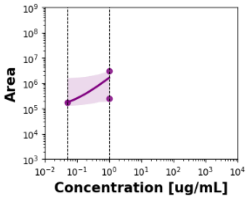	*Linear regressions:*<br>***Model*** ***a0*** ***a1*** ***R^2***<br>***lin*** 9.9e4 1.5e6 0.270<br>***constr.*** - 1.6e6 0.266<br><br>*Dyn. range:* 0.05 – 1.00 µg/mL
**nPiPP**<br>Isopentylpentyl phthalate<br>CASRN: 776297-69-9<br>MW: 306.18 g/mol	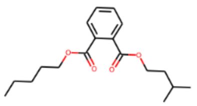	20.9	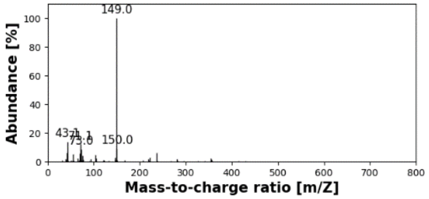	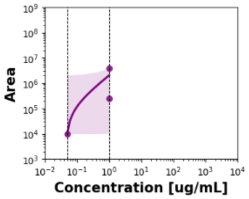	*Linear regressions:*<br>***Model*** ***a0*** ***a1*** ***R^2***<br>***lin*** -9.6e4 2.1e6 0.300<br>***constr.*** - 2.0e6 0.300<br><br>*Dyn. range:* 0.05 – 1.00 µg/mL
**DPP**<br>Di-n-pentyl phthalate<br>CASRN: 131-18-0<br>MW: 306.18 g/mol	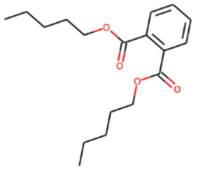	21.3	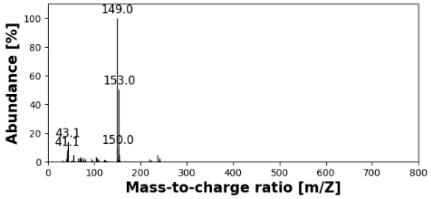	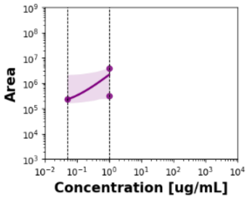	*Linear regressions:*<br>***Model*** ***a0*** ***a1*** ***R^2***<br>***lin*** *1.3e5* *2.0e6* *0.266*<br>***constr.*** - *2.1e6* *0.265*<br><br>*Dyn. range:* 0.05 – 1.00 µg/mL
**BBP**<br>Benzyl butyl phthalate<br>CASRN: 85-68-7<br>MW: 312.14 g/mol	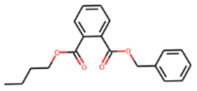	23.4	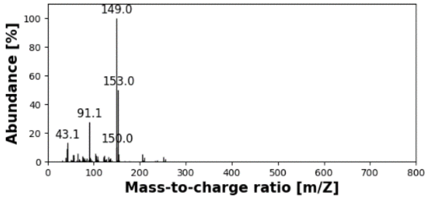<br>*coeluted with DHP	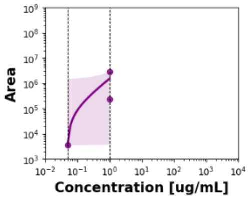	*Linear regressions:*<br>***Model*** ***a0*** ***a1*** ***R^2***<br>***lin*** *1.9e5* *3.1e6* *0.269*<br>***constr.*** - *3.3e6* *0.2674*<br><br>*Dyn. range:* 0.05 – 1.00 µg/mL

 https://doi.org/10.1021/acs.est.3c04851

Substance name	Structure	RT [min]	Massspectrum	Calibration curve
**DHP**<br>Dihexyl phthalate<br>CASRN: 84-75-3<br>MW: 334.21 g/mol	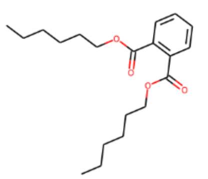	23.4	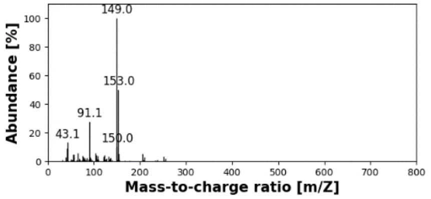<br>*coeluted with BBP	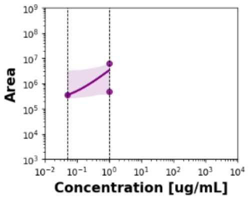<br>*Linear regressions:*<br>***Model*** ***a0*** ***a1*** ***R^2***<br>***lin*** 1.9e4 3.1e6 0.269<br>***constr.*** - 3.3e6 0.267<br>*Dyn. range:* 0.05 – 1.00 µg/mL
**DCHP**<br>Dicyclohexyl phthalate<br>CASRN: 84-61-7<br>MW: 330.18 g/mol	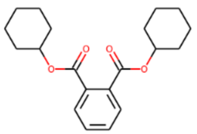	24.9	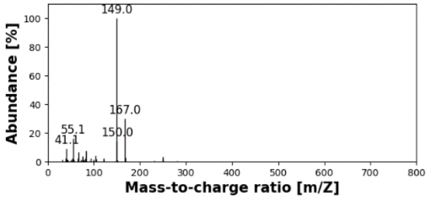	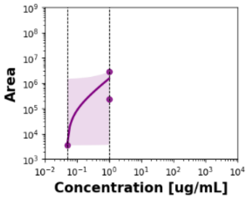<br>*Linear regressions:*<br>***Model*** ***a0*** ***a1*** ***R^2***<br>***lin*** -7.6e4 1.6e6 0.315<br>***constr.*** - 1.5e6 0.314<br>*Dyn. range:* 0.05 – 1.00 µg/mL
**DEHP**<br>Di(2-ethylhexyl) phthalate<br>CASRN: 117-81-7<br>MW: 390.28 g/mol	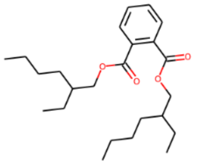	25.3	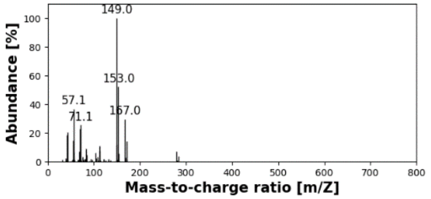	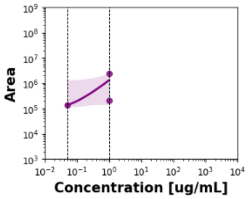<br>*Linear regressions:*<br>***Model*** ***a0*** ***a1*** ***R^2***<br>***lin*** 7.3e4 1.2e6 0.276<br>***constr.*** - 1.3e6 0.275<br>*Dyn. range:* 0.05 – 1.00 µg/mL
**DNOP**<br>Dioctyl phthalate<br>CASRN: 117-84-0<br>MW: 390.28 g/mol	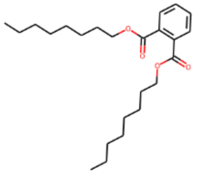	27.1	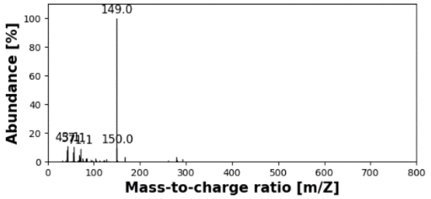	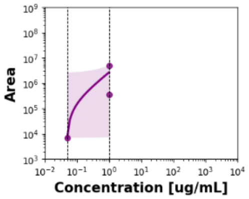<br>*Linear regressions:*<br>***Model*** ***a0*** ***a1*** ***R^2***<br>***lin*** -1.3e5 2.8e6 0.306<br>***constr.*** - 2.6e6 0.305<br>*Dyn. range:* 0.05 – 1.00 µg/mL
**DiNP**<br>Diisononyl phthalate<br>CASRN: 68515-48-0<br>MW: 418.31 g/mol	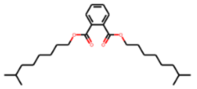<br>+ isomers	27.4	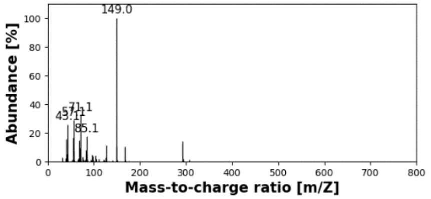	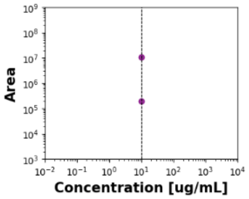<br>*Linear regressions:*<br>***Model*** ***a0*** ***a1*** ***R^2***<br>***lin*** *n.a.* *n.a.* *n.a*<br>***constr.*** - 5.6e5 *n.a.*<br>*Dyn. range:* >10 µg/mL
**DiDP**<br>Diisodecyl phthalate<br>CASRN: 68515-49-1<br>MW: 446.34 g/mol	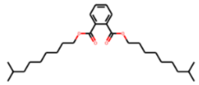<br>+ isomers	28.6	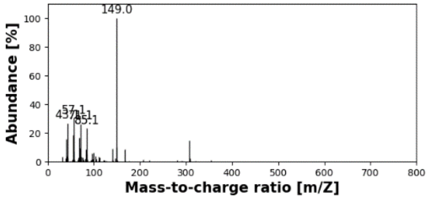	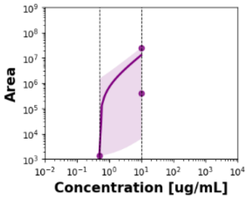<br>*Linear regressions:*<br>***Model*** ***a0*** ***a1*** ***R^2***<br>***lin*** -6.7e5 1.3e6 0.262<br>***constr.*** - 1.3e6 0.261<br>*Dyn. range:* 0.5 – 10.0 µg/mL

https://doi.org/10.1021/acs.est.3c04851

<table>
<tr><th>Substance name</th><th>Structure</th><th>RT [min]</th><th>Massspectrum</th><th>Calibration curve</th></tr>
<tr><td colspan="5">Alternative plasticizers</td></tr>
<tr><td>DEHA
Bis(2-ethylhexyl) adipate
CASRN: 103-23-1
MW: 370.31 g/mol</td><td>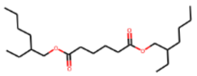</td><td>24.0</td><td>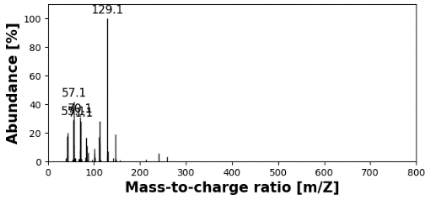
</td><td>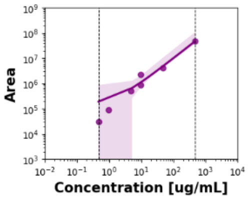

Linear regressions:
<table>
<tr><th>Model</th><th>a0</th><th>a1</th><th>R^2</th></tr>
<tr><td>lin</td><td>-1.4e5</td><td>1.0e5</td><td>0.999</td></tr>
<tr><td>constr.</td><td>-</td><td>1.0e5</td><td>0.999</td></tr>
</table>
Dyn. range: 0.5 - 471 µg/mL</td></tr>
<tr><td>DEHT
Bis(2-ethylhexyl) terephthalate
CASRN: 6422-86-2
MW: 390.28 g/mol</td><td>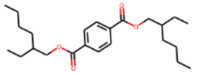</td><td>27.1</td><td>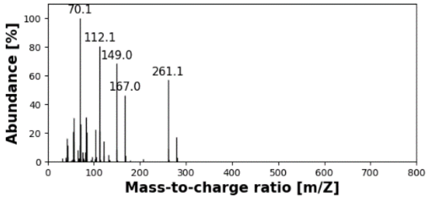
</td><td>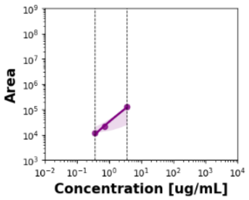

Linear regressions:
<table>
<tr><th>Model</th><th>a0</th><th>a1</th><th>R^2</th></tr>
<tr><td>lin</td><td>-2.3e3</td><td>3.5e4</td><td>0.999</td></tr>
<tr><td>constr.</td><td>-</td><td>3.4e4</td><td>0.998</td></tr>
</table>
Dyn. range: 0.4 - 4 µg/mL</td></tr>
<tr><td>DINCH
1,2-Cyclohexane dicarboxylic acid diisononyl ester
CASRN: 166412-78-8
MW: 424.36 g/mol</td><td>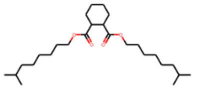</td><td>27.2</td><td>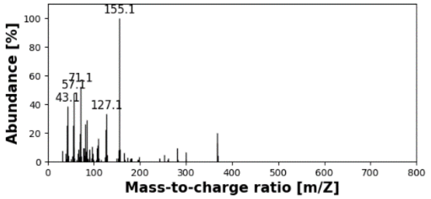
</td><td>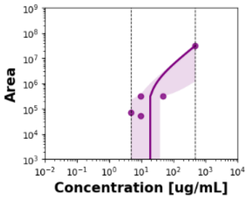

Linear regressions:
<table>
<tr><th>Model</th><th>a0</th><th>a1</th><th>R^2</th></tr>
<tr><td>lin</td><td>-9.7e5</td><td>6.7e4</td><td>0.994</td></tr>
<tr><td>constr.</td><td>-</td><td>6.5e4</td><td>0.989</td></tr>
</table>
Dyn. range: 5 - 477 µg/mL</td></tr>
</table>

https://doi.org/10.1021/acs.est.3c04851

Substance name	Structure	RT [min]	Massspectrum	Calibration curve	
Phosphate plasticiers / flame retardants					
**TBP**<br>Tributylphosphate<br>CASRN: 126-73-8<br>MW: 266.16 g/mol	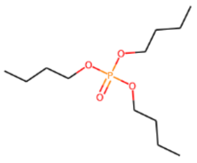	15.3	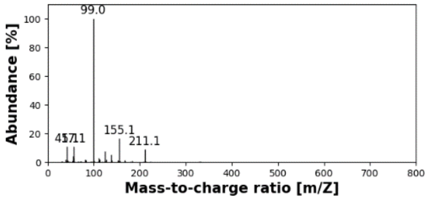	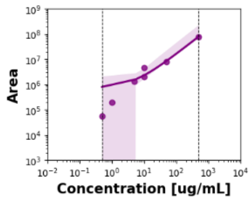	*Linear regressions:*<br>***Model*** ***a0*** ***a1*** ***R²***<br>***lin*** 7.2e5 1.6e5 0.999<br>***constr.*** - 1.6e5 0.998<br>Dyn. range: 0.5 - 500 µg/mL
**TCEP**<br>Tris-(2-chloroethyl) phosphate<br>CASRN: 115-96-8<br>MW: 283.95 g/mol	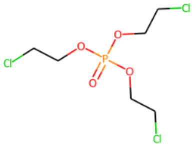	16.8	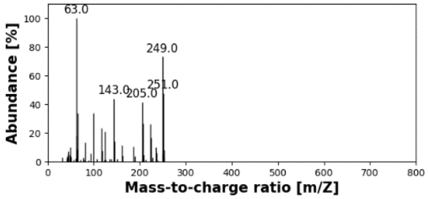	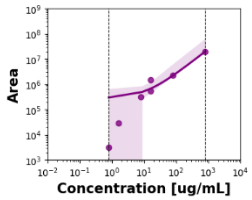	*Linear regressions:*<br>***Model*** ***a0*** ***a1*** ***R²***<br>***lin*** 2.8e5 2.4e4 0.997<br>***constr.*** - 2.5e4 0.996<br>Dyn. range: 1 - 805 µg/mL
**TCPP**<br>Tris-(2-chloroisopropyl) phosphate<br>CASRN: 13674-84-5<br>MW: 326.00 g/mol	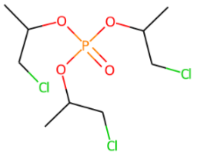	17.2	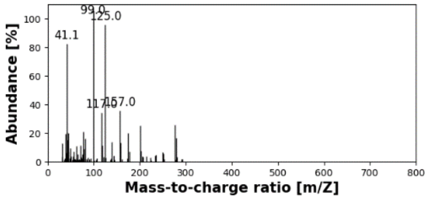	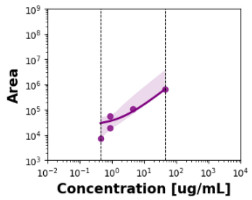	*Linear regressions:*<br>***Model*** ***a0*** ***a1*** ***R²***<br>***lin*** 2.3e4 1.4e4 0.994<br>***constr.*** - 1.4e4 0.987<br>Dyn. range: 0.4 - 44 µg/mL
**TDCPP**<br>Tris(1,3-dichloro-2-propyl)phosphate<br>CASRN: 13674-87-8<br>MW: 427.88 g/mol	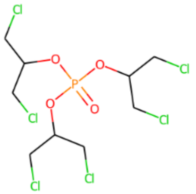	23.1	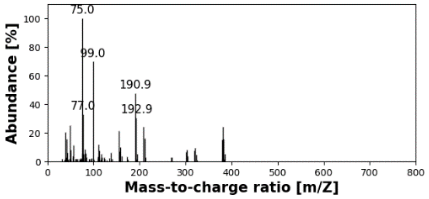	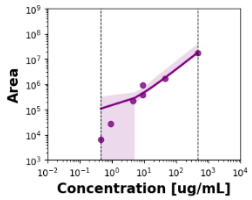	*Linear regressions:*<br>***Model*** ***a0*** ***a1*** ***R²***<br>***lin*** 9.0e4 3.8e4 0.999<br>***constr.*** - 3.8e4 0.999<br>Dyn. range: 0.5 - 460 µg/mL
**TPhP**<br>Triphenyl phosphate<br>CASRN: 115-86-6<br>MW: 326.07 g/mol	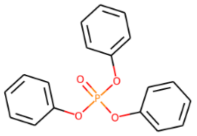	23.8	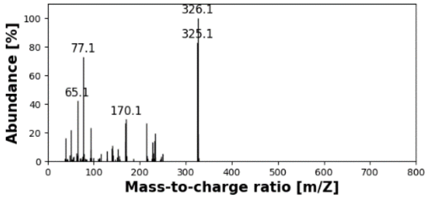	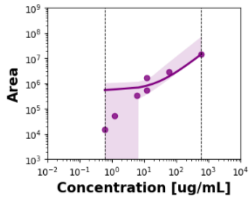	*Linear regressions:*<br>***Model*** ***a0*** ***a1*** ***R²***<br>***lin*** 5.3e5 2.3e4 0.986<br>***constr.*** - 2.4e4 0.976<br>Dyn. range: 0.6 - 607 µg/mL
**Octicizer**<br>2-Ethylhexyl diphenyl phosphate<br>CASRN: 1241-94-7<br>MW: 362.16 g/mol	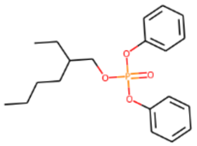	24.1	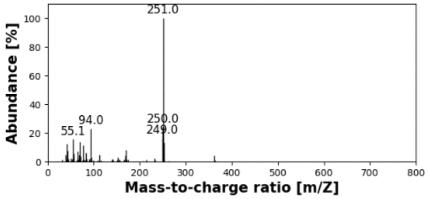	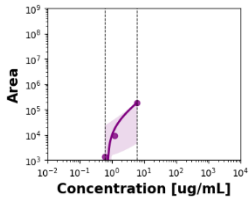	*Linear regressions:*<br>***Model*** ***a0*** ***a1*** ***R²***<br>***lin*** -2.6e4 3.5e4 0.996<br>***constr.*** - 3.0e4 0.953<br>Dyn. range: 0.6 - 6 µg/mL

https://doi.org/10.1021/acs.est.3c04851

Substance name	Structure	RT [min]	Massspectrum	Calibration curve	
**TEHP**<br>Tris(2-ethylhexyl) phosphate<br>CASRN: 78-42-2<br>MW: 434.35 g/mol	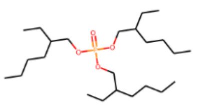	24.7	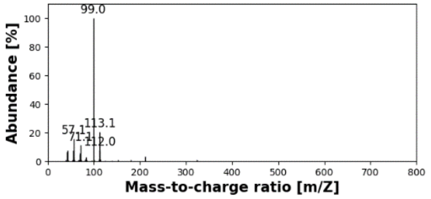	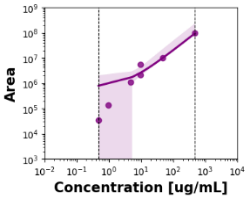	*Linear regressions:*<br>***Model*** ***a0*** ***a1*** ***R^2***<br>***lin*** 7.0e5 2.0e5 0.999<br>***constr.*** - 2.0e5 0.998<br>Dyn. range: 0.5 - 482 µg/mL
**TCP**<br>Tricresyl phosphate<br>CASRN: 1330-78-5<br>MW: 368.12 g/mol	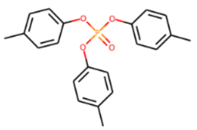	26.5	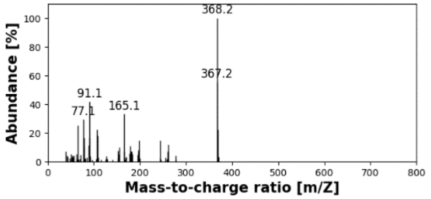	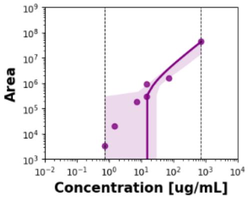	*Linear regressions:*<br>***Model*** ***a0*** ***a1*** ***R^2***<br>***lin*** -6.0e5 6.0e4 0.996<br>***constr.*** - 6.0e4 0.995<br>Dyn. range: 0.7 - 730 µg/mL
**TMPP**<br>Tri(3,4-dimethylphenyl)phosphate<br>CASRN: 3862-11-1<br>MW: 410.16 g/mol	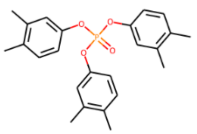	28.2	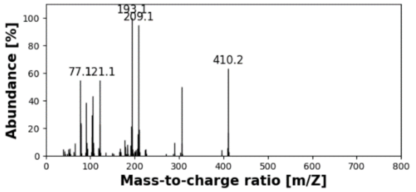	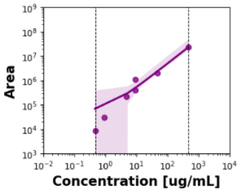	*Linear regressions:*<br>***Model*** ***a0*** ***a1*** ***R^2***<br>***lin*** 4.6e4 4.9e4 0.999<br>***constr.*** - 4.9e4 0.999<br>Dyn. range: 0.5 – 467 µg/mL
**TBPP**<br>Tris(2,3-dibromopropyl) phosphate<br>CASRN: 126-72-7<br>MW: 691.58 g/mol	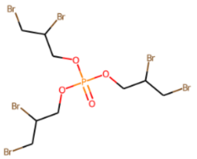	29.5	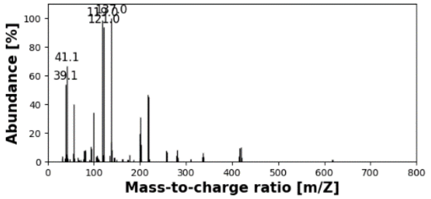	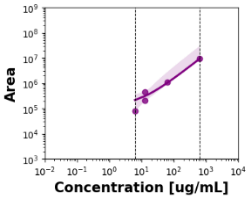	*Linear regressions:*<br>***Model*** ***a0*** ***a1*** ***R^2***<br>***lin*** 1.2e5 1.4e4 0.999<br>***constr.*** - 1.5e4 0.998<br>Dyn. range: 6 - 630 µg/mL
**TXP**<br>Tri(2,4-dimethylphenyl)phosphate<br>CASRN: 3862-12-2<br>MW: 410.16 g/mol	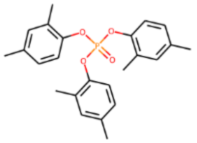	29.9	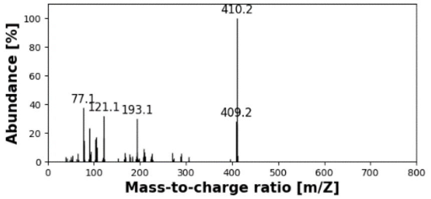	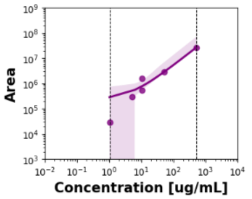	*Linear regressions:*<br>***Model*** ***a0*** ***a1*** ***R^2***<br>***lin*** 2.3e5 5.1e4 0.999<br>***constr.*** - 5.2e4 0.998<br>Dyn. range: 1.0 - 519 µg/mL

 https://doi.org/10.1021/acs.est.3c04851

Substance name	Structure	RT [min]	Massspectrum	Calibration curve	
Brominated flame retardants					
**TBPh**<br>2,4,6-tribromophenol<br>CASRN: 118-79-6<br>MW: 327.77 g/mol	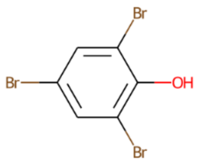	14.8	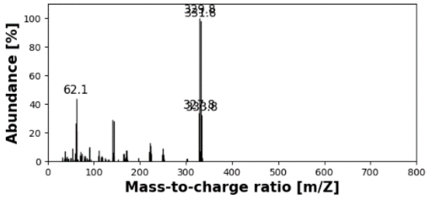	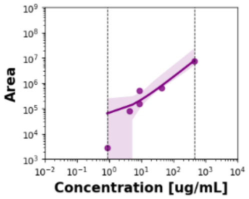	*Linear regressions:*<br>***Model*** ***a0*** ***a1*** ***R^2***<br>***lin*** 4.8e4 1.7e4 0.997<br>***constr.*** - 1.7e4 0.997<br>Dyn. range: 1 - 439 µg/mL
**BDE47**<br>2,2',4,4' -<br>Tetrabromodiphenyl ether<br>CASRN: 5436-43-1<br>MW: 481.72 g/mol	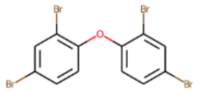	24.7	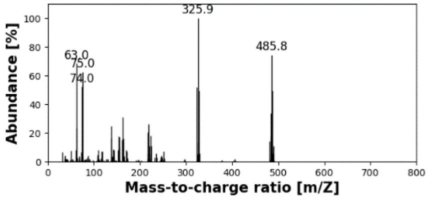	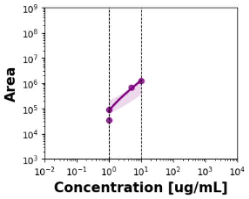	*Linear regressions:*<br>***Model*** ***a0*** ***a1*** ***R^2***<br>***lin*** -5.4e4 1.3e5 0.994<br>***constr.*** - 1.3e5 0.989<br>Dyn. range: 1.0 - 10 µg/mL
**TBBPA**<br>3,3',5,5'-<br>Tetrabromobisphénol A<br>CASRN: 79-94-7<br>MW: 539.76 g/mol	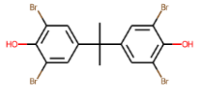	29.3	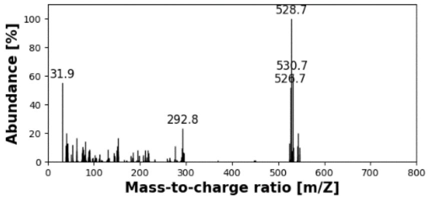	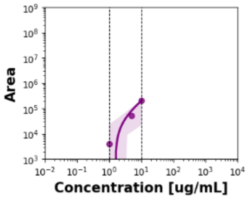	*Linear regressions:*<br>***Model*** ***a0*** ***a1*** ***R^2***<br>***lin*** -3.6e4 2.3e4 0.942<br>***constr.*** - 1.9e4 0.888<br>Dyn. range: 1.0 - 10 µg/mL
**gHBCD**<br>γ-1,2,5,6,9,10 –<br>Hexabromocyclododecane<br>CASRN: 134237-52-8<br>MW: 635.65 g/mol	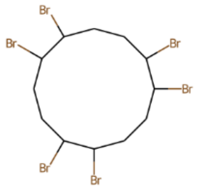	29.7	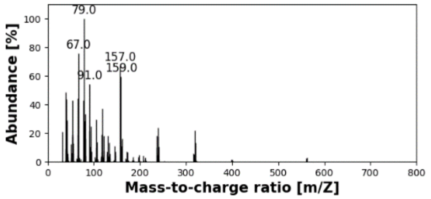	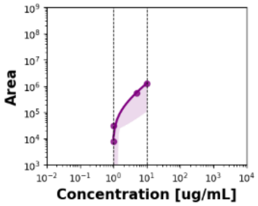	*Linear regressions:*<br>***Model*** ***a0*** ***a1*** ***R^2***<br>***lin*** -1.3e5 1.4e5 0.999<br>***constr.*** - 1.2e5 0.972<br>Dyn. range: 1.0 - 10 µg/mL
**BDE183**<br>2,2′,3,4,4′,5′,6-<br>Heptabromodiphenylether<br>CASRN: 207122-16-5<br>MW: 715.45 g/mol	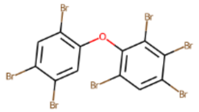	31.3	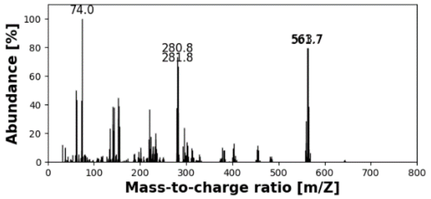	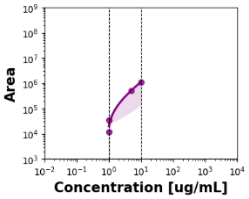	*Linear regressions:*<br>***Model*** ***a0*** ***a1*** ***R^2***<br>***lin*** -1.0e5 1.2e5 0.999<br>***constr.*** - 1.1e5 0.977<br>Dyn. range: 1.0 - 10 µg/mL
**BDE209**<br>Decabromodiphenylether<br>CASRN: 1163-19-5<br>MW: 959.17 g/mol	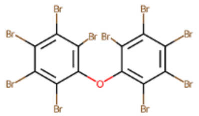	n.d.	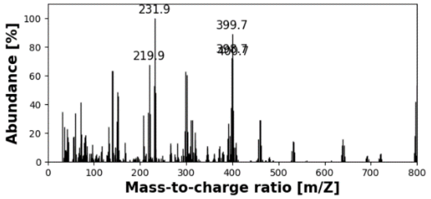		**Not available: BDE209 was available within a PVC reference material, which was prepared analogouly to the samples in this study.**

https://doi.org/10.1021/acs.est.3c04851

<table>
<tr><th>Substance name</th><th>Structure</th><th>RT [min]</th><th>Massspectrum</th><th>Calibration curve</th></tr>
<tr><td colspan="5">Antioxidants</td></tr>
<tr><td>dToc
δ-Tocopherol
CASRN: 119-13-1
MW: 402.35 g/mol</td><td>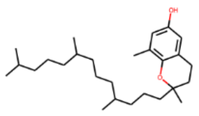</td><td>28.7</td><td>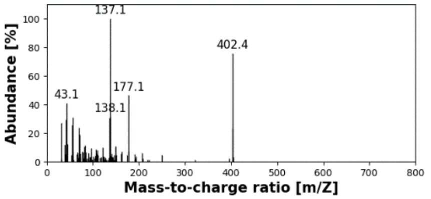</td><td>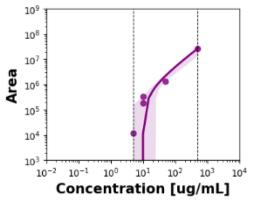
Linear regressions:
<table><tr><th>Model</th><th>a0</th><th>a1</th><th>R^2</th></tr><tr><td>lin</td><td>-5.4e5</td><td>5.5e4</td><td>0.999</td></tr><tr><td>constr.</td><td>-</td><td>5.3e4</td><td>0.996</td></tr></table>Dyn. range: 5 - 500 µg/mL</td></tr>
<tr><td>1035
Irganox 1035
CASRN: 41484-35-9
MW: 642.40 g/mol</td><td>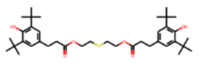</td><td>29.2</td><td>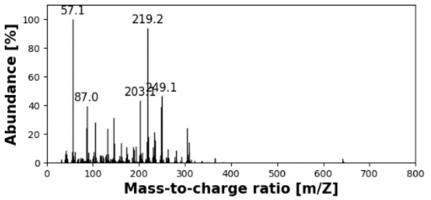</td><td>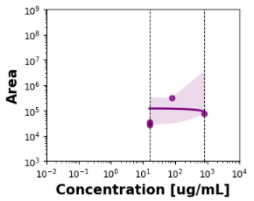
Linear regressions:
<table><tr><th>Model</th><th>a0</th><th>a1</th><th>R^2</th></tr><tr><td>lin</td><td>1.2e5</td><td>-3.6e1</td><td>0.010</td></tr><tr><td>constr.</td><td>-</td><td>1.3e2</td><td>-0.722</td></tr></table>Dyn. range: 16 – 796 µg/mL</td></tr>
<tr><td colspan="5">Bisphenols</td></tr>
<tr><td>BPA
Bisphenol-A
CASRN: 80-05-7
MW: 228.12 g/mol</td><td>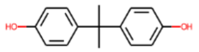</td><td>21.6</td><td>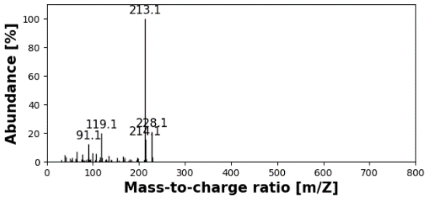</td><td>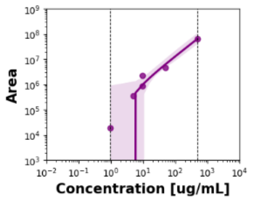
Linear regressions:
<table><tr><th>Model</th><th>a0</th><th>a1</th><th>R^2</th></tr><tr><td>lin</td><td>-3e5</td><td>1.3e5</td><td>0.999</td></tr><tr><td>constr.</td><td>-</td><td>1.3e5</td><td>0.999</td></tr></table>Dyn. range: 1 – 488 µg/mL</td></tr>
<tr><td>BPS
Bisphenol-S
CASRN: 80-09-1
MW: 250.03 g/mol</td><td>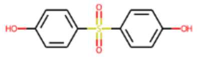</td><td>27.2</td><td>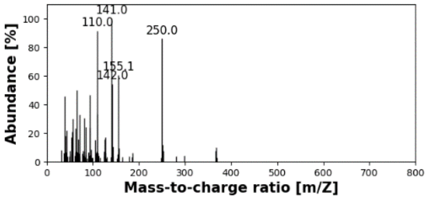</td><td>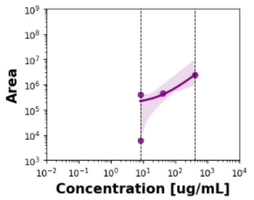
Linear regressions:
<table><tr><th>Model</th><th>a0</th><th>a1</th><th>R^2</th></tr><tr><td>lin</td><td>1.7e5</td><td>5.5e3</td><td>0.978</td></tr><tr><td>constr.</td><td>-</td><td>6.0e3</td><td>0.955</td></tr></table>Dyn. range: 8 - 413 µg/mL</td></tr>
</table>

https://doi.org/10.1021/acs.est.3c04851

S2.4 Details Bioassays

Sample preparation. The same extraction procedure as above was used (section S2.3.3) except that samples were not diluted after filtration but concentrated, since most bioassays have a low solvent tolerance (MTT/ROS: max 0.1 volume%). Using a Syncore system from Buchi to avoid losses of volatile substances, the solvent from 12 samples was evaporated in parallel, from approximately 6mL to 300 µL (pressure: 210mbar, temperature top of the flasks: 60°C, temperature bottom of the flask: 10°C). These samples were stored at –20°C. However, during the inter-laboratory shipping (2–3 days), the temperature may have risen to 20°C. Due to the high volatility of *THF*, the sample volumes decreased during the storage and transport. Before applying to each assay, samples were taken out from –20°C, the volume of each sample was inspected and, if necessary, filled up to 300 µl with THF. Then, samples were stored overnight at 4°C prior to the testing.

Extract selection: The samples screened for cytotoxicity (MTT assay) and reactive oxygen species generation (ROS assay) were selected at random (n=85). The selected samples can be seen in Sheet S1 and Sheet S10 in SI2. The samples for the endocrine activity assays, AMES test and planar-umuC bioassay were selected as to be maximally different regarding their *ortho*-phthalate content and their activity in the MTT assay (Table S11).

Table S11: Selected extracts for further screening with YES/YAS, umuC and AMES bioassays based on MTT viability and *ortho*-phthalate content.

Sample_id	MTT viability [%]	o-phthalate content [wt%]	YES/YAS	planar-umuC	Ames
g5	53.76	12.08		x	
d80-2	57.21	16.73		x	x
d1-2	49.82	0	x	x	
d31-1	50.69	0	x		
d1-1	57.74	0		x	
g4	70.81	33.02	x	x	
g1	82	20.61	x		
d21-1	88.03	47.14		x	x
d20-2	97.84	40.13	x	x	
gar1	105.99	40.35	x		
g2	125.51	18.19	x		
d20-1	108.43	35.02		x	x
g3	99.72	18.5		x	
d42-2	92.99	0	x		
d13-2	93.29	0		x	x
g7	128.66	0.01		x	x
d75-2	110.25	0		x	

Cytotoxicity and oxidative stress. Randomly selected extracts (n=85) were screened for cytotoxicity using MTT assays and for oxidative stress using ROS assays. Both assays were conducted on human liver cells (Huh7), according to Christen et al. 2014.[17] Cells were grown in DMEM with GlutaMAX™ (LuBioScience, Lucerne, Switzerland) supplemented with 10% FBS (Sigma-Aldrich, Taufkirchen, Germany) in a humidified incubator with 5% CO_2 at 37 °C. Cells were usually split every 4 days and sub-cultured at split ratios of about 1:6. Then, Huh7 cells were plated at a density of 25 000 cells per well in 96-well plates. After 24 h, cells were treated either with the highest possible test concentration (1 µl extract/1 ml cell culture medium, as solvent concentration should not exceed 0.1 volume%), or for selected ones, with a serial dilution of the extracts (1:2 dilution steps). The samples were classified based on the cell viability in the MTT assay: "highly toxic" for below 30%, "moderately toxic" for 30–60%, "slightly toxic" for 60–90%, and "not toxic" for above 90%.

Endocrine activity. Eight selected extracts were screened for estrogenic, anti-estrogenic, androgenic, and anti-androgenic activities using XenoScreen YES/YAS assays from Xenometrix (Allschwil, Switzerland). Serial dilutions of selected extracts (highest test concentration: 1:150 dilution of pure extract) were tested according to the manufacturer's protocol.

Mutagenicity. Nine selected extracts were analyzed for potential mutagenic activity using Ames MPF 98/100 from Xenometrix (Allschwil, Switzerland) with *Salmonella typhimurium* strains TA98 (for detection of frameshift mutations) and TA100 (for detection of base substitution mutations), in accordance with the manufacturer's protocol.

Genotoxicity. Twelve selected extracts were analyzed for potential direct genotoxic activity using the planar-umuC bioassay protocol of planar4 GmbH (Stäfa, Switzerland). The planar-umuC was conducted on normal phase, silica gel Si 60 HPTLC plates (Merck, Germany), with the *Salmonella typhimurium* strain TA1535 pSK1002 (Xenometrix, Allschwil, Switzerland). The raw samples (300 µl) were first diluted to 800 µl ACN/THF to facilitate handling. All samples were then diluted 1:10, 1:100 and 1:1000, and applied to the HPTLC plates using an Automatic TLC samples (ATS4, Camag, Switzerland). A solvent blank (ACN/THF, for sample dilution), a second solvent blank (solvent of positive control) and three 4-NQO positive controls with a mass per band of 100, 200 and 800 pg were also applied. The HPTLC plates were developed with ACN:DCM (dichloromethane) (1:1) from 20 mm to 75 mm. A total of 8 runs were conducted. The genotoxicity

after metabolic activation was not determined because the respective planar-umuC protocol was not available at the time of the experiment.

QA/QC. Procedural and solvent blank samples were tested to ensure effects were caused by substances present in the samples. MTT and ROS screening were performed in triplicate, whereas the other assays were repeated as often as recommended by the respective protocols.

S2.5 Data treatment

Data treatment included (1) treatment using specialized software for the analysis method (e.g. NITON plastics calibration for XRF, Agilent Masshunter for GC-MS), which is described in the respective sections, and (2) further combined data analysis, which was conducted in python (relevant scripts are provided in SI5). Further data treatment included combining and aligning data, creating the graphs for this paper, conducting principal component analysis, and clustering the data.

Furthermore, the raw data produced in this campaign is provided for further analysis in the following formats in the SI4: (1) ATR-FTIR spectra for each sample and side as '.csv', (2) XRF based elemental concentrations for each sample and side as 'excel', (3) SIM GC-MS spectra for phthalate measurements of each sample as 'Agilent' and 'mzXML', and calculated concentrations as 'excel', (4) SCAN GC-MS spectra for suspect screening of each sample as 'Agilent' and 'mzXML', and detection, identification and semi-quantification results as 'excel', (5) bioassay readings as 'excel'.

S3 RESULTS

S3.1 Concentrations and presence of individual substances

Table S12: Concentration and presence of individual elements based on XRF elemental analysis. Summary statistics (minimum, median, mean, sd, und maximum) are shown for the detected fraction only. The limits of detection (LODs) were calculated according to the instrument manufacturer's protocol, as three times the minimum standard deviation of the analyte. The table is sorted based on the detection frequency, if not detected by the abbreviation of the element. Abbr. = Abbreviation, CASRN = Chemical Abstract Service Registry Number, DF=Detection frequency, LOD = Limit of detection, SD = Standard deviation.

Name	Abbr.	DF [%]	LOD [mg/kg]	Min. [mg/kg]	Median [mg/kg]	Mean [mg/kg]	SD [mg/kg]	Max. [mg/kg]
Regulated Elements (i.e.Cr, Pb, Hg, Cd, As)	-	*19.2*	-	*12*	*72*	*1'457*	*3'519*	*15'619*
Zink	Zn	96.0	17	42	289	636	1'032	9'583
Iron	Fe	76.2	33	90	608	735	594	3'613
Barium	Ba	72.2	67	74	425	466	249	1'173
Titanium	Ti	67.5	24	55	1'629	5'091	9'210	50'342
Tin	Sn	58.3	18	26	123	238	877	8'260
Vanadium	V	45.7	10	31	71	134	147	674
Bromine	Br	22.5	4	9	24	33	24	91
Antimony	Sb	11.3	24	32	160	2'335	4'833	17'938
Chromium	Cr	9.3	11	36	69	169	326	1'289
Lead	Pb	8.6	6	15	144	3'025	4'534	14'330
Bismuth	Bi	4.6	9	17	25	24	5	30
Copper	Cu	4.0	18	47	99	89	29	121
Nickel	Ni	4.0	14	57	75	75	13	90
Arsenic	As	2.0	5	12	30	41	36	81
Gold	Au	-	17	-	-	-	-	-
Cadmium	Cd	-	13	-	-	-	-	-
Mercury	Hg	-	12	-	-	-	-	-
Selenium	Se	-	7	-	-	-	-	-

Table S 13: Concentration and presence of individual *ortho*-phthalates based on the phthalate GC-MS quantification workflow. Summary statistics (minimum, median, mean, sd, und maximum) are shown for the detected fraction only. The table is sorted based on the detection frequency, if not detected by the retention time of the standard. Abbr. = Abbreviation, CASRN = Chemical Abstract Service Registry Number, DF=Detection frequency, LOD = Limit of detection, SD = Standard deviation.

**Name**	**Abbr.**	**CASRN**	**DF** [%]	**LOQ** [mg/kg]	**Min.** [mg/kg]	**Median** [mg/kg]	**Mean** [mg/kg]	**SD** [mg/kg]	**Max.** [mg/kg]
Any ortho-phthalate		-	*36.4*	-	*32*	*37’752*	*86’852*	*120’588*	*471’434*
Restricted ortho-phthalate (i.e. DEHP, BBP, DiBP, DBP)		-	*20.5*	-	*32*	*4’018*	*16’303*	*40’155*	*205’086*
Diisononyl phthalate	DiNP	68515-48-0	23.8	504	595	34’518	89’370	124’083	458’472
Di(2-ethylhexyl) phthalate	DEHP	117-81-7	18.5	32	32	3’574	17’494	41’974	204’728
Diisodecyl phthalate	DiDP	68515-49-1	15.9	360	526	8’465	27’220	57’203	283’597
Diethyl phthalate	DEP	84-66-2	7.3	50	119	2’386	1’941	987	2’824
Diisobutyl phthalate	DiBP	84-69-5	5.3	36	39	154	979	1’448	4’122
Isopentylpentyl phthalate	nPiPP	776297-69-9	2.6	50	54	102	98	39	135
Benzyl butyl phthalate	BBP	85-68-7	2.6	36	95	313	990	1’505	3’242
Dioctyl phthalate	DNOP	117-84-0	2.6	43	54	83’400	88’277	102’099	186’255
Dicyclohexyl phthalate	DCHP	84-61-7	2.0	43	3’584	8’736	8’612	4’968	13’518
Di-n-butylphthalate	DBP	84-74-2	1.3	36	42	1’891	1’891	2’616	3’741
Dimethyl phthalate	DMP	131-11-3	0.7	50	79	79	79	-	79
Diisopentyl phthalate	DiPP	605-50-5	0.7	43	56	56	56	-	56
Diallyl phthalate	DAP	131-17-9	-	50	-	-	-	-	-
Bis(-2-methoxyethyl) phthalate	DMEP	117-82-8	-	36	-	-	-	-	-
Di-n-pentyl phthalate	DPP	131-18-0	-	36	-	-	-	-	-
Dihexyl phthalate	DHP	84-75-3	-	43	-	-	-	-	-

Table S14: Concentration and presence of individual substances based on GC-MS suspect screening workflow. The LODs reported here are based on a dilution series and only give an approximate measure for the limit of detection. Concentration estimates are based on semi-quantification, and may be above 1'000'000 mg/kg for samples outside the calibration range. Summary statistics (minimum, median, mean, sd, and maximum) are shown for the detected fraction only. The table is sorted based on the substance group and the detection frequency, if not detected by the retention time of the standard. Abbr. = Abbreviation, CASRN = Chemical Abstract Service Registry Number, DF=Detection frequency, LOD = Limit of detection, SD = Standard deviation.

Name	Abbr.	CASRN	DF *[%]*	LOD [mg/kg]	Min. [mg/kg]	Median [mg/kg]	Mean [mg/kg]	SD [mg/kg]	Max. [mg/kg]
Alternative plasticizers									
Bis(2-ethylhexyl) terephthalate	DEHT	6422-86-2	56.3	288	5'087	462'905	608'684	717'140	3'678'195
Bis(2-ethylhexyl) adipate	DEHA	103-23-1	19.2	360	81	7'182	48'951	131'853	522'876
Tris(2-ethylhexyl) trimellitate	TEHTM	3319-31-1	4.0	n.a.	-	-	-	-	-
1,2-Cyclohexane dicarboxylic acid diisononyl ester	DINCH	166412-78-8	3.3	3'600	237'129	1'103'038	1'050'023	761'139	2'076'224
Bis(2-ethylhexyl) isophthalate	DEHI	137-89-3	0.7	n.a.	-	-	-	-	-
Phosphate plasticizers / flame retardants									
2-Ethylhexyl diphenyl phosphate	Octicizer	1241-94-7	13.2	432	327	7'779	47'549	170'564	770'869
Triphenyl phosphate	TPhP	115-86-6	7.3	432	861	2'108	4'125	6'961	24'802
Tributylphosphate	TBP	126-73-8	-	360	-	-	-	-	-
Tris-(2-chloroethyl) phosphate	TCEP	115-96-8	-	720	-	-	-	-	-
Tris-(2-chloroisopropyl) phosphate	TCPP	13674-84-5	-	288	-	-	-	-	-
Tricresyl phosphate	TCP	1330-78-5	-	504	-	-	-	-	-
Tri(3,4-dimethylphenyl)phosphate	TMPP	3862-11-1	-	360	-	-	-	-	-
Tri(2,4-dimethylphenyl)phosphate	TXP	3862-12-2	-	720	-	-	-	-	-
Tris(1,3-dichloro-2-propyl)phosphate	TDCPP	13674-87-8	-	360	-	-	-	-	-
Tris(2-ethylhexyl) phosphate	TEHP	78-42-2	-	360	-	-	-	-	-
Tris(2,3-dibromopropyl) phophate	TBPP	126-72-7	-	4'320	-	-	-	-	-
Brominated flame retardants									
2,4,6-tribromophenol	TBPh	118-79-6	-	720	-	-	-	-	-
2,2',4,4'-Tetrabromodiphenyl ether	BDE47	5436-43-1	-	720	-	-	-	-	-
3,3',5,5'-Tetrabromobisphénol A	TBBPA	79-94-7	-	720	-	-	-	-	-
γ-1,2,5,6,9,10 - Hexabromocyclododecane	gHBCD	134237-52-8	-	720	-	-	-	-	-
2,2′,3,4,4′,5′,6-Heptabromdiphenylether	BDE183	207122-16-5	-	720	-	-	-	-	-
Decabromodiphenylether	BDE209	1163-19-5	-	n.a.	-	-	-	-	-
Antioxidants / UV Stabilizers									
Bumetrizol	UV-326	3896-11-5	4.0	n.a.	-	-	-	-	-
δ-Tocopherol	dToc	119-13-1	-	3'600	-	-	-	-	-
Irganox 1035	1035	41484-35-9	-	11'520	-	-	-	-	-
Bisphenols									
Bisphenol-A	BPA	80-05-7	1.3	720	2'820	3'325	3'325	713	3'829
Bisphenol-S	BPS	80-09-1	-	5'760	-	-	-	-	-

Aside from our suspect list, also other compounds were discovered by the library identification. All discovered substances, their corresponding samples and a prioritization of the substances (based on total area and number of relevant samples) is presented on Sheet S9 in SI2. Their chemical space plot can be seen in Figure S 7, weighted by the peak area, and .Figure S 8, weighted by the detection frequency.

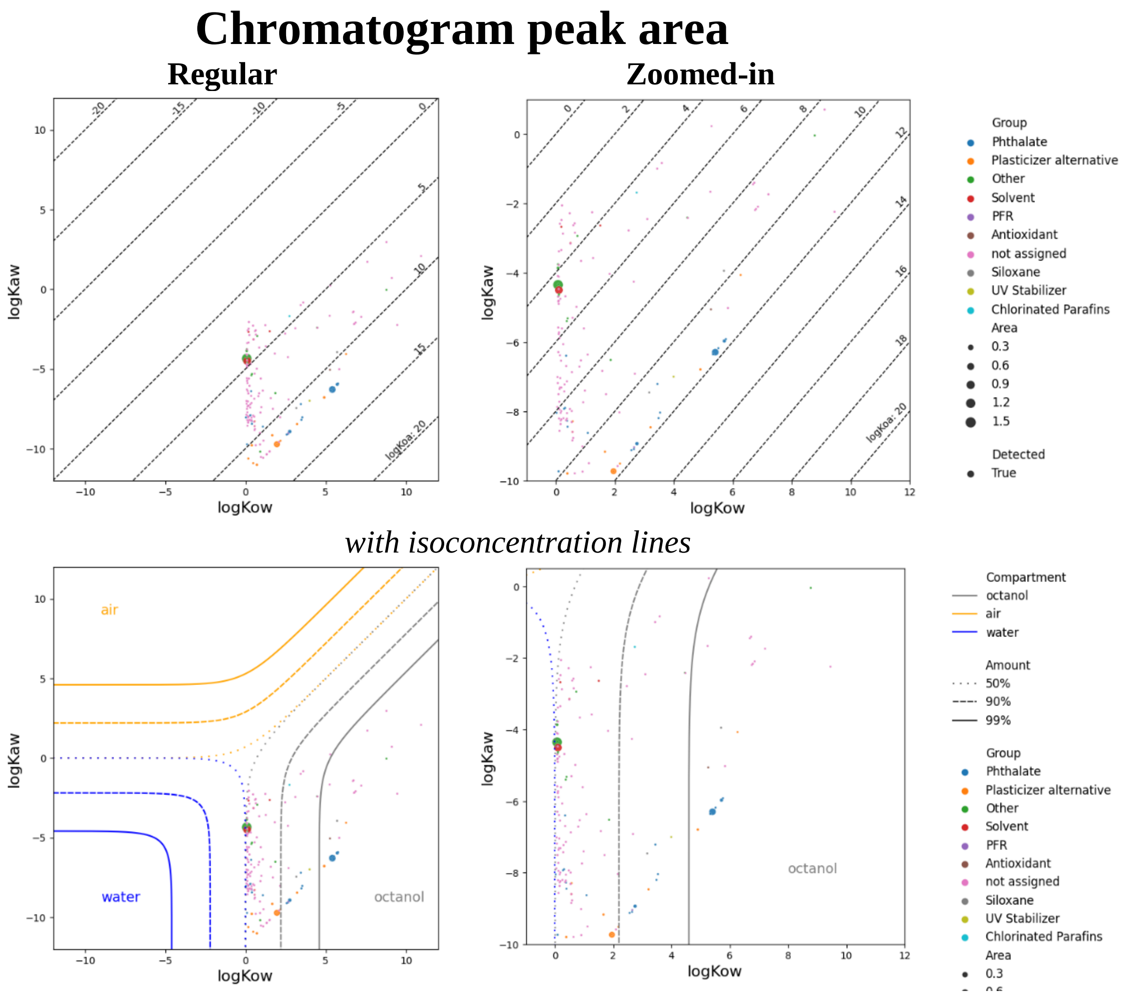

Figure S 7: Chemical space of the substances detected in suspect list screening, marker size scaled to their total chromatogram area. For the bottom plots, the iso-concentration curves are calculated for equal volumes of each compartment (i.e. water, air and octanol are exactly the same volume). Most additional substances that were not standards have a logKow around zero, meaning they are dynamic and easily leach from octanol-like environments.

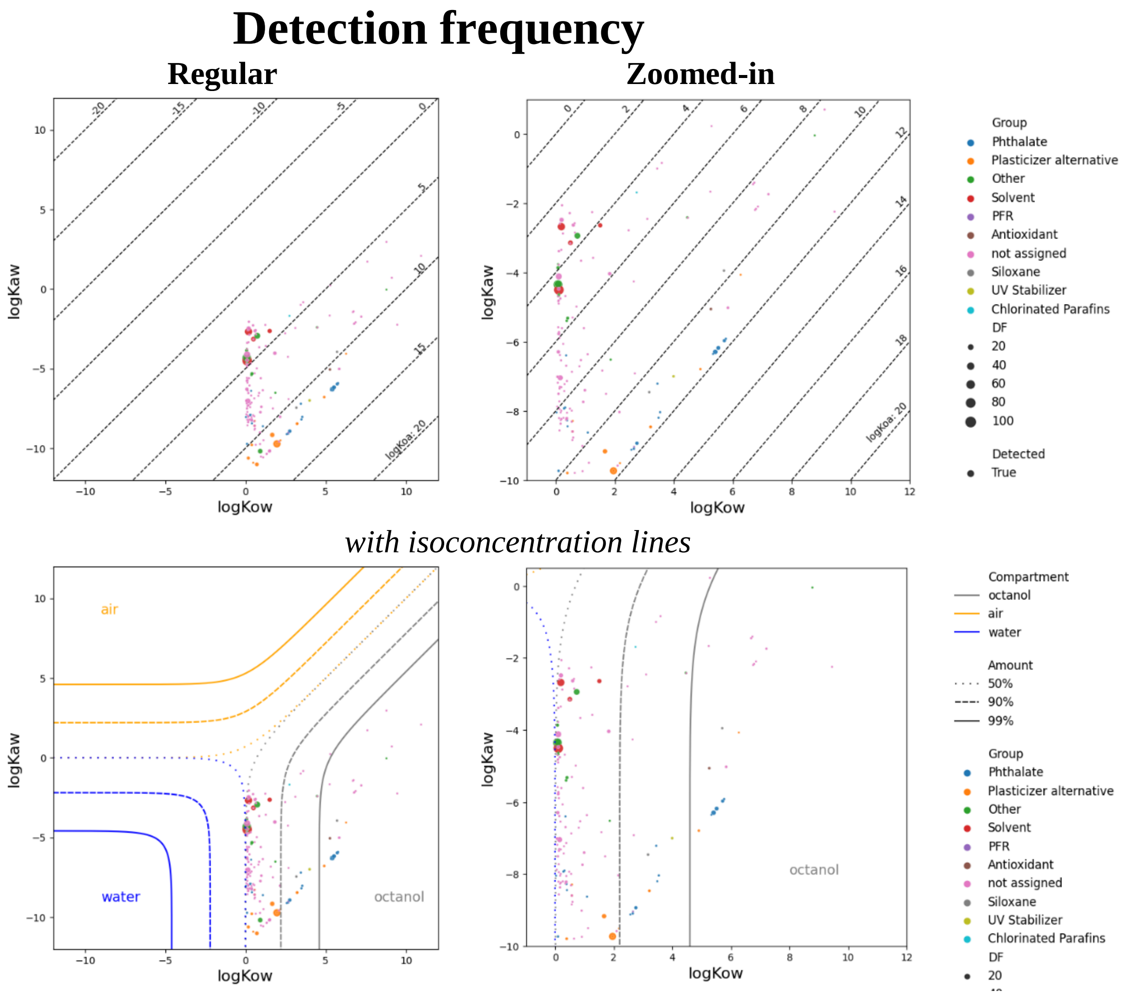

Figure S 8: Chemical space of the substances detected in suspect list screening, marker size scaled to their detection frequency. For the bottom plots, the iso-concentration curves are calculated for equal volumes of each compartment (i.e. water, air and octanol are exactly the same volume). Most additional substances that were not standards have a logKow around zero, meaning they are dynamic and easily leach from octanol-like environments.

S3.2 Total plasticizer content

Approximate plasticizer composition and total amount per sample are displayed in Figure S9, individual values for each sample can be found in Sheet S1 in SI2. The values for semi-quantified substances are highly uncertain as many were outside the respective calibration curve range.

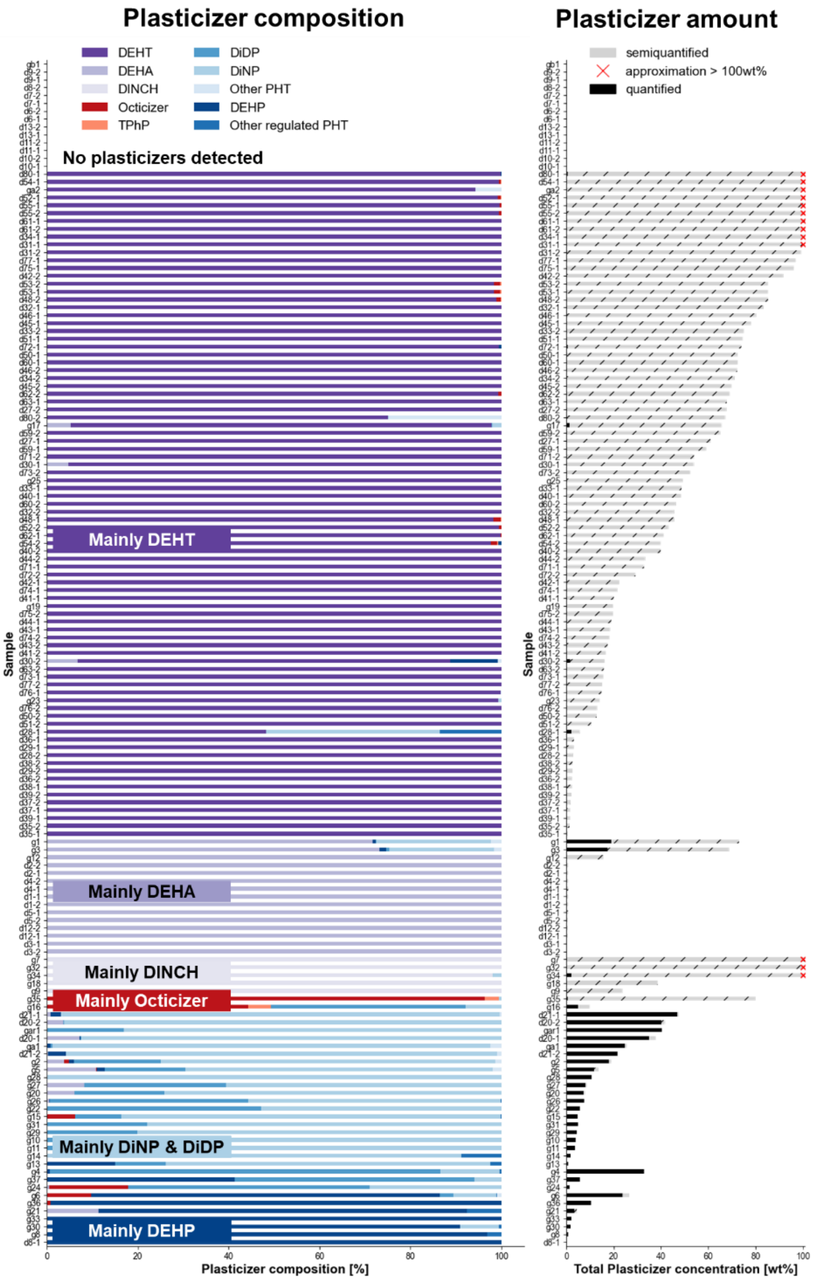

Figure S9: Plasticizer composition (left) and amount (right) by sample. Sorted by the major plasticizer per sample. Semi quantification of some plasticizer resulted in very high concentration estimates (some concentrations are even above 100wt%), which is mainly due to calibration curve uncertainty especially for signals above the calibration curve range.

S3.3 Correlation between Substances

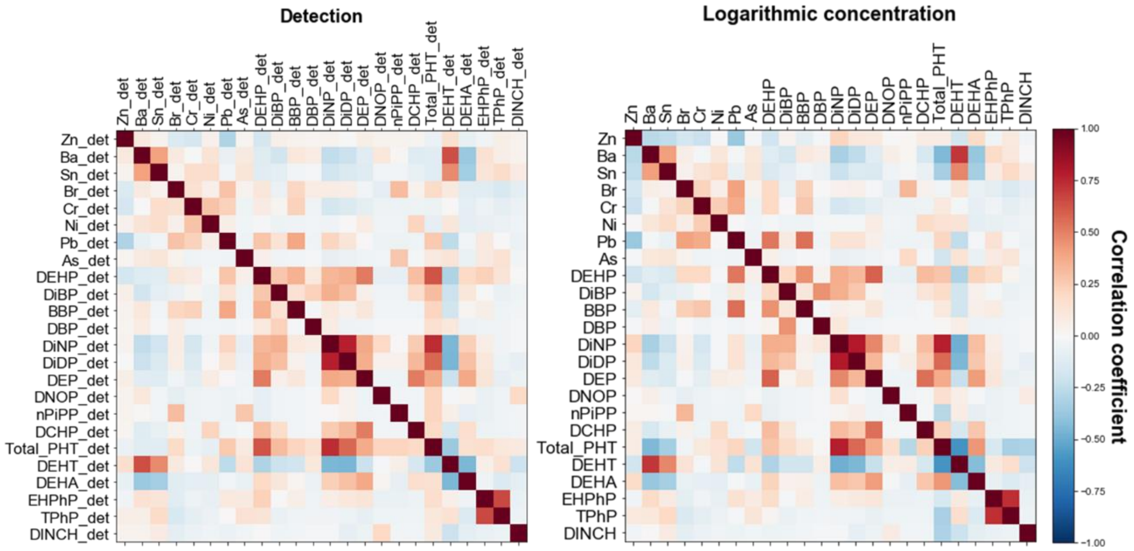

Figure S 10: Correlation matrix for detection and logarithmic concentration of all measured samples

S3.4 Bioassay results

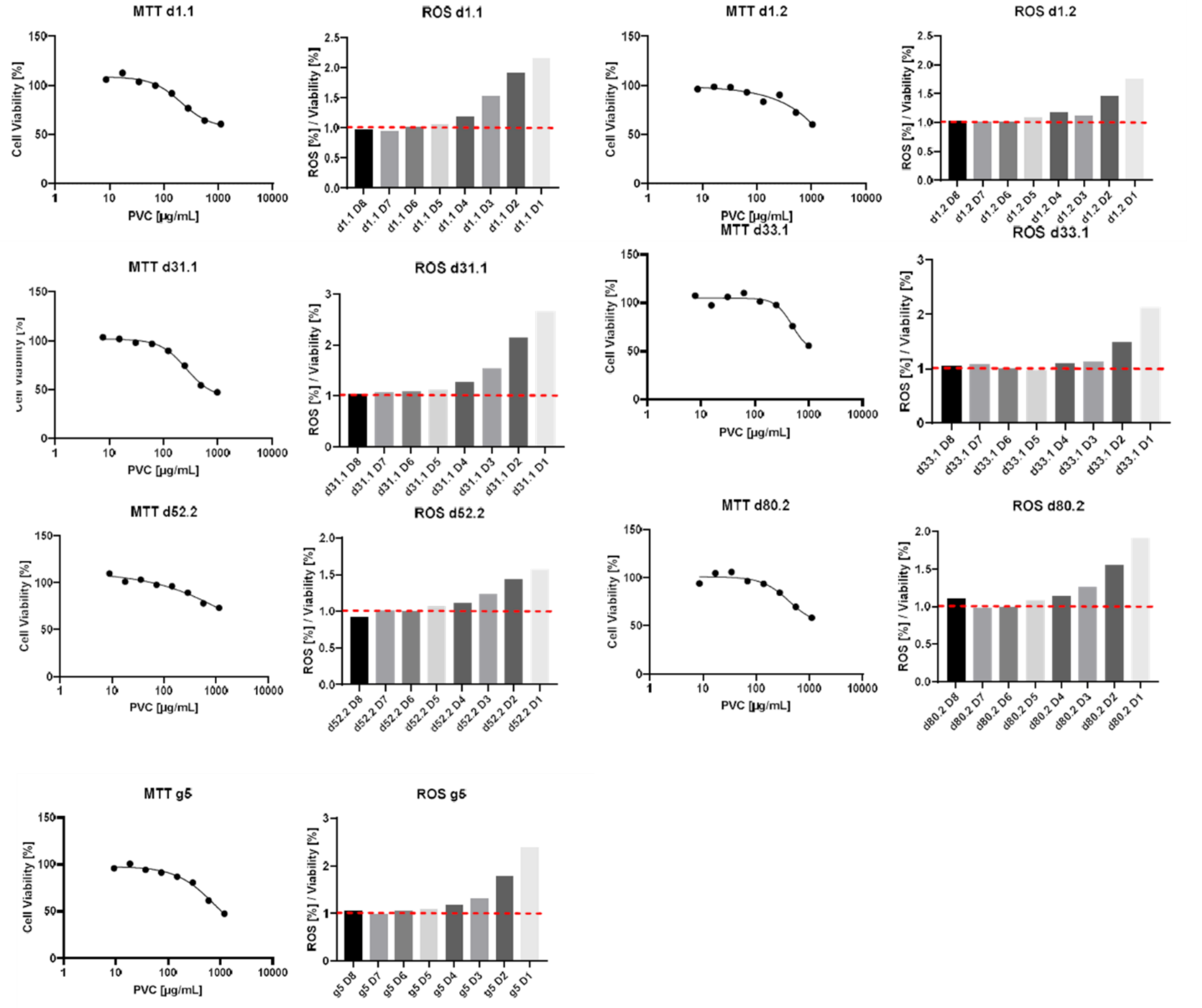

Figure S 11: Cell viability and induction of oxidative stress in Huh7 cells after exposure to plastic extracts. Huh7 cells were exposed to a serial dilution with a dilution-factor of 2 (d1: highest concentration, d8: lowest concentration) of the seven plastic extracts which induced more than 40% of cell mortality in the first screen.

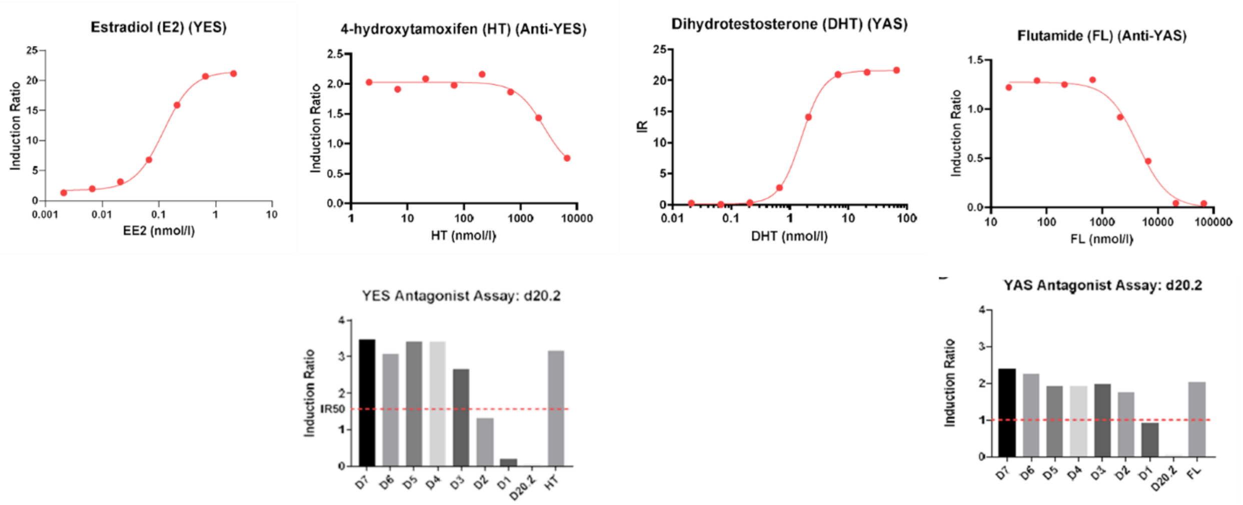

Figure S 12: Endocrine activity of selected plastic extracts. Estrogenic, anti-estrogenic, androgenic, and anti-androgenic activities were analysed in yeast cells after exposure to selected plastic extracts. Shown are the estrogenic, anti-estrogenic, androgenic, and anti-androgenic controls of the kit and data of extract d20.2. A serial dilution with a dilution factor of 2 from the highest possible concentration (d20.2) to the lowest test concentration (d7) was analysed. Red dotted lines point to the expected hormonal activities.

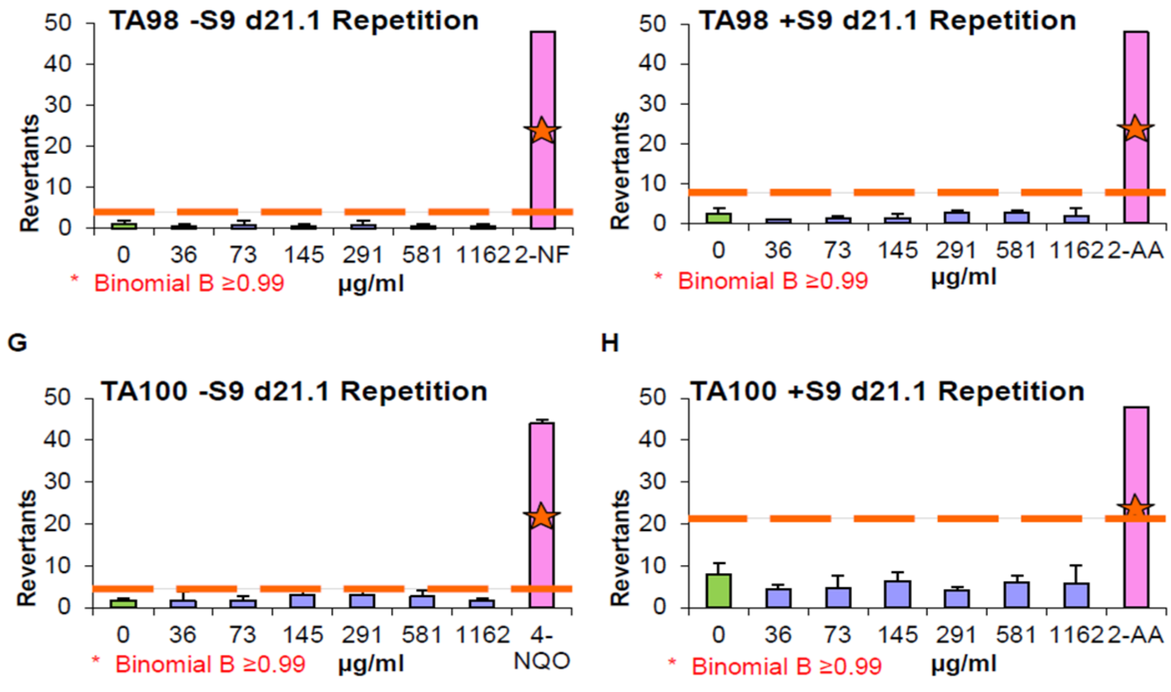

Figure S 13: Induction of mutagenicity by extract d21.1. ***Salmonella typhimurium*** **strains TA98 and TA100, each with and without S9 liver fractions, were exposed to a serial dilution of extract d21.1 and controls for 48h. Positive controls: 2-nitrofluorene (2-NF), 2-aminoanthracene (2-AA), and 4-nitroquinoline (4-NQO). Data is presented as number of reverted mutations per concentration with standard deviation from one experiment. Red line: 2-fold increase over baseline. Red star: binominal B≥ 0.99.**

 https://doi.org/10.1021/acs.est.3c04851

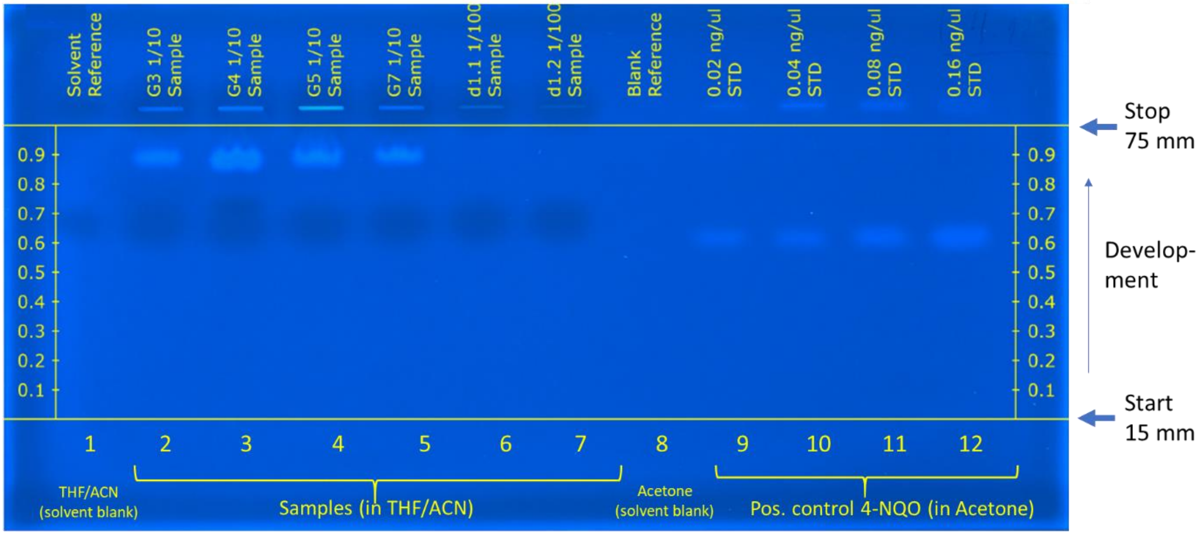

Figure S 14 Genotoxicity of selected samples, measured with the planar-umuC bioassay (samples g3, g4, g5, g7, d1.1 and d1.2 (tracks 2 to 7, all at Rf 0.9). The dark bands of tracks 1-7 at Rf 0.7 indicate an inhibition of the planar-umuC test system by the THF-ACN (1:3) solvent. Control tracks of solvent in (track 8). Positive control 4-NQO (tracks 9-12) in increasing concentration.

S3.5 Linear regression models

S3.5.1 Toxic metals – presence and concentration

Table S 15: Linear regression model for predicting the chance of any toxic metal(loids), i.e. Cd, Pb, Cr, Ni, Hg, As, being present (in %) based on sample properties (independent variables).

**Chance of presence of any toxic metals [%]**	**Coefficient**	**std err**	**t-value**	**p-value**	**[0.025**	**0.975]**
**Constant**	43.3	10.7	4.057	0.000**	22.2	64.5
**Independent Variables**						
Originating from DIY store	-11.3	10.1	-1.122	0.264	-31.2	8.6
Presence of grey layer	13.9	10.5	1.329	0.186	-6.8	34.6
Hardness	-11.5	6.4	-1.804	0.073*	-24.2	1.1
Number of layers	-2.6	3.9	-0.664	0.508	-10.4	5.2
Color of top layer:						
beige, orange or brown	9.0	9.5	0.953	0.342	-9.7	27.8
black	9.9	9.3	1.064	0.289	-8.5	28.2
blue or green	5.6	12.4	0.449	0.654	-18.9	30.1
grey	5.5	8.8	0.626	0.533	-12.0	23.0
red	0.1	15.7	0.006	0.996	-30.9	31.1
transparent or white	-1.8	9.2	-0.191	0.849	-20.0	16.5
wood	15.0	8.2	1.842	0.068*	-1.1	31.2

*** significant contributions ($p < 0.05$)*
** possibly significant contributions ($p < 0.10$)*

Table S 16: Linear regression model for predicting the concentration in ppm of toxic metal(loids), i.e. Cd, Pb, Cr, Ni, Hg, As, based on sample properties (independent variables)

**Concentration of toxic metals [ppm]**	**Coefficient**	**std err**	**t-value**	**p-value**	**[0.025**	**0.975]**
**Constant**	1490	410	3.60	0.000**	670	2310
**Independent Variables**						
Originating from DIY store	-440	390	-1.14	0.257	-1220	330
Presence of grey layer	1490	410	3.67	0.000**	690	2280
Hardness	-380	250	-1.52	0.131	-870	110
Number of layers	-398	152	-2.62	0.010**	-700	-97
Color of top layer:						
beige, orange or brown	490	370	1.32	0.188	-240	1210
black	400	360	1.12	0.266	-310	1110
blue or green	-420	480	-0.88	0.380	-1370	530
grey	390	340	1.14	0.257	-290	1070
red	840	610	1.39	0.167	-360	2040
transparent or white	50	360	0.13	0.896	-660	750
wood	-260	320	-0.81	0.420	-880	370

*** significant contributions ($p < 0.05$)*
** possibly significant contributions ($p < 0.10$)*

S3.5.2 *ortho*-Phthalates – presence and concentration

S3.5.2.1 Any ortho*-phthalates*

Table S 17: Linear regression model for predicting the chance of any of *ortho*-phthalates being present (in %) based on sample properties (independent variables).

**Chance of <u>presence</u> of any *ortho*-phthalates [%]**	**Coefficient**	**std err**	**t-value**	**p-value**	**[0.025**	**0.975]**
**Constant**	84.5	10.0	8.458	0.000**	64.7	104.2
**Independent Variables**						
Originating from DIY store	-64.6	9.4	-6.859	0.000**	-83.2	-46.0
Presence of grey layer	9.1	9.8	0.928	0.355	-10.3	28.4
Hardness	-12.9	6.0	-2.158	0.033**	-24.7	-1.1
Number of layers	4.4	3.7	1.205	0.230	-2.8	11.7
Color of top layer:						
beige, orange or brown	-3.1	8.9	-0.350	0.727	-20.6	14.4
black	18.7	8.7	2.161	0.032**	1.6	35.8
blue or green	22.4	11.6	1.930	0.056*	-0.6	45.3
grey	10.1	8.3	1.226	0.222	-6.2	26.4
red	14.6	14.7	0.999	0.319	-14.3	43.6
transparent or white	8.7	8.6	1.008	0.315	-8.3	25.7
wood	13.1	7.6	1.711	0.089	-2.0	28.1

*** significant contributions ($p < 0.05$)*
** possibly significant contributions ($p < 0.10$)*

Table S 18: Linear regression model for predicting the concentration (in wt%) of *ortho*-phthalates based on sample properties (independent variables).

**<u>Concentration</u> of *ortho*-phthalates [wt%]**	**Coefficient**	**std err**	**t-value**	**p-value**	**[0.025**	**0.975]**
**Constant**	9.81	2.10	4.672	0.000**	5.66	13.96
**Independent Variables**						
Originating from DIY store	-3.62	1.98	-1.829	0.070*	-7.53	0.29
Presence of grey layer	1.38	2.10	0.670	0.504	-2.69	5.44
Hardness	-3.29	1.26	-2.617	0.010**	-5.77	-0.80
Number of layers	0.55	0.77	0.705	0.482	-0.98	2.07
Color of top layer:						
beige, orange or brown	1.19	1.86	0.641	0.522	-2.49	4.88
black	7.39	1.82	4.060	0.000**	3.79	10.99
blue or green	1.42	2.43	0.584	0.560	-3.39	6.24
grey	0.35	1.73	0.202	0.840	-3.08	3.78
red	-1.16	3.10	-0.376	0.708	-7.25	4.93
transparent or white	-0.30	1.81	-0.167	0.868	-3.88	3.28
wood	0.91	1.60	0.568	0.571	-2.26	4.08

*** significant contributions ($p < 0.05$)*
** possibly significant contributions ($p < 0.10$)*

S3.5.2.2 Restricted ortho-*phthalates*

Table S 19: Linear regression model for predicting the chance of regulated *ortho*-phthalates being present (in %) based on sample properties (independent variables).

**Chance of presence of any regulated *ortho*-phthalates [%]**	**Coefficient**	**std err**	**t-value**	**p-value**	**[0.025**	**0.975]**
**Constant**	48.9	10.2	4.805	0.000**	28.8	69.0
**Independent Variables**						
Originating from DIY store	-25.9	9.6	-2.698	0.008**	-44.8	-6.9
Presence of grey layer	1.5	10.0	0.148	0.883	-18.2	21.2
Hardness	-12.4	6.1	-2.039	0.043**	-24.4	-0.4
Number of layers	6.2	3.7	1.643	0.103	-1.3	13.6
Color of top layer:						
beige, orange or brown	-1.0	9.0	-0.112	0.911	-18.9	16.8
black	9.8	8.8	1.113	0.267	-7.6	27.2
blue or green	6.2	11.8	0.529	0.598	-17.1	29.6
grey	5.9	8.4	0.700	0.485	-10.7	22.5
red	35.6	14.9	2.383	0.018**	6.1	65.1
transparent or white	-6.2	8.8	-0.705	0.482	-23.5	11.2
wood	-1.4	7.8	-0.186	0.853	-16.8	13.9

*** significant contributions ($p < 0.05$)*
** possibly significant contributions ($p < 0.10$)*

Table S 20: Linear regression model for predicting the concentration (in wt%) of regulated *ortho*-phthalates based on sample properties (independent variables).

**Concentration of regulated *ortho*-phthalates [wt%]**	**Coefficient**	**std err**	**t-value**	**p-value**	**[0.025**	**0.975]**
**Constant**	1.5657	0.504	3.104	0.002**	0.568	2.563
**Independent Variables**						
Originating from DIY store	-0.7691	0.476	-1.617	0.108	-1.710	0.171
Presence of grey layer	1.3389	0.494	2.711	0.008**	0.363	2.315
Hardness	-0.3289	0.302	-1.090	0.278	-0.926	0.268
Number of layers	-0.3992	0.186	-2.149	0.033**	-0.766	-0.032
Color of top layer:						
beige, orange or brown	0.2567	0.448	0.573	0.567	-0.629	1.142
black	0.1903	0.437	0.435	0.664	-0.674	1.055
blue or green	-0.3888	0.585	-0.664	0.508	-1.546	0.769
grey	1.2160	0.417	2.917	0.004**	0.392	2.040
red	0.0411	0.740	0.055	0.956	-1.423	1.505
transparent or white	0.2694	0.435	0.619	0.537	-0.591	1.130
wood	-0.0188	0.385	-0.049	0.961	-0.781	0.743

*** significant contributions ($p < 0.05$)*
** possibly significant contributions ($p < 0.10$)*

S3.5.3 Alternative plasticizers – presence

Table S 21 Linear regression model for predicting the chance of alternative plasticizers being present (in %) based on sample properties (independent variables).

**Chance of presence of any alternative plasticizers [%]**	**Coefficient**	**std err**	**t-value**	**p-value**	**[0.025**	**0.975]**
**Constant**	23.3403	9.2	2.538	0.012	5.157	41.523
**Independent Variables**						
Originating from DIY store	10.0927	8.7	1.164	0.247	-7.055	27.241
Presence of grey layer	11.9910	9.0	1.332	0.185	-5.810	29.792
Hardness	9.2636	5.5	1.683	0.095*	-1.616	20.144
Number of layers	6.1668	3.4	1.821	0.071*	-0.528	12.862
Color of top layer:						
beige, orange or brown	11.9120	8.2	1.459	0.147	-4.231	28.055
black	-17.2277	8.0	-2.161	0.032**	-32.990	-1.466
blue or green	12.8578	10.7	1.205	0.230	-8.246	33.961
grey	-1.4693	7.6	-0.193	0.847	-16.493	13.555
red	-2.9662	13.5	-0.220	0.826	-29.652	23.720
transparent or white	10.3472	7.9	1.304	0.194	-5.341	26.036
wood	9.8865	7.0	1.407	0.162	-4.006	23.779

*** significant contributions (p < 0.05)*
** possibly significant contributions (p < 0.10)*

S3.5.4 Bioassays

Table S 22: Linear regression model for predicting the chance of activity in any of the bioassay (in %) based on sample properties (independent variables).

**Chance of activity in bioassay [%]**	**Coefficient**	**std err**	**t-value**	**p-value**	**[0.025**	**0.975]**
**Constant**	102.4436	20.998	4.879	0.000**	60.605	144.283
**Independent Variables**						
Originating from DIY store	-70.4280	19.359	-3.638	0.001**	-109.00	-31.854
Presence of grey layer	-22.8974	20.128	-1.138	0.259	-63.003	17.208
Hardness	-15.3879	10.606	-1.451	0.151	-36.521	5.745
Number of layers	8.0993	6.411	1.263	0.210	-4.676	20.874
Color of top layer:						
beige, orange or brown	14.6191	15.866	0.921	0.360	-16.995	46.233
black	19.8693	14.356	1.384	0.171	-8.736	48.474
blue or green	22.2694	24.332	0.915	0.363	-26.213	70.752
grey	20.1881	13.596	1.485	0.142	-6.903	47.279
red	-25.1825	22.201	-1.134	0.260	-69.420	19.055
transparent or white	24.6362	14.903	1.653	0.103	-5.058	54.331
wood	26.0439	12.378	2.104	0.039**	1.381	50.707

*** significant contributions (p < 0.05)*
** possibly significant contributions (p < 0.10)*

S3.6 Screening quality metrics

Table S 23: Quality of different screening methods for determining samples of clear concern and those of any concern (possible + clear concern) using confusion matrices, sensitivity (sens) and specificity (spec).

<table>
<tr><th></th><th>Any concern</th><th></th><th>Clear concern</th><th></th></tr>
<tr><td>FTIR screening</td><td>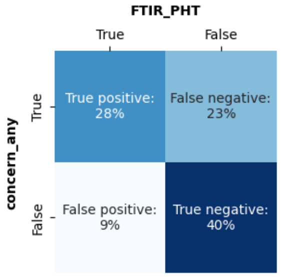
</td><td>sens: 55.8 %
spec: 81.1 %</td><td>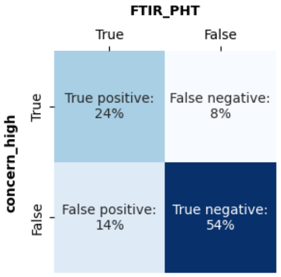
</td><td>sens: 75.0%
spec: 79.6%</td></tr>
<tr><td>XRF screening – any toxic elements</td><td>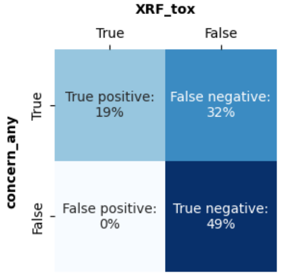
</td><td>sens: 37.7 %
spec: 100.0 %</td><td>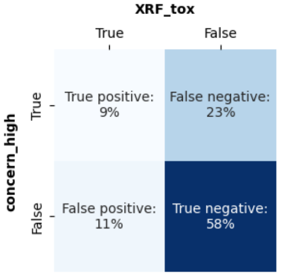
</td><td>sens: 27.1 %
spec: 84.5 %</td></tr>
<tr><td>XRF screening – >0.1wt% toxic elements</td><td>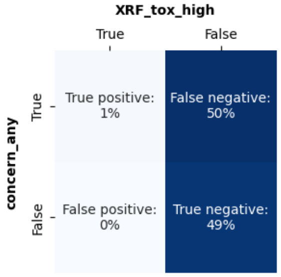
</td><td>sens: 1.3 %
spec: 100.0 %</td><td>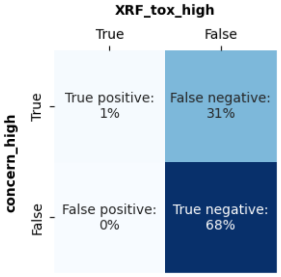
</td><td>sens: 2.1 %
spec: 100.0 %</td></tr>
<tr><td>GCMS – >0.1wt% ortho-phthlates</td><td>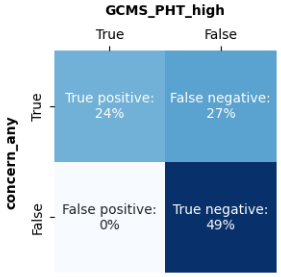
</td><td>sens: 46.8 %
spec: 100.0 %</td><td>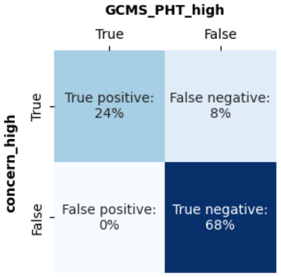
</td><td>sens: 75.0 %
spec: 100.0 %</td></tr>
<tr><td>Active in bioassays</td><td>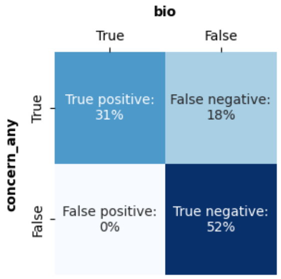
</td><td>sens: 63.4 %
spec: 100.0 %</td><td>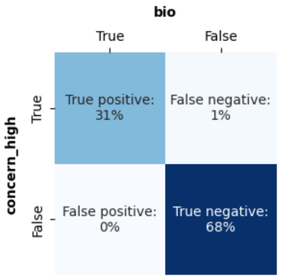
</td><td>sens: 96.3 %
spec: 100.0 %</td></tr>
</table>

	concern_any	concern_high
Presnece of a grey layer	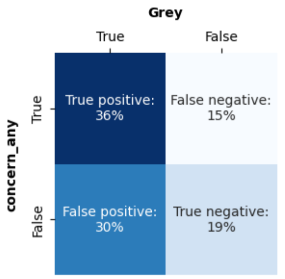 sens: 70.1 %<br>spec: 39.2 %	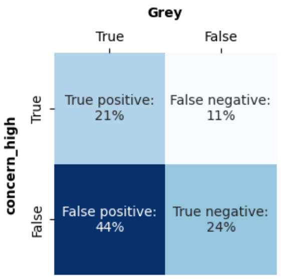 sens: 66.7 %<br>spec: 35.0 %
Hard sample	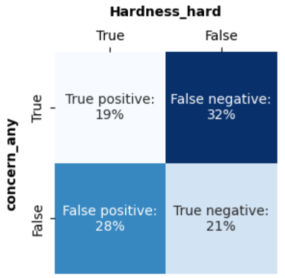 sens: 36.4 %<br>spec: 43.2 %	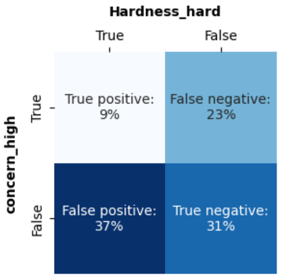 sens: 29.2 %<br>spec: 45.6 %
Medium-hard sample	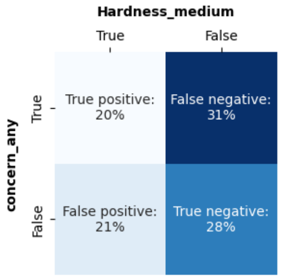 sens: 39.0 %<br>spec: 56.8 %	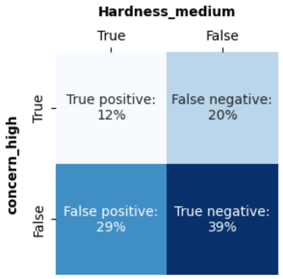 sens: 37.5 %<br>spec: 57.3 %
Soft sample	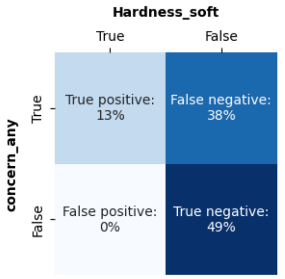 sens: 24.7 %<br>spec: 100.0 %	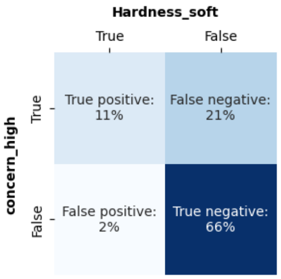 sens: 33.3 %<br>spec: 97.1 %
Color on top: beige, orange or brown	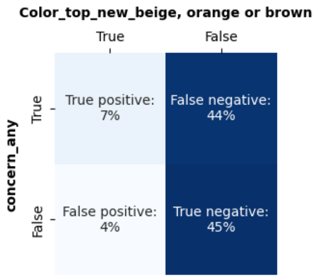 sens: 13.0 %<br>spec: 91.9 %	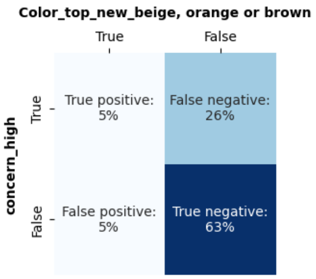 sens: 16.7 %<br>spec: 92.2 %
Color on top: black	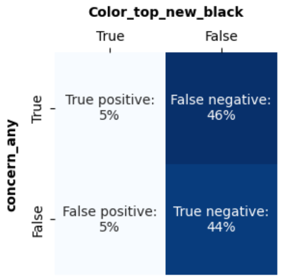 sens: 10.4 %<br>spec: 89.2 %	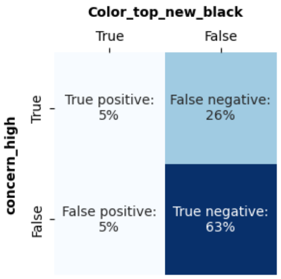 sens: 16.7 %<br>spec: 92.2 %

<table>
<tr><td>Color on top:
blue or green</td><td>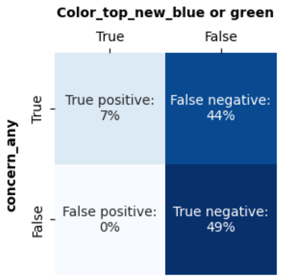

sens: 13.0 %

spec: 100.0 %</td><td>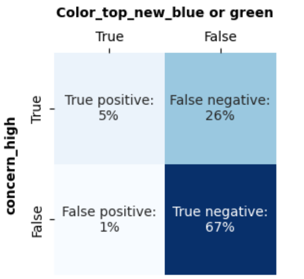

sens: 16.7 %

spec: 98.1 %</td></tr>
<tr><td>Color on top:
grey</td><td>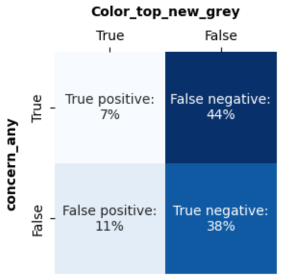

sens: 14.3 %

spec: 77.0 %</td><td>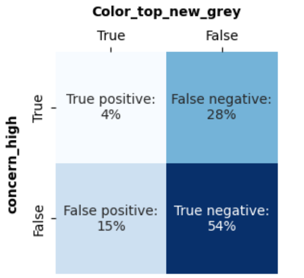

sens: 12.5 %

spec: 78.6 %</td></tr>
<tr><td>Color on top:
red</td><td>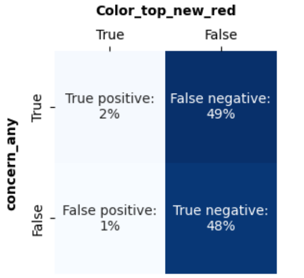

sens: 3.9 %

spec: 97.3 %</td><td>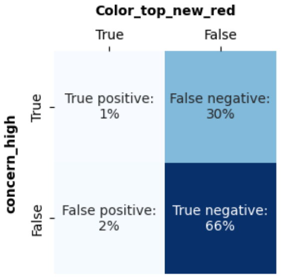

sens: 4.2 %

spec: 97.1 %</td></tr>
<tr><td>Color on top:
transparent or white</td><td>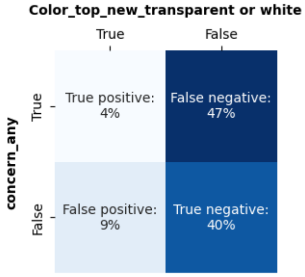

sens: 7.8 %

spec: 82.4 %</td><td>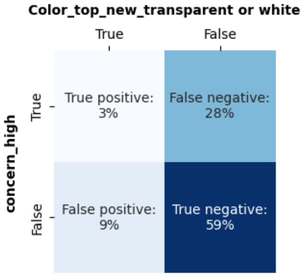

sens: 10.4 %

spec: 86.4 %</td></tr>
<tr><td>Color on top:
wood</td><td>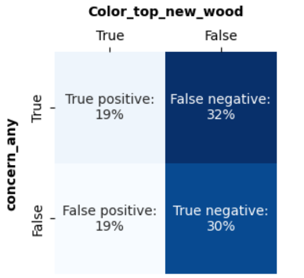

sens: 37.7 %

spec: 62.2 %</td><td>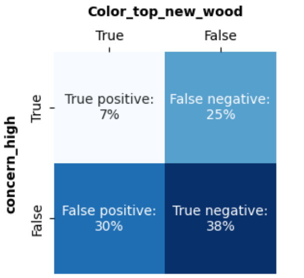

sens: 22.9 %

spec: 55.3 %</td></tr>
</table>

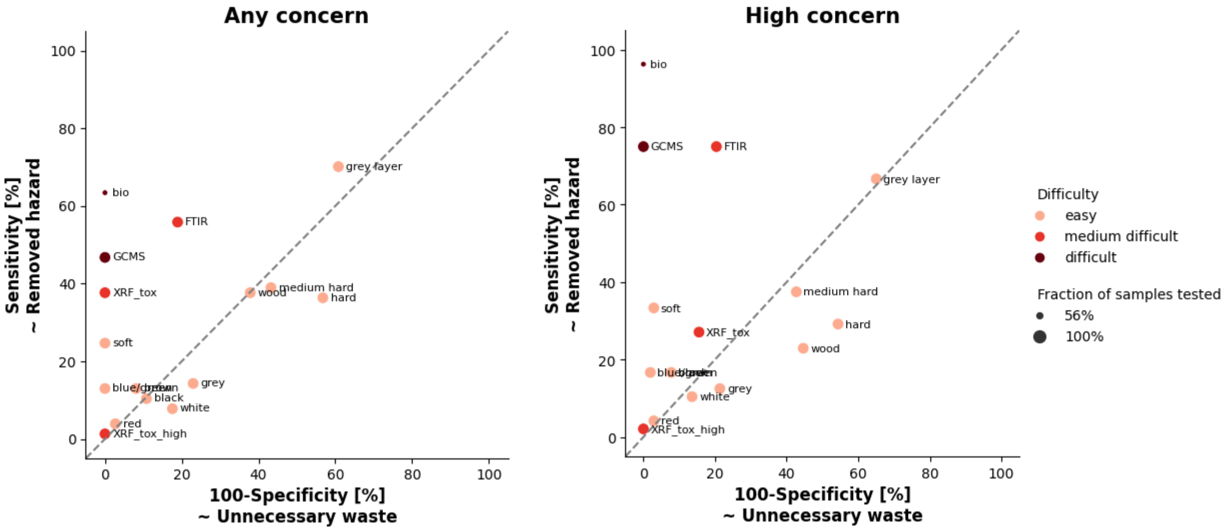

Figure S 15: Utility of different screening methods. Reverse specificity (as a proxy for unnecessary waste) is plotted against sensitivity (as a proxy for removed hazardous substances) for selected screening methods. Methods are differentiated by how difficult it is to implement them on industrial scale for waste sorting and by the fraction of samples tested in our study.

S4 DISCUSSION

S4.1 Chemical substances in PVC flooring

Table S 24 Recent studies investigating plasticizers and other substances present in PVC flooring. The country was not specified for all studies, the location of the main authors are given in parenthesis if no details were mentioned.

Reference	Country	Year	n	Major plasticizers	Conc. range [wt%]	Other substances	Conc. range [wt%]
**Clausen, et. al (2004)** [18]	(DNK)	2004	1	DEHP	17	not analyzed	-
**Afshari, et. al (2004)** [19]	(DNK)	2004	4	DEHP	17-18.5	not analyzed	-
**Chino, et.al. (2009)** [20]	(JPN)	2009	1	DEHP	10	not analyzed	-
**Xu, et al. (2012)** [21]	DNK	2012	1	DEHP	15	not analyzed	-
**Kumari, et al. (2014)** [22]	IND	2014	1	not analyzed	-	BDE47, BDE153, BDE209	<LOD
**Liang, et al. (2015)** [23]	USA	2015	16	DEHP, BBP, DEHI, DiNP, DBP	0.03-26.5	not analyzed	-
**Shi, et al. (2018)** [24]	CHN	2018	2	DEHP, BBP, DnOP (only low MW *ortho*-phthalates analyzed)	4-15	not analyzed	-
**Bohlin-Nizzetto, et al. (2021)** [25]	NOR	2021	6	TPhP, TBEP (*ortho*-phthalates not analyzed)	0.0002-0.07	BFRs	<LOD – $7x10^{-8}$
**Lowe, et al. (2021)** [26]	USA	2021	43	DEHA, DEP, TXIB, DBP, ATBC, BBP, others	not quant.	Hexadecanoic acid, Octadecanoic acid, 1-Dodecanol, others	not quant.
**This study**	CHE	2021	151	DEHT, DiNP, DEHA, DEHP, DiDP, Octicizer, others	<LOD - 46	UV326, BPA	not quant.

S4.2 Chemical substances in other PVC products

Table S 25: Recent studies investigating stabilizers and other metals present in PVC products.

Reference	Country	Year	Product	n	Major stabilizer	Conc. of restricted [wt%]
**Kumar, et al. (2007)** [27]	IND	2007	Toys	77	Pb, Cd (only Pb & Cd investigated)	Cd: <LOD - 0.018<br>Pb: <LOD - 0.21
**Ismail, et al. (2017)** [28]	MYS	2017	Toys	21	Zn (100%), Ba (62%), Pb (38%), Sn (14%), Cd (14%)	Cd: 0.0020<br>Pb: 0.011
**Oyeyiola, et al. (2017)** [29]	NGA	2017	Toys	21	Pb, Cd (only Pb & Cd investigated)	Cd: <LOD - 0.004<br>Pb: <LOD - 0.011
**Meng, et al. (2021)** [30]	CHN	2021	B&C - Clapboard	1	Pb, Si, Ti, Ca	PbO: 0.3
**Turner, et al. (2021)** [31]	GBR	2021	Several	92	Ba (49%), Pb (25%), Sn (20%), Sb (12%), Zn (9%), Cd (2%)	Cd: 0.15-0.16<br>Pb: 0.16-2.5

Table S 26: Recent studies investigating plasticizers and other organic substances present in other PVC products (not flooring). The country was not specified for all studies, the location of the main authors are given in parenthesis if no details were mentioned.

Reference	Country	Year	Product	n	Major plasticizers	Conc. range [wt%]	Other substances	Conc. range [wt%]
**Wahl, et al. (1999)** [32]	GER	1999	Medical	6	DEHP, BEHP, DBP, DiBP, DEP, DEHA, DMP	not quant. (DEHP largest area)	BHT, Styrene, others	not quant.
**Wang, et al. (2005)** [33]	DNK	2005	Medical	3	DEHP, DCHP, DEHA	0.06-30	BHT	not quant.
**Welle, et al. (2005)** [34]	(GER)	2005	Medical	6	DEHP, DiNCH, TEHTM, ATBC	30-49	not analyzed	-
**Radaniel, et al. (2014)** [35]	GER	2014	Medical	5	DEHP, ATBC, DEHT, DiNCH, TEHTM (sampled tubing with known contetnt for method validation)	29-36	not analyzed	-
**Bernhard, et al. (2015)** [36]	FRA	2015	Medical	4	DEHP, DEHT, TEHTM, DiNCH	28-31	not analyzed	-
**Bourdeaux et al. (2016)** [37]	FRA	2016	Medical	32	TEHTM, DEHP, DiNCH, DiNP, ATBC, DEHA, DEHT	24-36	not analyzed	-
**Faessler, et al. (2017)** [38]	CHE	2017	Medical	7	DEHP, DiNCH, DEHT, TOTM, ESBO	22-44	not analyzed	-
**Jeon, et al. (2018)** [39]	KOR	2018	Medical	3	DEHP, DiOP, TEHTM	not quant.	not analyzed	-
**Fernandez-Canal, et al. (2018)** [40]	(FRA)	2018	Medical	1	TOTM, DEHP, DEHT, DEHA	0.1-45	not analyzed	-
**Den Braver-Sewradj, et al. (2020)** [41]	-	2020	Medical	-	Review (extensive use of DEHP, mail alternatives: TEHTP, DiNCH, DEHA, ATBC, DiNP)	-	not analyzed	-
**Rastogi, et al. (1998)** [42]	(DNK)	1998	Toys	7	DEHP, DiNP, DiDP	<LOD - 40	not analyzed	-
**US-CPSC (2010)** [43]	USA	2010	Toys	37	ATBC (60%), Tributyl aconitate (49%), DiNCH (38%), DEHT (35%), TXIB (32%), DEHP (3%), DiNP (3%)	14-42	not analyzed	-
**Al-Natsheh, et al. (2015)** [44]	JOR	2015	Toys	1	DEHP	0.06	Not analyzed	-
**McCombie, et al. (2017)** [45]	CHE	2017	Toys	118	ESBO (81%), DEHT (55%), TXIB (49%), DiNCH (31%), ATBC (31%), DEHP (9%), others	0.9-51	not analyzed	-
**Ashworth, et al. (2018)** [46]	NZL	2018	Toys	49	DEHP, DiNP, DiDP, DiBP, DBP, DNOP	0.1-54	not analyzed	-

S4.3 Exposure to *ortho*-Phthalates and alternative plasticizers

Usually, the major exposure pathway for all *ortho*-phthalates is dietary intake, in the µg kg_{bw}^{-1} d^{-1} range, and together with indoor exposure, relevant health limit values (e.g., a reference dose for *DEHP*: 20 µg kg_{bw}^{-1} d^{-1}) can be exceeded, especially for vulnerable and at-risk populations (e.g., toddlers).[47] Another noteworthy exposure pathway for specific individuals is from medical devices, which are still commonly plasticized with DEHP as allowed by a re-authorization process: exposure from intravenous administration of different solutions may reach up the mg kg_{bw}^{-1} d^{-1} range.[41,48] The most important indoor exposure pathways for (semi-volatile) plasticizers such as *ortho*-phthalates from indoor products are the ingestion of dust, inhalation of air-borne particles, and direct skin contact (Table S 28).[18,49–52] While for higher-molecular weight *ortho*-phthalates, dust and dietary intake dominate the total exposure, for lower-molecular weight *ortho*-phthalates (e.g., DMP, DEP, DBP, DiBP), inhalation and dermal uptake (due to use in personal care products) are additionally important.[47,53,54] Typically, steady-state air concentrations for *ortho*-phthalates have been found in the low µg/m^3 range in chamber experiments with PVC floorings (e.g., 0.8–1 µg/m^3 for DEHP),[19,21,55] and air measurements in residential buildings (e.g., 0.1–20 µg/m^3 for total phthalates) [56,57]. This clearly demonstrates the releases of these substances from PVC floorings. Once released from the PVC matrix, partition to skin, dust, and air-borne particles is mainly governed by the octanol-air partition coefficients K_{OA}, which is high for the major plasticizers in this study (Figure S 1, Table S 4).[58–61] A similar pattern to the original PVC flooring is, thus, expected in dust and skin wipes. Plasticizers have been measured in indoor dust samples in the µg/g to mg/g range, with strong correlation with the use of PVC floorings as can be expected.[52,56,57,62–64] The main plasticizers in dust vary by region, likely due to different flooring compositions across markets. For example, a recent Swedish study found mainly DiNP, DEHP, DiDP, DEHT, and DINCH (~100 µg/g), which is in good agreement with our findings.[62] Another German study found DEHP, DiNP, and DiDP being the major ones, and DEHP reducing and the others rising over time.[64] A study in Canada found mainly DiNP, DEHP, and DBP (~14-200 µg/g).[57] Studies from China, Republic of Korea and the US mainly reported DEHP, DBP, DiBP, and BBP (also ~100 µg/g).[52,54,63,65]. In general, exposure from dust ingestion has been estimated to be in the lower µg kg_{bw}^{-1} d^{-1}, while dermal absorption of dust is in the low ng kg_{bw}^{-1} d^{-1}.[52,54,57] Concentrations of *ortho*-phthalates on skin have typically been measured in the ng/cm^2 to µg/cm^2 range, resulting in estimated dermal exposure to air in the lower µg kg_{bw}^{-1} d^{-1} range. Dermal

exposure is typically lower than exposure from dust ingestion.[47,52,54,57,66] Reported plasticizers on skin were again regionally dependent: DiNP, DNOP, DEHP, DBEP, and DMEP were the main plasticizers in China,[66] DEHP and DiNP were reported in the US and Canada,[52,57] and DiNP, DEHP, and DiDP were the main plasticizers reported in Norway (which shows a similar plasticizer profile as in this study).[47]

Alternatives are found in similar concentrations, albeit slightly lower than *ortho*-phthalates, in the different compartments (Table S 27): DEHA, DINCH, DEHT and ATBC were found in the air around 10–100 ng/m^3 (an order of magnitude below *ortho*-phthalates),[67,68] DEHA, DINCH, DEHT, and ATBC were found in dust around 10–100 μg/g (the same order of magnitude as *ortho*-phthalates).[62,68,69]

Table S 27: Measured and modelled indoor media concentrations of different plasticizers. M= measurements, C= chamber / model

		Type of study	***ortho*-phthalates** Restricted *DEHP*	*BBP, DBP, DiBP*	**Other o-PHT** *DiNP, DiDP*	*Others*	**Alternative plasticizers Detected in this study** *e.g. DEHT, DEHA, DINCH*	**Other**	**Ref**
	logKoa		11.7	8.2-9.8	11-11.5	5.7-11.7	10.8-11.7	6-18	[70]
**Conc.**	*Flooring [μg/g]*	M	32–204’700	39-4’000	500-471’300	50-18’600	80-1’000’000*	n.a.	This study
	Air [μg/m³]	M	0.02-3.69	0.0006-4.6	0.01-0.03	0.004-2.5	<.LOD	-	[47,57,71,72]
		C	0.8–1.0	0.1-0.2	-	-	-	-	[19,21,55]
	Dust [μg/g]	M	100-232	5.5-15.2	29-282	0.12-6.3	32.8-34.5	-	[47,57,62]
		C	0.5-0.9	-	-	-	-	-	[55]
	Dust [μg/m3]	M	0.04-2.2	0.0003-2.3	-	0.0002-1.5	-	-	[72,73]
		C	0.5-0.9	-	-	-	-	-	[55]
	Skin [μg/m2]	M	0.000001-55.7	0.0001-2.7	0.0001-56.9	0.0001-3.6	<LOD	-	[47,57]
	Surface [μg/m2]	M	0.000001-1’241	0.0001-0.004	0.00001-0.037	0.00003-0.0006	-	-	[21,57]

* alternative plasticizer concentrations in this study are highly uncertain, due to lack of internal standard and in-range calibration, which lead to partially implausible estimates

** PVC flooring in a ventilated room

https://doi.org/10.1021/acs.est.3c04851

Table S 28 Estimated exposure to different plasticizers.

		ortho-phthalates				Alternative plasticizers		
		**Restricted**		**Other o-PHT**		**Detected in this study**	**Other**	**Ref**
		DEHP	*BBP, DBP, DiBP*	*DiNP, DiDP*	*Others*	*e.g. DEHT, DEHA, DINCH*		
**Exposure [µg/kg/d]**	*Total*	1.36-2.7	0.22-0.99	0.3-1.6	0.03-135	0.1-8	0.004-4’650	[47,74]
	Dietary	1.26	0.14-0.64	0.16-0.21	0.01-0.31	0.22	-	[47]
	Medical devices	6-13’070	-	-	-	3-300	-	[41,75]
	Indoor	0.15	0.012-0.18	0.024-0.108	0.004-0.135	0.023	-	[47]
	-dust ingestion	0.14	0.006-0.011	0.021-0.106	0.0001-0.004	0.023	-	[47]
	- inhalation	0.019	0.002-0.113	0.002-0.003	0.01-0.06	0.001	-	[47]
	- dermal	0.00038	0.046-0.060	0.0000025-0.000014	0.0003-0.034	0.0001	-	[47]
**TDI [µg/kg/d]**		50	10-500	150	200-500	40-25’000	460-1’250	[47,74,76]

https://doi.org/10.1021/acs.est.3c04851

REFERENCES

(1) European Chemicals Agency (ECHA). Candidate List of substances of very high concern for Authorisation https://echa.europa.eu/candidate-list-table (accessed Apr 2, 2020).

(2) European Chemicals Agency (ECHA). SCIP Database https://echa.europa.eu/scip-database (accessed Sep 25, 2023).

(3) European Chemicals Agency (ECHA). Authorisation List https://echa.europa.eu/authorisation-list (accessed Sep 25, 2023).

(4) European Chemicals Agency (ECHA). Restriction List https://echa.europa.eu/substances-restricted-under-reach.

(5) European Chemicals Agency (ECHA). Phthalates https://echa.europa.eu/hot-topics/phthalates (accessed Sep 25, 2023).

(6) European Parliament; Council of the European Union. Council Directive 2009/48/EC on the safety of toys http://data.europa.eu/eli/dir/2009/48/2019-11-18 (accessed Sep 25, 2023).

(7) European Parliament; Council of the European Union. Council Directive 2005/84/EC on phthalates in toys and childcare articles https://eur-lex.europa.eu/eli/dir/2005/84/oj (accessed Sep 25, 2023).

(8) European Parliament; Council of the European Union. Council Directive 2011/65/EU on the restriction of the use of certain hazardous substances (RoHS) in electrical and electronic equipment (EEE) http://data.europa.eu/eli/dir/2011/65/2021-04-01 (accessed Sep 25, 2023).

(9) vinylPlus. The European PVC industry's experience in replacing lead and cadmium-based stabilisers https://www.stabilisers.eu/wp-content/uploads/2015/11/VinylPlus_Contribution-Cefic_Eu-Industry.pdf (accessed Sep 25, 2023).

(10) European Stabilisers Producers Association (ESPA(). Stabilisers – What's new ? https://www.stabilisers.eu/wp-content/uploads/2016/01/ESPA-stabilisers_update_January-2017.pdf (accessed Sep 25, 2023).

(11) Everard, M. 5 - PVC and Sustainability. In *PVC Additives*; Schiller, M., Ed.; Hanser, 2015; pp 369–410. https://doi.org/https://doi.org/10.3139/9781569905449.005.

(12) Kuptsov, A. H.; Zhizhin, G. N. Spectra. In *Handbook of Fourier Transform Raman and Infrared Spectra of Polymers*; Kuptsov, A. H., Zhizhin, G. N. B. T.-P. S. D., Eds.; Elsevier, 1998; Vol. 45, pp 1–500. https://doi.org/10.1016/S0921-318X(98)80016-7.

(13) Thermo Scientific. Fast , affordable solutions for polymers and plastics analysis https://assets.thermofisher.com/TFS-Assets/MSD/Flyers/FL52273-ftir-polymer-analysis-kits.pdf.

(14) Lowry, S.; Bradley, M.; Thermo Scientific. Using FT-IR Spectroscopy to Characterize Plastics and Other Materials. *Adv. Mater. Process.* **2011**, *169* (4), 22–25.

(15) Agilent; Wang, Y. Polymer and Phthalate Analysis with FTIR Spectroscopy. Agilent 2018.

(16) Thermo Fisher Scientific. XL3 Analyzer version 7.0.1 User Guide https://www.tttenviro.com/wp-content/uploads/Manual-XL3-Series-v7.0.11.pdf.

(17) Christen, V.; Camenzind, M.; Fent, K. Silica Nanoparticles Induce Endoplasmic Reticulum Stress Response, Oxidative Stress and Activate the Mitogen-Activated Protein Kinase (MAPK) Signaling Pathway. *Toxicol. Reports* **2014**, *1*, 1143–1151. https://doi.org/10.1016/j.toxrep.2014.10.023.

(18) Clausen, P. A.; Hansen, V.; Gunnarsen, L.; Afshari, A.; Wolkoff, P. Emission of Di-2-Ethylhexyl Phthalate from PVC Flooring into Air and Uptake in Dust: Emission and Sorption Experiments in FLEC and CLIMPAQ. *Environ. Sci. Technol.* **2004**, *38* (9), 2531–2537. https://doi.org/10.1021/es0347944.

(19) Afshari, A.; Gunnarsen, L.; Clausen, P. A.; Hansen, V. Emission of Phthalates from PVC and Other Materials. *Indoor Air* **2004**, *14* (2), 120–128. https://doi.org/10.1046/j.1600-0668.2003.00220.x.

(20) Chino, S.; Kato, S.; Seo, J.; Ataka, Y. Study on Emission of Decomposed Chemicals of Esters Contained in PVC Flooring and Adhesive. *Build. Environ.* **2009**, *44* (7), 1337–1342. https://doi.org/10.1016/j.buildenv.2008.07.003.

(21) Xu, Y.; Liu, Z.; Park, J.; Clausen, P. A.; Benning, J. L.; Little, J. C. Measuring and Predicting the Emission Rate of Phthalate Plasticizer from Vinyl Flooring in a Specially-Designed Chamber. *Environ. Sci. Technol.* **2012**, *46* (22), 12534–12541. https://doi.org/10.1021/es302319m.

(22) Kumari, K.; Sharma, J. K.; Kanade, G. S.; Kashyap, S. M.; Juwarkar, A. A.; Wate, S. R. Investigation of Polybrominated Diphenyl Ethers in Old Consumer Products in India. *Environ. Monit. Assess.* **2014**, *186* (5), 3001–3009. https://doi.org/10.1007/s10661-013-3596-2.

(23) Liang, Y.; Xu, Y. Emission of Phthalates and Phthalate Alternatives from Vinyl Flooring and Crib Mattress Covers: The Influence of Temperature. *Environ. Sci. Technol.* **2014**, *48* (24), 14228–14237. https://doi.org/10.1021/es504801x.

(24) Shi, S.; Cao, J.; Zhang, Y.; Zhao, B. Emissions of Phthalates from Indoor Flat Materials in Chinese Residences. *Environ. Sci. Technol.* **2018**, *52* (22), 13166–13173. https://doi.org/10.1021/acs.est.8b03580.

(25) Bohlin-Nizzetto, P. Content and migration of chemical additives from plastic products. (NILU report 9/2022). https://hdl.handle.net/11250/2992965 (accessed Sep 25, 2023).

(26) Lowe, C. N.; Phillips, K. A.; Favela, K. A.; Yau, A. Y.; Wambaugh, J. F.; Sobus, J. R.; Williams, A. J.; Pfirrman, A. J.; Isaacs, K. K. Chemical Characterization of Recycled Consumer Products Using Suspect Screening Analysis. *Environ. Sci. Technol.* **2021**, *55* (16), 11375–11387. https://doi.org/10.1021/acs.est.1c01907.

(27) Kumar, A.; Pastore, P. Lead and cadmium in soft plastic toys http://www.jstor.org/stable/24099126 (accessed Sep 25, 2023).

(28) Ismail, S. N. S.; Mohamad, N. S.; Karuppiah, K.; Abidin, E. Z.; Rasdi, I.; Praveena, S. M. Heavy metals content in low-priced toys http://www.arpnjournals.org/jeas/research_papers/rp_2017/jeas_0317_5787.pdf (accessed Sep 25, 2023).

(29) Oyeyiola, A. O.; Akinyemi, M. I.; Chiedu, I. E.; Fatunsin, O. T.; Olayinka, K. O. Statistical Analyses and Risk Assessment of Potentially Toxic Metals (PTMS) in Children's Toys. *J.*

Taibah Univ. Sci. **2017**, *11* (6), 842–849. https://doi.org/10.1016/j.jtusci.2017.02.005.

(30) Meng, J.; Xu, B.; Liu, F.; Li, W.; Sy, N.; Zhou, X.; Yan, B. Effects of Chemical and Natural Ageing on the Release of Potentially Toxic Metal Additives in Commercial PVC Microplastics. *Chemosphere* **2021**, *283* (October 2020), 131274. https://doi.org/10.1016/j.chemosphere.2021.131274.

(31) Turner, A.; Filella, M. Polyvinyl Chloride in Consumer and Environmental Plastics, with a Particular Focus on Metal-Based Additives. *Environ. Sci. Process. Impacts* **2021**, *23* (9), 1376–1384. https://doi.org/10.1039/d1em00213a.

(32) Wahl, H. G.; Hoffmann, A.; Häring, H.-U.; Liebich, H. M. Identification of Plasticizers in Medical Products by a Combined Direct Thermodesorption–Cooled Injection System and Gas Chromatography–Mass Spectrometry. *J. Chromatogr. A* **1999**, *847* (1–2), 1–7. https://doi.org/10.1016/S0021-9673(99)00138-7.

(33) Wang, Q.; Storm, B. K. Separation and Analysis of Low Molecular Weight Plasticizers in Poly(Vinyl Chloride) Tubes. *Polym. Test.* **2005**, *24* (3), 290–300. https://doi.org/10.1016/j.polymertesting.2004.12.002.

(34) Welle, F.; Wolz, G.; Franz, R. Migration of plasticizers from PVC tubes into enteral feeding solutions http://pieweb.plasteurope.com/members/pdf/P204322b.PDF (accessed Sep 25, 2023).

(35) Radaniel, T.; Genay, S.; Simon, N.; Feutry, F.; Quagliozzi, F.; Barthélémy, C.; Lecoeur, M.; Sautou, V.; Décaudin, B.; Odou, P.; Bernard, L.; Bourdeaux, D.; Chennell, P.; Richard, D.; Pereira, B.; Azaroual, N.; Christine Barthélémy; Décaudin, B.; Dine, T.; Feutry, F.; Genay, S.; Kambia, N.; Lecoeur, M.; Odou, P.; Simon, N.; Vaccher, C.; Cueff, R.; Feschet, E.; Breysse, C. Quantification of Five Plasticizers Used in PVC Tubing through High Performance Liquid Chromatographic-UV Detection. *J. Chromatogr. B Anal. Technol. Biomed. Life Sci.* **2014**, *965*, 158–163. https://doi.org/10.1016/j.jchromb.2014.06.027.

(36) Bernard, L.; Cueff, R.; Breysse, C.; Décaudin, B.; Sautou, V. Migrability of PVC Plasticizers from Medical Devices into a Simulant of Infused Solutions. *Int. J. Pharm.* **2015**, *485* (1–2), 341–347. https://doi.org/10.1016/j.ijpharm.2015.03.030.

(37) Bourdeaux, D.; Yessaad, M.; Chennell, P.; Larbre, V.; Eljezi, T.; Bernard, L.; Sautou, V.; Azaroual, N.; Barthelémy, C.; Décaudin, B.; Dine, T.; Feutry, F.; Genay, S.; Kambia, N. las; Lecoeur, M.; Masse, M.; Odou, P.; Simon, N.; Vaccher, C.; Daudet, X.; Richard, D.; Pereira, B.; Clauson, H.; Cueff, R.; Feschet, E.; Breysse, C. Analysis of PVC Plasticizers in Medical Devices and Infused Solutions by GC-MS. *J. Pharm. Biomed. Anal.* **2016**, *118*, 206–213. https://doi.org/10.1016/j.jpba.2015.10.034.

(38) Faessler, D.; McCombie, G.; Biedermann, M.; Felder, F.; Subotic, U. Leaching of Plasticizers from Polyvinylchloride Perfusion Lines by Different Lipid Emulsions for Premature Infants under Clinical Conditions. *Int. J. Pharm.* **2017**, *520* (1–2), 119–125. https://doi.org/10.1016/j.ijpharm.2017.01.046.

(39) Jeon, S. H.; Kim, Y. P.; Kho, Y.; Shin, J. H.; Ji, W. H.; Ahn, Y. G. Development and Validation of Gas Chromatography-Triple Quadrupole Mass Spectrometric Method for Quantitative Determination of Regulated Plasticizers in Medical Infusion Sets. *J. Anal. Methods Chem.* **2018**, *2018*. https://doi.org/10.1155/2018/9470254.

(40) Fernandez-Canal, C.; Pinta, P. G.; Eljezi, T.; Larbre, V.; Kauffmann, S.; Camilleri, L.; Cosserant, B.; Bernard, L.; Pereira, B.; Constantin, J. M.; Grimandi, G.; Sautou, V. Patients' Exposure to PVC Plasticizers from ECMO Circuits. *Expert Rev. Med. Devices* **2018**, *15* (5), 377–383. https://doi.org/10.1080/17434440.2018.1462698.

(41) Den Braver-Sewradj, S. P.; Piersma, A.; Hessel, E. V. S. An Update on the Hazard of and Exposure to Diethyl Hexyl Phthalate (DEHP) Alternatives Used in Medical Devices. *Crit. Rev. Toxicol.* **2020**, *50* (8), 650–672. https://doi.org/10.1080/10408444.2020.1816896.

(42) Rastogi, S. C. Gas Chromatographic Analysis of Phthalate Esters in Plastic Toys. *Chromatographia* **1998**, *47* (11–12), 724–726. https://doi.org/10.1007/BF02467461.

(43) United States Consumer Product Safety Comission (US CPSC). Phthalates and Phthalate Substitutes in Children's Toys https://www.cpsc.gov/s3fs-public/phthallab.pdf (accessed Sep 25, 2023).

(44) Al-Natsheh, M.; Alawi, M.; Fayyad, M.; Tarawneh, I. Simultaneous GC-MS Determination of Eight Phthalates in Total and Migrated Portions of Plasticized Polymeric Toys and Childcare Articles. *J. Chromatogr. B Anal. Technol. Biomed. Life Sci.* **2015**, *985*, 103–109. https://doi.org/10.1016/j.jchromb.2015.01.010.

(45) McCombie, G.; Biedermann, S.; Suter, G.; Biedermann, M. Survey on Plasticizers Currently Found in PVC Toys on the Swiss Market: Banned Phthalates Are Only a Minor Concern. *J. Environ. Sci. Heal. - Part A Toxic/Hazardous Subst. Environ. Eng.* **2017**, *52* (5), 491–496. https://doi.org/10.1080/10934529.2016.1274176.

(46) Ashworth, M.; Chappell, A.; Ashmore, E.; Fowles, J. Analysis and Assessment of Exposure to Selected Phthalates Found in Children's Toys in Christchurch, New Zealand. *Int. J. Environ. Res. Public Health* **2018**, *15* (2), 200. https://doi.org/10.3390/ijerph15020200.

(47) Giovanoulis, G.; Bui, T.; Xu, F.; Papadopoulou, E.; Padilla-Sanchez, J. A.; Covaci, A.; Haug, L. S.; Cousins, A. P.; Magnér, J.; Cousins, I. T.; de Wit, C. A. Multi-Pathway Human Exposure Assessment of Phthalate Esters and DINCH. *Environ. Int.* **2018**, *112* (April 2017), 115–126. https://doi.org/10.1016/j.envint.2017.12.016.

(48) Schettler, T.; Skakkebæk, N. E.; De Kretser, D.; Leffers, H. Human Exposure to Phthalates via Consumer Products. *Int. J. Androl.* **2006**, *29* (1), 134–139. https://doi.org/10.1111/j.1365-2605.2005.00567.x.

(49) Xu, Y.; Cohen Hubal, E. A.; Little, J. C. Predicting Residential Exposure to Phthalate Plasticizer Emitted from Vinyl Flooring: Sensitivity, Uncertainty, and Implications for Biomonitoring. *Environ. Health Perspect.* **2010**, *118* (2), 253–258. https://doi.org/10.1289/ehp.0900559.

(50) Little, J. C.; Weschler, C. J.; Nazaroff, W. W.; Liu, Z.; Cohen Hubal, E. A. Rapid Methods to Estimate Potential Exposure to Semivolatile Organic Compounds in the Indoor Environment. *Environ. Sci. Technol.* **2012**, *46* (20), 11171–11178. https://doi.org/10.1021/es301088a.

(51) Eichler, C. M. A.; Hubal, E. A. C.; Xu, Y.; Cao, J.; Bi, C.; Weschler, C. J.; Salthammer, T.; Morrison, G. C.; Koivisto, A. J.; Zhang, Y.; Mandin, C.; Wei, W.; Blondeau, P.; Poppendieck, D.; Liu, X.; Delmaar, C. J. E.; Fantke, P.; Jolliet, O.; Shin, H. M.; Diamond, M. L.; Shiraiwa, M.; Zuend, A.; Hopke, P. K.; Von Goetz, N.; Kulmala, M.; Little, J. C.

Assessing Human Exposure to SVOCs in Materials, Products, and Articles: A Modular Mechanistic Framework. *Environ. Sci. Technol.* **2021**, *55* (1), 25–43. https://doi.org/10.1021/acs.est.0c02329.

(52) Kim, H.-H.; Yang, J.-Y.; Kim, S.-D.; Yang, S.-H.; Lee, C.-S.; Shin, D.-C.; Lim, Y.-W. Health Risks Assessment in Children for Phthalate Exposure Associated with Childcare Facilities and Indoor Playgrounds. *Environ. Health Toxicol.* **2011**, *26*, e2011008. https://doi.org/10.5620/eht.2011.26.e2011008.

(53) Koch, H. M.; Lorber, M.; Christensen, K. L. Y.; Pälmke, C.; Koslitz, S.; Brüning, T. Identifying Sources of Phthalate Exposure with Human Biomonitoring: Results of a 48h Fasting Study with Urine Collection and Personal Activity Patterns. *Int. J. Hyg. Environ. Health* **2013**, *216* (6), 672–681. https://doi.org/10.1016/j.ijheh.2012.12.002.

(54) Wang, W.; Wu, F. Y.; Huang, M. J.; Kang, Y.; Cheung, K. C.; Wong, M. H. Size Fraction Effect on Phthalate Esters Accumulation, Bioaccessibility and in Vitro Cytotoxicity of Indoor/Outdoor Dust, and Risk Assessment of Human Exposure. *J. Hazard. Mater.* **2013**, *261*, 753–762. https://doi.org/10.1016/j.jhazmat.2013.04.039.

(55) Clausen, P. A.; Liu, Z.; Kofoed-Sørensen, V.; Little, J.; Wolkoff, P. Influence of Temperature on the Emission of Di-(2-Ethylhexyl)Phthalate (DEHP) from PVC Flooring in the Emission Cell FLEC. *Environ. Sci. Technol.* **2012**, *46* (2), 909–915. https://doi.org/10.1021/es2035625.

(56) Lucattini, L.; Poma, G.; Covaci, A.; de Boer, J.; Lamoree, M. H.; Leonards, P. E. G. A Review of Semi-Volatile Organic Compounds (SVOCs) in the Indoor Environment: Occurrence in Consumer Products, Indoor Air and Dust. *Chemosphere* **2018**, *201*, 466–482. https://doi.org/10.1016/j.chemosphere.2018.02.161.

(57) Yang, C.; Harris, S. A.; Jantunen, L. M.; Kvasnicka, J.; Nguyen, L. V.; Diamond, M. L. Phthalates: Relationships between Air, Dust, Electronic Devices, and Hands with Implications for Exposure. *Environ. Sci. Technol.* **2020**, *54* (13), 8186–8197. https://doi.org/10.1021/acs.est.0c00229.

(58) Sukiene, V.; Gerecke, A. C.; Park, Y. M.; Zennegg, M.; Bakker, M. I.; Delmaar, C. J. E.; Hungerbühler, K.; Von Goetz, N. Tracking SVOCs' Transfer from Products to Indoor Air and Settled Dust with Deuterium-Labeled Substances. *Environ. Sci. Technol.* **2016**, *50* (8), 4296–4303. https://doi.org/10.1021/acs.est.5b05906.

(59) Dodson, R. E.; Camann, D. E.; Morello-Frosch, R.; Brody, J. G.; Rudel, R. A. Semivolatile Organic Compounds in Homes: Strategies for Efficient and Systematic Exposure Measurement Based on Empirical and Theoretical Factors. *Environ. Sci. Technol.* **2015**, *49* (1), 113–122. https://doi.org/10.1021/es502988r.

(60) Schossler, P.; Schripp, T.; Salthammer, T.; Bahadir, M. Beyond Phthalates: Gas Phase Concentrations and Modeled Gas/Particle Distribution of Modern Plasticizers. *Sci. Total Environ.* **2011**, *409* (19), 4031–4038. https://doi.org/10.1016/j.scitotenv.2011.06.012.

(61) Garrido, J. A.; Parthasarathy, S.; Moschet, C.; Young, T. M.; McKone, T. E.; Bennett, D. H. Exposure Assessment for Air-To-Skin Uptake of Semivolatile Organic Compounds (SVOCs) Indoors. *Environ. Sci. Technol.* **2019**, *53* (3), 1608–1616. https://doi.org/10.1021/acs.est.8b05123.

(62) Larsson, K.; Lindh, C. H.; Jönsson, B. A.; Giovanoulis, G.; Bibi, M.; Bottai, M.; Bergström, A.; Berglund, M. Phthalates, Non-Phthalate Plasticizers and Bisphenols in Swedish Preschool Dust in Relation to Children's Exposure. *Environ. Int.* **2017**, *102*, 114–124. https://doi.org/10.1016/j.envint.2017.02.006.

(63) Zhang, Q.; Sun, Y.; Zhang, Q.; Hou, J.; Wang, P.; Kong, X.; Sundell, J. Phthalate Exposure in Chinese Homes and Its Association with Household Consumer Products. *Sci. Total Environ.* **2020**, *719*, 136965. https://doi.org/10.1016/j.scitotenv.2020.136965.

(64) Nagorka, R.; Birmili, W.; Schulze, J.; Koschorreck, J. Diverging Trends of Plasticizers (Phthalates and Non-Phthalates) in Indoor and Freshwater Environments—Why? *Environ. Sci. Eur.* **2022**, *34* (1). https://doi.org/10.1186/s12302-022-00620-4.

(65) Guo, Y.; Kannan, K. Comparative Assessment of Human Exposure to Phthalate Esters from House Dust in China and the United States. *Environ. Sci. Technol.* **2011**, *45* (8), 3788–3794. https://doi.org/10.1021/es2002106.

(66) Zhao, A.; Wang, L.; Pang, X.; Liu, F. Phthalates in Skin Wipes: Distribution, Sources, and Exposure via Dermal Absorption. *Environ. Res.* **2022**, *204* (PB), 112041. https://doi.org/10.1016/j.envres.2021.112041.

(67) Rudel, R. A.; Camann, D. E.; Spengler, J. D.; Korn, L. R.; Brody, J. G. Phthalates, Alkylphenols, Pesticides, Polybrominated Diphenyl Ethers, and Other Endocrine-Disrupting Compounds in Indoor Air and Dust. *Environ. Sci. Technol.* **2003**, *37* (20), 4543–4553. https://doi.org/10.1021/es0264596.

(68) Fromme, H.; Schütze, A.; Lahrz, T.; Kraft, M.; Fembacher, L.; Siewering, S.; Burkardt, R.; Dietrich, S.; Koch, H. M.; Völkel, W. Non-Phthalate Plasticizers in German Daycare Centers and Human Biomonitoring of DINCH Metabolites in Children Attending the Centers (LUPE 3). *Int. J. Hyg. Environ. Health* **2016**, *219* (1), 33–39. https://doi.org/10.1016/j.ijheh.2015.08.002.

(69) Nagorka, R.; Conrad, A.; Scheller, C.; Süßenbach, B.; Moriske, H. J. Diisononyl 1,2-Cyclohexanedicarboxylic Acid (DINCH) and Di(2-Ethylhexyl) Terephthalate (DEHT) in Indoor Dust Samples: Concentration and Analytical Problems. *Int. J. Hyg. Environ. Health* **2011**, *214* (1), 26–35. https://doi.org/10.1016/j.ijheh.2010.08.005.

(70) Mansouri, K.; Grulke, C. M.; Judson, R. S.; Williams, A. J. OPERA Models for Predicting Physicochemical Properties and Environmental Fate Endpoints. *J. Cheminform.* **2018**, *10* (1), 1–19. https://doi.org/10.1186/s13321-018-0263-1.

(71) Wang, X.; Song, M.; Guo, M.; Chi, C.; Mo, F.; Shen, X. Pollution Levels and Characteristics of Phthalate Esters in Indoor Air in Hospitals. *J. Environ. Sci. (China)* **2015**, *37*, 67–74. https://doi.org/10.1016/j.jes.2015.02.016.

(72) Zhang, L.; Wang, F.; Ji, Y.; Jiao, J.; Zou, D.; Liu, L.; Shan, C.; Bai, Z.; Sun, Z. Phthalate Esters (PAEs) in Indoor PM10/PM2.5 and Human Exposure to PAEs via Inhalation of Indoor Air in Tianjin, China. *Atmos. Environ.* **2014**, *85*, 139–146. https://doi.org/10.1016/j.atmosenv.2013.11.068.

(73) Peeters, J. R.; Vanegas, P.; Kellens, K.; Wang, F.; Huisman, J.; Dewulf, W.; Duflou, J. R. Forecasting Waste Compositions: A Case Study on Plastic Waste of Electronic Display Housings. *Waste Manag.* **2015**, *46*, 28–39. https://doi.org/10.1016/j.wasman.2015.09.019.

(74) Bui, T. T.; Giovanoulis, G.; Cousins, A. P.; Magnér, J.; Cousins, I. T.; de Wit, C. A. Human Exposure, Hazard and Risk of Alternative Plasticizers to Phthalate Esters. *Sci. Total Environ.* **2016**, *541*, 451–467. https://doi.org/10.1016/j.scitotenv.2015.09.036.

(75) European Commission - Directorate-General for Health and Food Safety. *The Safety of Medical Devices Containing DEHP Plasticized PVC or Other Plasticizers on Neonates and Other Groups Possibly at Risk (2015 Update)*; Brussels, Belgium, 2016; Vol. 76. https://doi.org/10.1016/j.yrtph.2016.01.013.

(76) Panneel, L.; Cleys, P.; Breugelmans, C.; Christia, C.; Malarvannan, G.; Poma, G.; Jorens, P. G.; Mulder, A.; Covaci, A. Neonatal Exposure to Phthalate and Alternative Plasticizers via Parenteral Nutrition. *Int. J. Pharm.* **2023**, *631* (December 2022), 122472. https://doi.org/10.1016/j.ijpharm.2022.122472.
