# Supplementary material for: Legacy and Emerging Plasticizers and Stabilizers in PVC Floorings and Implications for Recycling
Source: Environ Sci Technol. 2024 Jan 19;58(4):1894–907. doi: 10.1021/acs.est.3c04851 (PMC10832040; doi:10.1021/acs.est.3c04851)
Supplement: Supplementary file 3 — es3c04851_si_003.zip [file es3c04851_si_003.zip › Protocol_original-french_Méthode d'analyse des phtalates par GC-MS.pdf]

TABLE DES MATIERES

1 But et domaine d'application 2
2 Responsabilités 2
3 Description 2
3.1 Matériel, instruments et logiciels 2
3.1.1 Matériel usuel 2
3.1.2 Instruments et logiciels 3
3.2 Réactifs, standards et solutions 3
3.2.1 Réactifs usuels 3
3.2.2 Standards et standards internes 3
3.3 Solutions de standards, points de calibration et contrôles qualité 4
3.3.1 Solutions des standards 4
3.3.2 Solution de standards internes 4
3.3.3 Points de calibration 5
3.3.4 Contrôles qualités (QCs) 5
3.4 Paramètres et méthodes instrumentaux 5
3.4.1 Conditions GC 5
3.4.2 Conditions MS 6
3.5 Méthodologie d'analyse 7
3.5.1 Extraction 7
3.5.2 Précipitation 7
3.5.3 Dilution finale 7
3.5.4 Extraction de CRM 7
3.5.5 Séquence d'analyse des échantillons 8
3.6 Calcul des résultats et paramètres de validation 8
3.6.1 Calcul des résultats de concentrations 8
3.6.2 Critères de validité 8
3.6.3 Calcul de la teneur d'un échantillon 9
3.6.4 Règles de décision 9
3.7 Chromatogramme type 10
4 Historique 10
5 Références 10
6 Annexes 10

1 But et domaine d'application

Cette méthode a pour but la détection et quantification de phtalates dans des matériaux (objets) et poussières. Les phtalates sont utilisés comme additifs pour ses propriétés plastifiantes, particulièrement pour les matériaux en PVC.

Selon l'annexe 1.18 de l'ordonnance sur la réduction des risques liés aux produits chimiques (ORRChim), il est interdit de mettre sur le marché des objets contenant les phtalates suivants :

Nom	CAS	Formule	PM
Dibutylphtalate (DBP)	84-74-2	$C_{16}H_{22}O_4$	278.4
Diisobutylphtalate (DiBP)	84-69-5	$C_{16}H_{22}O_4$	278.4
Butylbenzylphtalate (BBP)	85-68-7	$C_{19}H_{20}O_4$	312.4
Bis(2-ethylhexyl)phtalate (DEHP)	117-81-7	$C_{24}H_{38}O_4$	390.6

On considère qu'un objet contient des phtalates si la teneur dépasse 0.1% en masse.

2 Responsabilités

Cette méthode s'adresse à toute personne habilitée du laboratoire qui doit réaliser une quantification de phtalates par GC-MS.

3 Description

3.1 Matériel, instruments et logiciels

3.1.1 Matériel usuel

Dans le cas où le matériel indiqué ci-après n'est pas disponible, il peut éventuellement être remplacé par du matériel équivalent.

<u>Extraction et dilution</u>

- Seringues électronique SGE e-vol^R XR
- Pipettes Pasteur
- Pipettes jaugées de 5 et 10 mL
- Micropipettes Gilson
- Tubes Falcon de 15 mL
- Flacons à vis (verre brun) de 15 mL (Supelco – 27003)
- Flacons à vis (verre brun) de 7 mL (Supelco – 27002-U)
- Flacons à vis (verre brun) de 4 mL (Supelco – 27001-U)
- Flacons à vis (verre brun) de 2 mL (Supelco – 27000)
- Balance de précision Mettler XP 205 DR
- Bain ultrasons
- Centrifugeuse

<u>Injection GCMS</u>

- Flacons de 1.5 mL (amber glass) Screw cap (BGB – 080401-XLW)
- Flacons de 0.2 mL (amber glass) Screw cap (integrated insert) (BGB – 080401-XLE-HP)
- Screw caps with septa silicone, rubber/PTFE (BGB – 090300-B)
- Seringue de 10 µL

3.1.2 Instruments et logiciels

- Chromatographe Agilent 7890A (GC System)
- Détecteur MS Agilent 5975C (inert MSD with Triple-Axis Detector)
- Logiciel Masshunter avec module Gerstel Maestro 1
- Gerstel MPS 2

3.2 Réactifs, standards et solutions

Dans le cas où les standards et solutions indiqués ci-après ne sont pas disponibles, ils peuvent éventuellement être remplacés par des produits équivalents.

3.2.1 Réactifs usuels

- Tetrahydrofurane, Chromosolv®Plus (Sigma-Aldrich / 34865)
- Toluene, puriss.p.a (Sigma-Aldrich / 32249)
- Hexane, GC Plus (Honeywell / 10313896)
- Acétonitrile, Emplura (Merck / 1.15500.2500)

3.2.2 Standards et standards internes

Substance	Fabricant (concentration)	Formule	PM	CAS
**Standards**				
Dimethylphthalate (DMP)	CHIRON (1000 µg/mL dans isooctane)	$C_{10}H_{10}O_4$	194.18	131-11-3
Diethylphthalate (DEP)	CHIRON (1000 µg/mL dans isooctane)	$C_{12}H_{14}O_4$	222.24	84-66-2
Diallylphthalate (DAP)	CHIRON (1000 µg/mL dans isooctane)	$C_{14}H_{14}O_4$	246.26	131-17-9
Diisobutylphthalate (DiBP)	CHIRON (1000 µg/mL dans isooctane)	$C_{16}H_{22}O_4$	278.35	84-69-5
Dibutylphthalate (DBP)	CHIRON (1000 µg/mL dans isooctane)	$C_{16}H_{22}O_4$	278.38	84-74-2
Bis(2-methoxyethyl)phthalate (DMEP)	CHIRON (1000 µg/mL dans isooctane)	$C_{14}H_{18}O_6$	282.32	117-82-8
Diisoamylphthalate (DiPP)	CHIRON (1000 µg/mL dans isooctane)	$C_{18}H_{26}O_4$	306.4	605-50-5
Isopentylpentylphthalate (nPiPP)	CHIRON (1000 µg/mL dans isooctane) ou Synthonix (pur)	$C_{18}H_{26}O_4$	306.4	776297-69-9
Diamylphthalate (DPP)	CHIRON (1000 µg/mL dans isooctane)	$C_{18}H_{26}O_4$	306.4	131-18-0
Butylbenzylphthalate (BBP)	CHIRON (1000 µg/mL dans isooctane)	$C_{19}H_{20}O_4$	312.39	85-68-7
Dihexylphthalate (DHP)	CHIRON (1000 µg/mL dans isooctane)	$C_{20}H_{30}O_4$	334.5	84-75-3
Dicyclohexylphthalate (DCHP)	CHIRON (1000 µg/mL dans isooctane)	$C_{20}H_{26}O_4$	330.42	84-61-7
Bis(2-ethylhexyl)phthalate (DEHP)	CHIRON (1000 µg/mL dans isooctane)	$C_{24}H_{38}O_4$	390.56	117-81-7
Dioctylphthalate (DNOP)	CHIRON (1000 µg/mL dans isooctane)	$C_{24}H_{38}O_4$	390.56	117-84-0
Diisononylphthalate (DiNP)	Sigma-Aldrich (pur)	$C_{26}H_{42}O_4$	418.61	28553-12-0
Diisodecylphthalate (DiDP)	Sigma-Aldrich (pur)	$C_{28}H_{46}O_4$	446.66	26761-40-0
**Standards internes**				
Diisobutylphthalate-d4 (DiBP_d4)	CHIRON (100 ou 1000 µg/mL dans isooctane)	$C_{16}d_4H_{18}O_4$	282.38	358730-88-8
Dibutylphthalate-d4 (DBP_d4)	CHIRON (100 ou 1000 µg/mL dans isooctane)	$C_{16}d_4H_{18}O_4$	282.38	93952-11-5
Diisoamylphthalate-d4 (DiPP_d4)	CHIRON (100 ou 1000 µg/mL dans isooctane)	$C_{18}d_4H_{22}O_4$	310.4	605-50-5

Diamylphthalate-d4 (DPP_d4)	CHIRON (100 ou 1000 µg/mL dans isooctane)	$C_{18}d_4H_{22}O_4$	286.32	358730-89-9
Butylbenzylphthalate-d4 (BBP_d4)	CHIRON (100 ou 1000 µg/mL dans isooctane)	$C_{19}d_4H_{16}O_4$	316.39	93951-88-3
Dihexylphthalate-d4 (DHP_d4)	CHIRON (100 ou 1000 µg/mL dans isooctane)	$C_{20}d_4H_{26}O_4$	338.5	1015854-55-3
Bis(2-ethylhexyl)phthalate-d4 (DEHP_d4)	CHIRON (100 ou 1000 µg/mL dans isooctane)	$C_{24}d_4H_{34}O_4$	394.58	93951-87-2
**Solution mère QC**				
Phthalates Mixture 576 (DiBP, DBP, BBP, DHP, DEHP, DNOP, DiNP, DiDP)	LGC (1000-5000 µg/mL dans dichloromethane)	N/A	N/A	N/A
**CRM (controlled reference material)**				
Phthalates in Polyvinylchloride (CRM-PVC001)	SPEX Certiprep (3000-30000 µg/g)	N/A	N/A	N/A

NB: le fournisseur et la concentration sont indiqués à titre informatif. Des standards similaires peuvent être obtenus chez d'autres fournisseurs à condition qu'il soit accrédité ISO 17034. L'emploi de composé pur (>99%) est possible mais il devra être dissout dans du toluène à une concentration finale d'environ exactement 1000 µg/mL pour la préparation de la solution fille 1. Il sera aussi nécessaire d'introduire précisément les concentrations dans Masshunter pour le calcul de la droite de calibration.

3.3 Solutions de standards, points de calibration et contrôles qualité

Utiliser la seringue eVol pour préparer les solutions intermédiaires, pour les points de la droite de calibration et les QC.

3.3.1 Solutions des standards

Solution intermédiaire DiNP et DiDP

Dans un flacon jaugé de 2mL, peser environ exactement 20 mg de DiDP et DiNP et compléter au trait de jauge avec du toluène.

[DiDP] = [DiNP] = ~10 mg/mL

Pour standards purs

En cas d'utilisation de standards purs, pour chaque composé, peser individuellement environ exactement 10 mg dans un flacon jaugé de 10 mL et compléter au trait de jauge avec du toluène.

[PHT] = 1000 µg/mL

Solution fille 1

Prélever 50 µL de chaque standard à 1000 µg/mL et 50 µL de la solution de DiNP / DiDP à 10 mg/mL, ajouter 250 µL de toluène.

$[Standards]_{SF1}$ = 50 µg/mL et 500 µg/mL (DiNP et DiDP)

Solution fille 2

Prélever 50 µL de solution fille 1 et jauger à 1 mL avec le toluène.

$[Standards]_{SF2}$ = 2.5 µg/mL et 25 µg/mL (DiNP et DiDP)

3.3.2 Solution de standards internes

Prélever 20 µL de chaque standard interne à 1000 µg/mL et/ou 200 µL de chaque standard interne à 100 µg/mL. Compléter à 1 mL avec du toluène.

[Standards interne] = 20 µg/mL

3.3.3 Points de calibration

La droite d'étalonnage est constituée d'au minimum 7 concentrations différentes. Une droite de calibration typique comprend les points de calibration suivants:

Concentration [1] (µg/mL)	Solution fille 1 (µL)	Solution fille 2 (µL)	Solution ISTD (µL)	Tetrahydrofurane (µL)	Volume final (µL)
**10 (100)**	200	-	25	775	**1000**
**5 (50)**	100	-	25	875	**1000**
**1 (10)**	20	-	25	955	**1000**
**0.5 (5)**	-	200	25	775	**1000**
**0.250 (2.5)**	-	100	25	875	**1000**
**0.05 (0.5)**	-	20	25	955	**1000**
**0**	-	-	25	975	**1000**

Chaque point de la droite contient 0.5 µg/mL de standards internes

3.3.4 Contrôle qualité (QC)

Prélever 50 µL de mix de standards 1000 (5000)[1] µg/mL, compléter à 1 mL avec du toluène (950 µL). Prélever ajouter 20 µL de la dernière solution, 25 µL de standard interne et compléter à 1 mL avec de THF (955 µL).

[Standard QC] = 1 (5)[1] µg/mL

3.4 Paramètres et méthodes instrumentaux

La méthode décrite ci-dessous s'applique pour tous les types d'échantillons, les blancs d'injections, les zéros, les standards de calibration et les QC.

Nom de la méthode: PHT_SIM_Date_Version.M

3.4.1 Conditions GC

- Instrument: GC Agilent 7890A (GC System) avec détecteur 5975C (inert MSD with Triple-Axis Detector)
- Colonne: DB-5MS (15m x 250µm x 0,1µm) – Agilent 122-5511
- Pré-colonne: HP-5MS (1m x 250µm x 0,25µm) – Agilent 19091S-433
- Température du four: 80°C, pendant 2 min puis
  20°C/min jusqu'à 200°C puis 8°C/min jusqu'à 320°
- Flow constant helium: 1,5 mL/min
- Pression: 6.1 psi (indicative)
- Injection: mode Pulsed Splitless (70 psi → 1.5min)
- Liner: Topaz, 4mm Single Taper w/Wool, 6.5 x 78.5mm - Restek 23303 (900 µL)
- Température injecteur: 140°C puis 800°C/min jusqu'à 350°C
- Volume injecté: 2 µL
- Méthode de rinçage de la seringue d'injection:

- 3 volumes de seringue avec toluène puis hexane avant injection
- 1 volume avec toluène puis hexane après injection

- Tray cooler: 10°C

[1] Entre parenthèse, la concentration des DiNP et DiDP

- Septum purge flow mode: switched
- Septum purge flow: 3 mL/min
- Purge flow to split vent: 100 mL/min à 2.5 min
- Gas saver: On, 20 mL/min après 4.5 min
- Total flow: 24.5 mL/min (indicatif)
- Durée totale par injection: 23 minutes
- Temps d'équilibrage entre 2 injections: 3 minutes

3.4.2 Conditions MS

- Ligne de transfert: 280°C
- MS Source: 230°C
- MS Quad: 150°C
- Mode : SIM
- Solvent delay: 3.5 minutes
- Résolution: low
- EM Setting: delta EMV (-100V)

Composé	ISTD	RT (min)	Groupe	Période* (min)	Ion quanti (m/z)	Ion contrôle (m/z)	Dwell (ms)
DMP	*DiBP_d4*	4.4	1	3.50 → 5.00	163.0	194.0	100
DEP	*DiBP_d4*	5.61	2	5.00 → 6.50	149.0	177.0	100
DAP	*DiBP_d4*	6.56	3	6.50 → 7.20	149.0	189.0	100
DiBP	*DiBP_d4*	7.23	4	7.20 → 7.70	149.1	223.1	50
DiBP_d4		7.21	4		153.1	227.1	50
DBP	*DBP_d4*	7.7	5	7.70 → 8.00	149.1	223.1	50
DBP_d4		7.7	5		153.1	227.1	50
DMEP	*DiPP_d4*	7.89	6	8.00 → 8.30	59.1	149.0	100
DiPP	*DiPP_d4*	8.23	7	8.30 → 8.55	149.0	237.1	50
DiPP_d4		8.21	7		153.1	241.1	50
nPiPP	*DPP_d4*	8.43	8	8.55 → 8.75	149.0	237.1	100
DPP	*DPP_d4*	8.66	9	8.75 → 9.20	149.1	237.1	50
DPP_d4		8.63	9		153.1	241.1	50
BBP	*BBP_d4*	9.77	10	9.20 → 10.80	206.1	238.0	25
BBP_d4		9.76	10		210.1	242.1	25
DHP	*DHP_d4*	9.78	10		251.1	233.1	25
DHP_d4		9.77	10		255.1	237.1	25
DCHP	*DEHP_d4*	10.86	11	10.80 → 11.35	149.0	167.0	100
DEHP	*DEHP_d4*	11.17	12	11.35 → 11.90	149.0	167.0	50
DEHP_d4		11.13	12		153.1	171.1	50
DNOP	*DEHP_d4*	12.6	13	11.90 → fin	279.1	261.1	33
DiNP	*DEHP_d4*	11.6-15.1	13		293.2	127.1	33
DiDP	*DEHP_d4*	12.7-16.1	13		307.2	289.2	33

* Les périodes peuvent devoir être ré-adaptées lors de changement de colonne ou de pré-colonne.

3.5 Méthodologie d'analyse

3.5.1 Extraction

- Peser environ exactement entre 50 et 500 mg d'échantillon découpé finement ou broyé dans un flacon à vis de 7 ou 15 mL
- Tarer le flacon
- Ajouter entre 5 et 10 mL de tetrahydrofurane à l'aide d'une pipette jaugée
- Placer dans le bain ultrasons (à froid) pendant ~2 heures afin de dissoudre tout ou partie de l'échantillon
- Laisser le flacon revenir à température ambiante et le peser afin de connaître le volume exact de solvant (densité THF: 0.89)
- Dans le cas d'une prise de 500 mg dans 10 mL, une dilution intermédiaire est réalisée en prélevant 500 μL de l'extrait dans 2000 μl de THF (Dilution x5)

Nb: extraire un blanc procédural (THF) et un CRM en même temps que les échantillons

3.5.2 Précipitation

- Mettre 1000 µL d'acétonitrile dans un flacon de 4 mL
- Prélever 500 µL d'extrait et les rajouter
- Placer les flacons au froid (frigo 4°C)
- Laisser reposer ~2 heures afin que la matrice polymérique de l'échantillon précipite et se dépose au fond du flacon
- Si tel n'est pas le cas, transvaser l'échantillon dans un tube Falcon de 15 mL et centrifuger à 8'000 t/min pendant 2 minutes.

3.5.3 Dilution finale

- Prélever, par exemple, 100 µL de surnageant et les mettre dans un flacon de 1 mL
- Ajouter 25 µL de solution de standards internes à 20 µg/mL (toluène)
- Compléter avec 875 µL de THF

Cette dilution est à ajuster en fonction de la quantité de phtalates contenus dans l'échantillon.

Exemple de % massiques obtenus en fonction de la prise et des dilutions:

Prise (mg)	Volume (mL)	Dilution initiale (extrait)	Dilution (précipitation)	Dilution (finale)	Dilution totale	Teneur équivalente (%) à 1μg/mL lu
500	10	5x	3x	5x	75x	0.15
500	5	-	3x	5x	15x	0.015
50	5	-	3x	10x	30x	0.3

3.5.4 Extraction de CRM

L'extraction du CRM suit les étapes 3.5.1 à 3.5.3.

Environ exactement 50 mg d'échantillon sont pesés et dissouts dans 5 mL de tetrahydrofurane.

Dilutions: la dilution intermédiaire dans THF avant précipitation n'est pas nécessaire et la dilution finale est effectuée comme décrite au point 3.5.3 (100 µL de surnageant + 25 µL de SI + 875 µL de THF).

3.5.5 Séquence d'analyse des échantillons

Lors d'analyse d'échantillons une séquence d'analyse complète est réalisée et est constituée au minimum, dans l'ordre, par :

- ✓ 1 blanc instrumental correspondant à une injection de solvant (THF)
- ✓ Une courbe de calibration avec concentrations croissantes incluant un point "zéro" (ISTD seul) ou 1 point de la droite comme QC
- ✓ 1 blanc instrumental correspondant à une injection de solvant (THF)
- ✓ 1 blanc procédural, c'est-à-dire 1 échantillon ne contenant que le solvant et qui suit la procédure d'extraction d'échantillons
- ✓ Echantillons (par série de 10 max.).
- ✓ Entre chaque série d'échantillons 1 QC instrumental.
- ✓ 1 CRM après la dernière série d'échantillons.
- ✓ 1 blanc instrumental correspondant à une injection de solvant (THF)

3.6 Calcul des résultats et paramètres de validation

3.6.1 Calcul des résultats de concentrations

Tous les résultats sont générés par le logiciel MassHunter à travers une feuille de calcul Excel. Chaque chromatogramme est traité *via* un batch par la méthode de quantification "20200114_PHT_Quanti".

Le traitement de l'information peut être résumé en 5 points:

- ✓ Vérification des chromatogrammes et intégrations. Dans le cas où une intégration de pic n'est pas jugée satisfaisante par l'opérateur, une intégration manuelle peut être effectuée.
- ✓ Génération des courbes de calibration pour chaque composé et évaluation de la fonction de réponse (quadratic; Origin: ignore; Weight: 1/x).
- ✓ Traitement de la droite de calibration et QC pour évaluation de la précision et de l'exactitude des résultats. Acceptation ou non de la séquence en fonction des critères de validité.
- ✓ Génération des concentrations mesurées dans les échantillons.
- ✓ Après l'analyse, la séquence de résultats sous forme de batch est sauvée comme suit: Date_PHT_FJ Par exemple: 20191203_PHT_100001

3.6.2 Critères de validité

Courbe de calibration: la courbe de calibration est considérée valide si le coefficient de détermination (r^2) de la fonction de réponse est de 0.99 au minimum. Des points de concentration peuvent éventuellement écartés (outliers) dans la limite de 1 point sur 5, et dans la limite de 1 point par concentration dans le cas de duplicatas.

Lorsque les critères de calibration ne sont pas remplis il faut repréparer une courbe de calibration et réinjecter la séquence. Si le problème persiste il est nécessaire de remplir une action corrective [P03-02-01_F01] et de procéder à une recherche de causes. A noter, lorsqu'un "autotune" est réalisé il est obligatoire de recalibrer le GC avant injections.

Contrôle qualité (QC): un contrôle qualité est considéré valide si la concentration mesurée respecte une erreur relative maximale de ±20%. Lorsque les critères du QC ne sont pas remplis, il faut repréparer le QC et réinjecter la séquence. Si le problème persiste il est nécessaire de remplir une action corrective [P03-02-01_F01] et de procéder à une recherche de causes.

CRM: le CRM est considéré valide si la concentration mesurée respecte une erreur relative maximale de ±20% par rapport au certificat d'analyse sur les phthalates présents dans l'échantillon. Lorsque les critères du CRM ne sont pas remplis, il faut extraire un nouvel échantillon

et réinjecter la séquence. Si le problème persiste il est nécessaire de remplir une action corrective [P03-02-01_F01] et de procéder à une recherche de causes.

Echantillons: pour chaque composé les temps de rétention des ions de quantification et qualification doivent être similaires à ceux des standards internes dans une fenêtre de ±0.1 minute. Le ratio entre ion de quantification et ion de qualification doit être respecté avec une erreur relative maximale de ±20% et conforme au ratio mentionné dans le rapport de validation ou conforme au ratio mesuré pour les standards (pour les composés non validés).

3.6.3 Calcul de la teneur d'un échantillon

Pour calculer la quantité de chaque composé dans l'échantillon, il faut utiliser la formule suivante:

$$t_{phtalate\,[\mu g/g]} = \frac{c_{phtalate\,[\mu g/mL]} \cdot d \cdot V}{m}$$

$t_{phtalate}$= teneur en phtalate dans l'échantillon (µg/g)

c = concentration obtenue en µg/mL (analyse instrumentale)

d = facteur de dilution totale de l'échantillon

V = volume de solubilisation en mL (THF)

m = pesée de l'échantillon en g

3.6.4 Règles de décision

Selon l'annexe 1.18 de l'ordonnance sur la réduction des risques liés aux produits chimiques (ORRChim), il est interdit de mettre sur le marché des objets contenant les phtalates butylbenzylphtalate (BBP), Dibutylphtalate (DBP), Bis(2-ethylhexyl)phtalate (DEHP) et Diisobutylphtalate (DiBP) si lui-même ou une de ses parties présente une teneur en phtalates de 0,1 % masse ou plus dans le matériau contenant le plastifiant.

Le seuil de non-conformité à l'ORRChim est fixé à 0.1% en masse de phtalates sur la masse de matériau homogène. On distingue 3 cas de figures :

Cas 1 : le résultat obtenu, en incluant l'intervalle d'incertitude, est supérieur au seuil de 0.1%. La situation est non-conforme.

Cas 2 : le résultat obtenu, en incluant l'intervalle d'incertitude, inclut la valeur de 0.1%. La situation est considérée comme incertaine vis-à-vis du seuil de décision. La valeur lue est donnée et le risque associé à l'incertitude de mesure est souligné auprès du demandeur.

Cas 3 : le résultat obtenu, en incluant l'intervalle d'incertitude, est inférieur au seuil de 0.1%. La décision est conforme.

La règle de décision est inscrite dans le rapport d'analyse avec le résultat obtenu.

3.7 Chromatogramme type

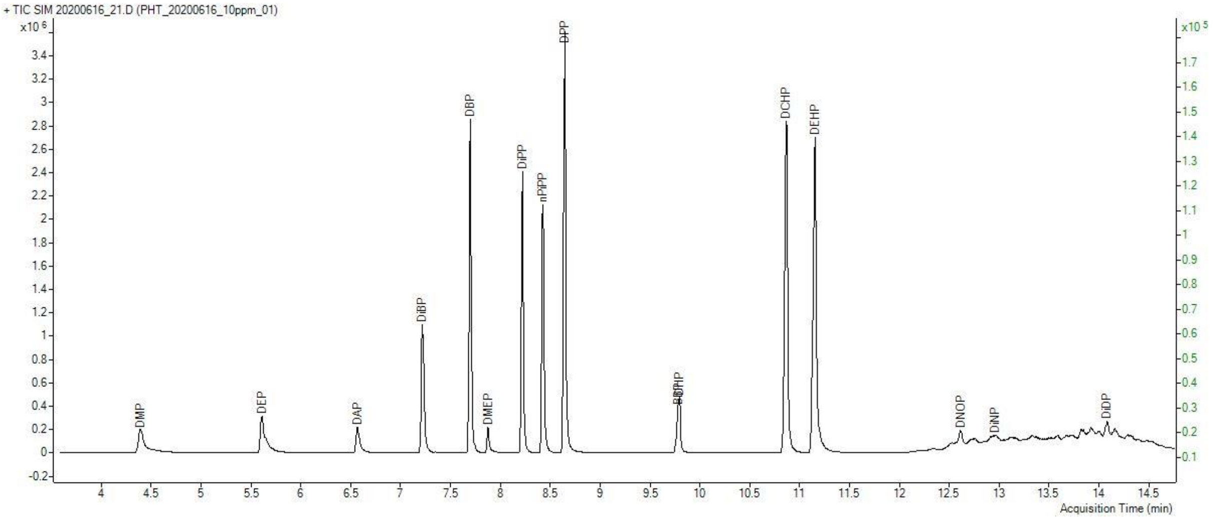

4 Historique

Version 4:

- §3.2.2: modification de la table de standard (utilisation de solution de standard plutôt que de standard pure).
- §3.3.4: remplacement des QCs instrumentaux provenant de la même solution mère que les standards par un mélange de phtalates de provenance différente (indépendance du QC par rapport à la gamme étalon).
- §3.4.1: changement de liner.
- §3.4.2: adaptation des conditions MS (modification des temps/segments du mode SIM en raison d'un changement de colonne). Modification de l'électro-multiplicateur en mode "delta EMV" au lieu de "Absolute EMV" (condition plus stable et indépendant de la perte de sensibilité du détecteur dans le temps).
- §3.4.2: remplacement des ions quanti (149.0 → 59.1) et quali (207.1 → 149.0) du DMEP.
- §3.5.4: ajout de l'analyse d'un CRM.
- §3.5.5: adaptation de la séquence d'analyse (ajout du CRM).

Remarque: ces modifications ont été testées sans impacter la qualité des résultats et ne requièrent pas de nouvelle validation.

5 Références

- Ordonnance sur la réduction des risques liés aux produits chimiques (ORRChim)
- Test Method : CPSC-CH-C1001-09.4 (United States Consumer product safety commission) Standard operating procedure for determination of phthalates, January 17, 2018
- Determination of Phthalate Concentration in Toys and Children’s Products, Agilent application note

6 Annexes

Informations concernant d'autres plastifiants susceptibles d'être présents.

Composé	N° CAS	Formule brute	PM	RT	Ions par ordre décroissant					
Bis(2-ethylhexyl)isophthalate (DOIP)	137-89-3	$C_{24}H_{38}O_4$	390.6	12.14	167.1	149.1	261.1	112.1	279.1	
Bis(2-ethylhexyl) terephthalate (DOTP)	6422-86-2	$C_{24}H_{38}O_4$	390.6	12.65	149.1	261.1	112.2	167.1	279.1	
2,2,4-Trimethyl-1,3-pentanediol diisobutyrate (TXIB)	6846-50-0	$C_{16}H_{30}O_4$	286.4	5.64	111.1	159.1	243.2	155.1	173.1	143.1
Plastic additive 24 CRS (diisononyl cyclohexane-1,2-dicarboxylate) (DINCH)	166412-78-8	$C_{26}H_{48}O_4$	424.7	11.8-13.6	155.1	127.1	207.0	281.3	299.2	252.2
Bis(2-ethylhexyl) adipate (DEHA)	103-23-1	$C_{22}H_{42}O_4$	370.6	10.12	129.1	112.1	147.1	241.2	259.2	
Plastic additive 26 CRS (trioctyl trimellitate) (TOTM)	3319-31-1	$C_{33}H_{54}O_6$	546.8	17.88	305.2	193	323.2	207.1	435.3	417.3

Observations:

Nous retrouvons dans le DOIP et le DOTP les mêmes ions spécifiques (261.1 et 279.1) que dans le DNOP.

De plus le DOTP a exactement le même temps de rétention que le DNOP. Seul le ratio entre les deux ions permet de les différencier.

- DNOP → 279.1/261.1 = ~15
- DOTP → 279.1/261.1 = ~330
-

Le DOTP (à 12.65) et le DOIP (à 12.14) donnent un pic unique dans la fenêtre du DiNP.
