# Supplementary material for: Legacy and Emerging Plasticizers and Stabilizers in PVC Floorings and Implications for Recycling
Source: Environ Sci Technol. 2024 Jan 19;58(4):1894–907. doi: 10.1021/acs.est.3c04851 (PMC10832040; doi:10.1021/acs.est.3c04851)
Supplement: Supplementary file 3 — es3c04851_si_003.zip [file es3c04851_si_003.zip › Protocol_translate-english.pdf]

Subscribe to DeepL Pro to edit this document.
Visit www.DeepL.com/pro for more information.

TABLE OF CONTENTS

1 Purpose and scope 2
2 Responsibilities 2
3 Description 2
3.1 Hardware, instruments and software 2
3.1.1 Standard equipment 2
3.1.2 Instruments and software 2
3.2 Reagents, standards and solutions 3
3.2.1 Common reagents 3
3.2.2 Internal standards 3
3.3 Standard solutions, calibration points and quality controls 4
3.3.1 Standard solutions 4
3.3.2 Internal standards solution 4
3.3.3 Calibration points 5
3.3.4 Quality controls (QCs) 5
3.4 Instrumental parameters and methods 5
3.4.1 GC conditions 5
3.4.2 MS conditions 6
3.5 Analysis methodology 7
3.5.1 Extraction 7
3.5.2 Precipitation 7
3.5.3 Final dilution 7
3.5.4 CRM extraction 7
3.5.5 Sample analysis sequence 8
3.6 Calculation of results and validation parameters 8
3.6.1 Calculation of concentration results 8
3.6.2 Validity criteria 8
3.6.3 Calculating the content of a sample 9
3.6.4 Decision rules 9
3.7 Typical chromatogram 10
4 History 10
5 References 10
6 Appendices 10

1 Purpose and scope

The aim of this method is to detect and quantify phthalates in materials (objects) and dusts. Phthalates are used as additives for their plasticizing properties, particularly in PVC materials.

According to Annex 1.18 of the Ordinance on Risk Reduction related to Chemical Products (ORRChem), it is forbidden to place on the market articles containing the following phthalates:

Name	CASE	Formula	PM
Dibutylphthalate (DBP)	84-74-2	C H O_{16224}	278.4
Diisobutylphthalate (DiBP)	84-69-5	C H O_{16224}	278.4
Butylbenzylphthalate (BBP)	85-68-7	C H O_{19204}	312.4
Bis(2-ethylhexyl)phthalate (DEHP)	117-81-7	C H O_{24384}	390.6

An object is considered to contain phthalates if the content exceeds 0.1% by mass.

2 Responsibilities

This method is intended for all authorized laboratory personnel who need to quantify phthalates by GC-MS.

3 Description

3.1 Hardware, instruments and software

3.1.1 Usual equipment

If the equipment listed below is not available, it may be replaced by equivalent equipment.

Extraction and dilution

- Electronic syringes SGE e-vol^{R} XR
- Pasteur pipettes
- 5 and 10 mL volumetric pipettes
- Gilson micropipettes
- 15 mL Falcon tubes
- 15 mL screw-top vials (brown glass) (Supelco - 27003)
- 7 mL screw-top vials (brown glass) (Supelco - 27002-U)
- 4 mL screw-top vials (brown glass) (Supelco - 27001-U)
- 2 mL screw-top vials (brown glass) (Supelco - 27000)
- Precision balance Mettler XP 205 DR
- Ultrasonic bath
- Centrifuge

GCMS injection

- 1.5 mL vials (amber glass) Screw cap (BGB - 080401-XLW)
- 0.2 mL vials (amber glass) Screw cap (integrated insert) (BGB - 080401-XLE-HP)
- Screw caps with septa silicone, rubber/PTFE (BGB - 090300-B)
- 10 µL syringe

3.1.2 Instruments and software

- Agilent 7890A chromatograph (GC System)

- Agilent 5975C MS Detector (inert MSD with Triple-Axis Detector)
- Masshunter software with Gerstel Maestro 1 module
- Gerstel MPS 2

3.2 Reagents, standards and solutions

If the standards and solutions listed below are not available, they may be replaced by equivalent products.

3.2.1 Common reagents

- Tetrahydrofuran, Chromosolv®Plus (Sigma-Aldrich / 34865)
- Toluene, puriss.p.a (Sigma-Aldrich / 32249)
- Hexane, GC Plus (Honeywell / 10313896)
- Acetonitrile, Emplura (Merck / 1.15500.2500)

3.2.2 Internal standards

Substance	Manufacturer (concentration)	Formula	PM	CASE
**Standards**				
Dimethylphthalate (DMP)	CHIRON (1000 µg/mL in isooctane)	C H O_{10104}	194.18	131-11-3
Diethylphthalate (DEP)	CHIRON (1000 µg/mL in isooctane)	C H O_{12144}	222.24	84-66-2
Diallylphthalate (DAP)	CHIRON (1000 µg/mL in isooctane)	C H O_{14144}	246.26	131-17-9
Diisobutylphthalate (DiBP)	CHIRON (1000 µg/mL in isooctane)	C H O_{16224}	278.35	84-69-5
Dibutylphthalate (DBP)	CHIRON (1000 µg/mL in isooctane)	C H O_{16224}	278.38	84-74-2
Bis(2-methoxyethyl)phthalate (DMEP)	CHIRON (1000 µg/mL in isooctane)	C H O_{14186}	282.32	117-82-8
Diisoamylphthalate (DiPP)	CHIRON (1000 µg/mL in isooctane)	C H O_{18264}	306.4	605-50-5
Isopentylpentylphthalate (nPiPP)	CHIRON (1000 µg/mL in isooctane) or Synthonix (pure)	C H O_{18264}	306.4	776297-69-9
Diamylphthalate (DPP)	CHIRON (1000 µg/mL in isooctane)	C H O_{18264}	306.4	131-18-0
Butylbenzylphthalate (BBP)	CHIRON (1000 µg/mL in isooctane)	C H O_{19204}	312.39	85-68-7
Dihexylphthalate (DHP)	CHIRON (1000 µg/mL in isooctane)	C H O_{20304}	334.5	84-75-3
Dicyclohexylphthalate (DCHP)	CHIRON (1000 µg/mL in isooctane)	C H O_{20264}	330.42	84-61-7
Bis(2-ethylhexyl)phthalate (DEHP)	CHIRON (1000 µg/mL in isooctane)	C H O_{24384}	390.56	117-81-7
Dioctylphthalate (DNOP)	CHIRON (1000 µg/mL in isooctane)	C H O_{24384}	390.56	117-84-0
Diisononylphthalate (DiNP)	Sigma-Aldrich (pure)	C H O_{26424}	418.61	28553-12-0
Diisodecylphthalate (DiDP)	Sigma-Aldrich (pure)	C H O_{28464}	446.66	26761-40-0
**Internal standards**				
Diisobutylphthalate-d4 (DiBP_d4)	CHIRON (100 or 1000 µg/mL in isooctane)	C d H O_{164184}	282.38	358730-88-8
Dibutylphthalate-d4 (DBP_d4)	CHIRON (100 or 1000 µg/mL in isooctane)	C d H O_{164184}	282.38	93952-11-5
Diisoamylphthalate-d4 (DiPP_d4)	CHIRON (100 or 1000 µg/mL in isooctane)	C d H O_{184224}	310.4	605-50-5
Diamylphthalate-d4 (DPP_d4)	CHIRON (100 or 1000 µg/mL in isooctane)	C d H O_{184224}	286.32	358730-89-9
Butylbenzylphthalate-d4 (BBP_d4)	CHIRON (100 or 1000 µg/mL in isooctane)	C d H O_{194164}	316.39	93951-88-3

Dihexylphthalate-d4 (DHP_d4)	CHIRON (100 or 1000 µg/mL in isooctane)	C d H O_{204264}	338.5	1015854-55-3
Bis(2-ethylhexyl)phthalate-d4 (DEHP_d4)	CHIRON (100 or 1000 µg/mL in isooctane)	C d H O_{244344}	394.58	93951-87-2
**QC stock solution**				
Phthalates Mixture 576 (DiBP, DBP, BBP, DHP, DEHP, DNOP, DiNP, DiDP)	LGC (1000-5000 µg/mL in dichloromethane)	N/A	N/A	N/A
**CRM (controlled reference material)**				
Phthalates in Polyvinylchloride (CRM-PVC001)	SPEX Certiprep (3000-30000 µg/g)	N/A	N/A	N/A

NB: Supplier and concentration are given for information only. Similar standards can be obtained from other suppliers, provided they are ISO 17034 accredited. Pure compound (>99%) can be used, but must be dissolved in toluene to a final concentration of exactly 1000 µg/mL for preparation of daughter solution 1. It will also be necessary to accurately enter the concentrations into Masshunter to calculate the calibration line.

3.3 Solutions for standards, calibration points and quality control

Use the eVol syringe to prepare intermediate solutions for calibration line points and QCs.

3.3.1 Standard solutions

DiNP and DiDP intermediate solution

In a 2mL volumetric flask, weigh out exactly 20 mg of DiDP and DiNP and make up to the mark with toluene.

[DiDP] = [DiNP] = ~10 mg/mL

For pure standards

When using pure standards, for each compound, individually weigh exactly 10 mg into a 10 mL volumetric flask and make up to the mark with toluene.

[PHT] = 1000 µg/mL

Daughter solution 1

Take 50 µL of each standard at 1000 µg/mL and 50 µL of DiNP / DiDP solution at 10 mg/mL, add 250µ L toluene.

$[Standards]_{SF1}$ = 50 µg/mL and 500 µg/mL (DiNP and DiDP)

Daughter solution 2

Take 50 µL of daughter solution 1 and make up to 1 mL with toluene.

$[Standards]_{SF2}$ = 2.5 µg/mL and 25 µg/mL (DiNP and DiDP)

3.3.2 Internal standards solution

Take 20 µL of each internal standard at 1000 µg/mL and/or 200 µL of each internal standard at 100 µg/mL. Make up to 1 mL with toluene.

[Internal standard] = 20 µg/mL

3.3.3 Calibration points

The calibration line consists of at least 7 different concentrations. A typical calibration line comprises the following calibration points:

Concentration [1] (µg/mL)	Daughter solution 1 (µL)	Daughter solution 2 (µL)	ISTD solution (µL)	Tetrahydrofuran (µL)	Final volume (µL)
**10 (100)**	200	-	25	775	**1000**
**5 (50)**	100	-	25	875	**1000**
**1 (10)**	20	-	25	955	**1000**
**0.5 (5)**	-	200	25	775	**1000**
**0.250 (2.5)**	-	100	25	875	**1000**
**0.05 (0.5)**	-	20	25	955	**1000**
**0**	-	-	25	975	**1000**

Each point on the line contains 0.5 µg/mL of internal standards

3.3.4 Quality Control (QC)

Take 50 µL of standard mix 1000 (5000)[1] µg/mL, make up to 1 mL with toluene (950 µL). Add 20 µL of the last solution, 25 µL of internal standard and make up to 1 mL with THF (955 µL).

[Standard QC] = 1 (5)[1] µg/mL

3.4 Instrumental parameters and methods

The method described below applies to all sample types, injection blanks, zeros, calibration standards and QCs.

Method name: PHT_SIM_Date_Version.M

3.4.1 GC conditions

- Instrument: GC Agilent 7890A (GC System) with 5975C detector (inert MSD with Triple-Axis Detector)
- Column: DB-5MS (15m x 250µm x 0.1µm) - Agilent 122-5511
- Pre-column: HP-5MS (1m x 250µm x 0.25µm) - Agilent 19091S-433
- Oven temperature: 80°C, for 2 min then

  20°C/min up to 200°C then 8°C/min up to 320°.
- Flow constant helium: 1.5 mL/min
- Pressure: 6.1 psi (indicative)
- Injection: Pulsed Splitless mode (70 psi → 1.5min)
- Liner: Topaz, 4mm Single Taper w/Wool, 6.5 x 78.5mm - Restek 23303 (900 µL)
- Injector temperature: 140°C then 800°C/min to 350°C
- Volume injected: 2 µL
- Injection syringe rinsing method:
  - 3 syringe volumes with toluene then hexane before injection
  - 1 volume with toluene then hexane after injection
- Tray cooler: 10°C
- Septum purge flow mode: switched

[1] In parenthesis, the concentration of DiNP and DiDP

- Septum purge flow: 3 mL/min
- Purge flow to split vent: 100 mL/min à 2.5 min
- Gas saver: On, 20 mL/min after 4.5 min
- Total flow: 24.5 mL/min (indicative)
- Total time per injection: 23 minutes
- Equilibration time between 2 injections: 3 minutes

3.4.2 MS conditions

- Transfer line: 280°C
- MS Source: 230°C
- MS Quad: 150°C
- Mode: SIM
- Solvent delay: 3.5 minutes
- Resolution: low
- EM Setting: delta EMV (-100V)

Compound	ISTD	RT (min)	Group	Period* (min)	Quantile ion (m/z)	Control ion (m/z)	Dwell (ms)
DMP	*DiBP_d4*	4.4	1	3.50 → 5.00	163.0	194.0	100
DEP	*DiBP_d4*	5.61	2	5.00 → 6.50	149.0	177.0	100
DAP	*DiBP_d4*	6.56	3	6.50 → 7.20	149.0	189.0	100
DiBP	*DiBP_d4*	7.23	4	7.20 → 7.70	149.1	223.1	50
DiBP_d4		7.21	4		153.1	227.1	50
DBP	*DBP_d4*	7.7	5	7.70 → 8.00	149.1	223.1	50
DBP_d4		7.7	5		153.1	227.1	50
DMEP	*DiPP_d4*	7.89	6	8.00 → 8.30	59.1	149.0	100
DiPP	*DiPP_d4*	8.23	7	8.30 → 8.55	149.0	237.1	50
DiPP_d4		8.21	7		153.1	241.1	50
nPiPP	*DPP_d4*	8.43	8	8.55 → 8.75	149.0	237.1	100
PLR	*DPP_d4*	8.66	9	8.75 → 9.20	149.1	237.1	50
DPP_d4		8.63	9		153.1	241.1	50
BBP	*BBP_d4*	9.77	10	9.20 → 10.80	206.1	238.0	25
BBP_d4		9.76	10		210.1	242.1	25
DHP	*DHP_d4*	9.78	10		251.1	233.1	25
DHP_d4		9.77	10		255.1	237.1	25
DCHP	*DEHP_d4*	10.86	11	10.80 → 11.35	149.0	167.0	100
DEHP	*DEHP_d4*	11.17	12	11.35 → 11.90	149.0	167.0	50
DEHP_d4		11.13	12		153.1	171.1	50
DNOP	*DEHP_d4*	12.6	13	11.90 → end	279.1	261.1	33
DiNP	*DEHP_d4*	11.6-15.1	13		293.2	127.1	33
DiDP	*DEHP_d4*	12.7-16.1	13		307.2	289.2	33

* Periods may have to be readjusted when changing column or pre-column.

3.5 Analysis methodology

3.5.1 Extraction

- Weigh approximately exactly 50 to 500 mg of finely cut or ground sample into a 7 or 15 mL screw-top flask.
- Tare the bottle
- Add 5-10 mL tetrahydrofuran using a volumetric pipette
- Place in ultrasonic bath (cold) for ~2 hours to dissolve all or part of the sample
- Return the bottle to room temperature and weigh it to determine the exact volume of solvent (THF density: 0.89).
- In the case of an intake of 500 mg in 10 mL, an intermediate dilution is made by taking 500 □L of the extract in 2000 □l of THF (Dilution x5).

Nb: extract a procedural blank (THF) and a CRM at the same time as the samples

3.5.2 Precipitation

- Place 1000 µL acetonitrile in a 4 mL vial
- Remove 500 µL of extract and add
- Place bottles in the fridge (4°C).
- Allow to stand for ~2 hours, so that the sample's polymer matrix precipitates and settles to the bottom of the vial.
- If this is not the case, transfer the sample to a 15 mL Falcon tube and centrifuge at 8,000 rpm for 2 minutes.

3.5.3 Final dilution

- Take, for example, 100 µL of supernatant and place in a 1 mL vial.
- Add 25 µL of 20 µg/mL internal standard solution (toluene)
- Complete with 875 µL THF

This dilution should be adjusted according to the quantity of phthalates in the sample.

Example of mass % obtained according to setting and dilutions:

Intake (mg)	Volume (mL)	Initial dilution (excerpt)	Dilution (precipitation)	Dilution (final)	Total dilution	Equivalent content (%) at 1□g/mL lu
500	10	5x	3x	5x	75x	0.15
500	5	-	3x	5x	15x	0.015
50	5	-	3x	10x	30x	0.3

3.5.4 CRM extraction

CRM extraction follows the steps below 3.5.1 à 3.5.3.

Approximately 50 mg of sample is weighed and dissolved in 5 mL tetrahydrofuran.

Dilutions: intermediate dilution in THF prior to precipitation is not necessary and final dilution is carried out as described in section 3.5.3 (100 µL supernatant + 25 µL SI + 875 µL THF).

3.5.5 Sample analysis sequence

When analyzing samples, a complete analysis sequence is performed, consisting of at least, in order, :

- ✓ 1 instrumental blank corresponding to solvent injection (THF)
- ✓ A calibration curve with increasing concentrations including a "zero" point (ISTD only) or 1 point of the line as QC
- ✓ 1 instrumental blank corresponding to solvent injection (THF)
- ✓ 1 procedural blank, i.e. 1 sample containing only the solvent and following the sample extraction procedure
- ✓ Samples (max. 10 per set).
- ✓ Between each series of samples 1 QC instrumental.
- ✓ 1 CRM after the last series of samples.
- ✓ 1 instrumental blank corresponding to solvent injection (THF)

3.6 Calculation of results and validation parameters

3.6.1 Calculation of concentration results

All results are generated by MassHunter software via an Excel spreadsheet. Each chromatogram is batch-processed *using* the "20200114_PHT_Quanti" quantification method.

Information processing can be summarized in 5 points:

- ✓ Checking chromatograms and integrations. If a peak integration is not deemed satisfactory by the operator, a manual integration can be performed.
- ✓ Generation of calibration curves for each compound and evaluation of response function (quadratic; Origin: ignore; Weight: 1/x).
- ✓ Processing of calibration line and QC to assess precision and accuracy of results. Accept or reject the sequence according to validity criteria.
- ✓ Generation of measured sample concentrations.
- ✓ After analysis, the sequence of results in batch form is saved as follows:
  Date_PHT_FJ For example: 20191203_PHT_100001

3.6.2 Validity criteria

<u>Calibration curve</u>: the calibration curve is considered valid if the coefficient of determination (r^2) of the response function is at least 0.99. Concentration points may be excluded (outliers) up to a limit of 1 point out of 5, and up to a limit of 1 point per concentration in the case of duplicates.

If the calibration criteria are not met, a new calibration curve must be prepared and the sequence re-injected. If the problem persists, complete a corrective action [P03-02-01_F01] and investigate the causes. Note that when an "autotune" is performed, the GC must be recalibrated before injections.

<u>Quality control (QC)</u>: QC is considered valid if the measured concentration is within a maximum relative error of ±20%. If the QC criteria are not met, the QC must be repeated and the sequence re-injected. If the problem persists, a corrective action [P03-02-01_F01] must be completed and the causes investigated.

<u>CRM</u>: the CRM is considered valid if the measured concentration respects a maximum relative error of ±20% compared with the certificate of analysis for phthalates present in the sample. If the CRM criteria are not met, a new sample must be extracted and the sequence reinjected. If the problem persists, a corrective action [P03-02-01_F01] must be completed and the causes investigated.

Samples: for each compound, the retention times of the quantification and qualification ions must be similar to those of the internal standards within a window of ±0.1 minute. The ratio between quantification ion and qualification ion must be respected with a maximum relative error of ±20% and conform to the ratio mentioned in the validation report or conform to the ratio measured for standards (for non-validated compounds).

3.6.3 Sample content calculation

To calculate the quantity of each compound in the sample, use the following formula:

$$t_{phtalate\,[\mu g/g]} = \frac{c_{phtalate\,[\mu g/mL]} \cdot d \cdot V}{m}$$

tphthalate= phthalate content in sample (µg/g)

c = concentration obtained in µg/mL (instrumental analysis)

d = total sample dilution factor

V = solubilization volume in mL (THF)

m = sample weight in g

3.6.4 Decision rules

According to Annex 1.18 of the Ordinance on Risk Reduction related to Chemical Products (ORRChem), it is forbidden to place on the market articles containing the phthalates butylbenzylphthalate (BBP), dibutylphthalate (DBP), bis(2-ethylhexyl)phthalate (DEHP) and diisobutylphthalate (DiBP) if the article itself or any of its parts contains a phthalate content of 0.1% mass or more in the material containing the plasticizer.

The threshold for non-compliance with the ORRChem is set at 0.1% by mass of phthalates in the homogeneous material. There are 3 possible scenarios:

Case 1: the result obtained, including the uncertainty interval, is above the 0.1% threshold. The situation is non-compliant.

Case 2: the result obtained, including the uncertainty interval, includes the 0.1% value. The situation is considered uncertain in relation to the decision threshold. The value read is given and the risk associated with the measurement uncertainty is highlighted to the applicant.

Case 3: the result obtained, including the uncertainty interval, is below the 0.1% threshold. The decision is compliant.

The decision rule is recorded in the analysis report along with the result obtained.

3.7 Typical chromatogram

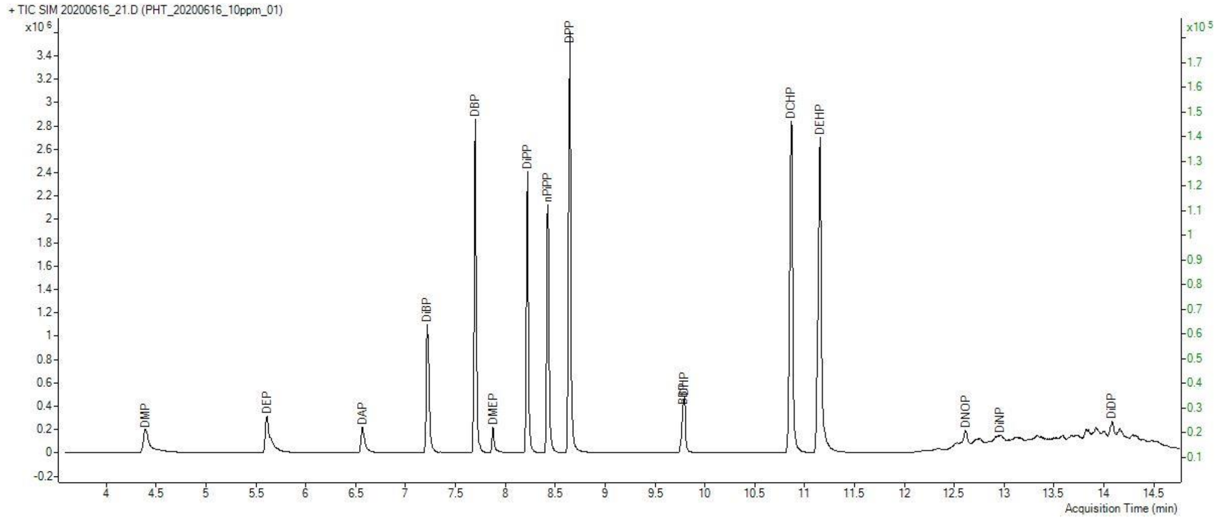

4 History

Version 4:

- §3.2.2Standard table modification (use of standard solution rather than pure standard).
- §3.3.4replacement of instrumental QCs from the same stock solution as the standards by a mixture of phthalates from different sources (independence of the QC from the standard range).
- §3.4.1Liner change.
- §3.4.2Adaptation of MS conditions (modification of SIM mode times/segments due to column change). Modification of electro-multiplier to "delta EMV" mode instead of "Absolute EMV" (more stable condition and independent of loss of detector sensitivity over time).
- §3.4.2replacement of quanti (149.0 → 59.1) and quali (207.1 → 149.0) DMEP ions.
- §3.5.4CRM analysis added.
- §3.5.5adaptation of the analysis sequence (addition of CRM).

Note: these modifications have been tested without impacting the quality of the results and do not require further validation.

5 References

- Ordinance on Risk Reduction related to Chemical Products (ORRChem)
- Test Method : CPSC-CH-C1001-09.4 (United States Consumer product safety commission) Standard operating procedure for determination of phthalates, January 17, 2018
- Determination of Phthalate Concentration in Toys and Children's Products, Agilent application note

6 Appendices

Information on other plasticizers that may be present.

Compound	CAS NO.	Gross formula	PM	RT	Ions in descending order					
Bis(2-ethylhexyl)isophthalate (DOIP)	137-89-3	C H O$_{24384}$	390.6	12.14	167.1	149.1	261.1	112.1	279.1	
Bis(2-ethylhexyl) terephthalate (DOTP)	6422-86-2	C H O$_{24384}$	390.6	12.65	149.1	261.1	112.2	167.1	279.1	
2,2,4-Trimethyl-1,3-pentanediol diisobutyrate (TXIB)	6846-50-0	C H O$_{16304}$	286.4	5.64	111.1	159.1	243.2	155.1	173.1	143.1
Plastic additive 24 CRS (diisononyl cyclohexane-1,2-dicarboxylate) (DINCH)	166412-78-8	C H O$_{26484}$	424.7	11.8-13.6	155.1	127.1	207.0	281.3	299.2	252.2
Bis(2-ethylhexyl) adipate (DEHA)	103-23-1	C H O$_{22424}$	370.6	10.12	129.1	112.1	147.1	241.2	259.2	
Plastic additive 26 CRS (trioctyl trimellitate) (TOTM)	3319-31-1	C H O$_{33546}$	546.8	17.88	305.2	193	323.2	207.1	435.3	417.3

Comments:

We find the same specific ions (261.1 and 279.1) in DOIP and DOTP as in DNOP.

What's more, DOTP has exactly the same retention time as DNOP. Only the ratio between the two ions differentiates them.

- DNOP → 279.1/261.1 = ~15
- DOTP → 279.1/261.1 = ~330
-

DOTP (at 12.65) and DOIP (at 12.14) give a single peak in the DiNP window.
